# Urea hydrogen-bond donor strengths: bigger is not always better†‡

**DOI:** 10.1039/d4cp04042b

**Published:** 2024-12-05

**Authors:** Celine Nieuwland, Angelina N. van Dam, F. Matthias Bickelhaupt, Célia Fonseca Guerra

**Affiliations:** a Department of Chemistry and Pharmaceutical Sciences, Amsterdam Institute of Molecular and Life Sciences (AIMMS), Vrije Universiteit Amsterdam De Boelelaan 1108 1081 HZ Amsterdam The Netherlands c.fonsecaguerra@vu.nl https://www.theochem.nl/; b Institute for Molecules and Materials, Radboud University Heyendaalseweg 135 6525 AJ Nijmegen The Netherlands; c Department of Chemical Sciences, University of Johannesburg, Auckland Park Johannesburg 2006 South Africa

## Abstract

The hydrogen-bond donor strength of ureas, widely used in hydrogen-bond donor catalysis, molecular recognition, and self-assembly, can be enhanced by increasing the size of the chalcogen X in the C=X bond from O to S to Se and by introducing more electron-withdrawing substituents because both modifications increase the positive charge on the NH groups which become better hydrogen-bond donors. However, in 1,3-diaryl X-ureas, a steric mechanism disrupts the positive additivity of these two tuning factors, as revealed by our quantum-chemical analyses. This leads to an enhanced hydrogen-bond donor strength, despite a lower NH acidity, for 1,3-diaryl substituted O-ureas compared to the S- and Se-urea analogs. In addition, we provide a strategy to overcome this steric limitation using a predistorted urea-type hydrogen-bond donor featuring group 14 elements in the C=X bond so that the hydrogen-bond donor strength of X-urea derivatives bearing two aryl substituents can be enhanced upon varying X down group 14.

## Abstract 2

Urea hydrogen-bond donor strengths can be enhanced by electron-withdrawing substituents and a larger X in the C=X bond. However, our quantum-chemical analyses reveal a steric mechanism disrupting this additivity, and a strategy to restore it.

## Graphical abstract

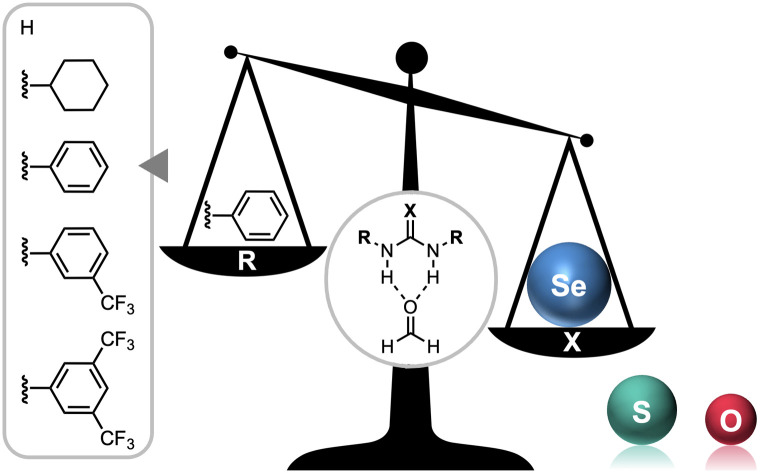

## Introduction

**Fig. 1:**
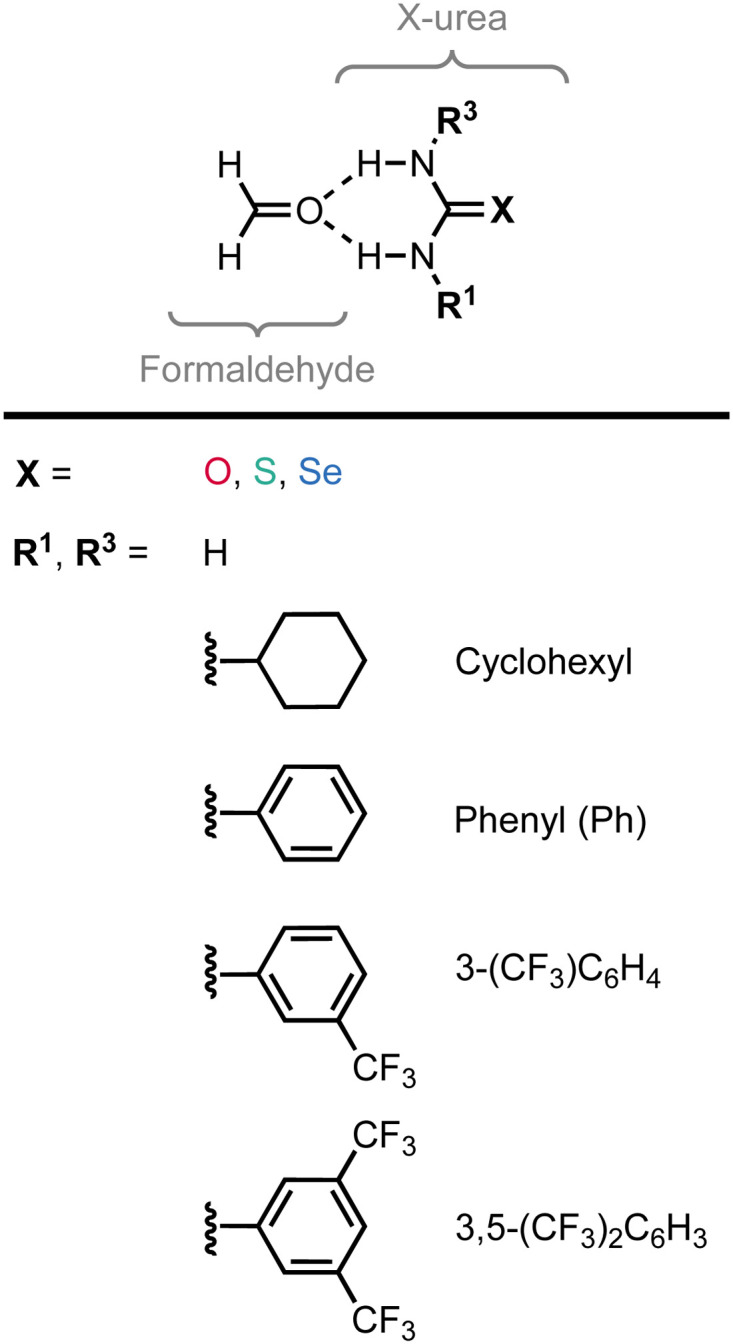
The formaldehyde⋯X-urea hydrogen-bonded complexes studied in this work with X = O (urea), S (thiourea), and Se (selenourea) and different 1,3-substituents (R^1^ and R^3^).

Inspired by the structure and function of natural proteins, intermolecular hydrogen bonding involving amides is nowadays widely used in the field of supramolecular chemistry.^1,2^ Owing to their bidentate nature, with two amino NH groups, urea derivatives have gained significant popularity as hydrogen-bond donor agents in many supramolecular applications, including hydrogen-bond donor organocatalysis,^3^ molecular and anion recognition,^4^ and self-assembly (Fig. 1).^5^ Besides ureas (*i.e.*, involving oxygen in the C=X bond), thioureas (*i.e.*, urea comprising sulfur in the C=X bond) have also attracted considerable attention, as the sulfur analogs are experimentally found to be more acidic compared to ureas.^6^ The higher acidity, that is, the ease of deprotonation of the (N–)H proton, means that thioureas are intrinsically stronger hydrogen-bond donors than ureas because both phenomena relate to the ability of the NH group to interact with and accept electronic charge from a (Lewis) base. The enhanced hydrogen-bond ability of thioureas may seem counterintuitive because the lower electronegativity of sulfur compared to oxygen suggests, erroneously, a reduced C=X group electronegativity and thus a reduced NH hydrogen-bond donor strength of thioureas compared to ureas. Recently, we have revealed through quantum-chemical bonding analyses why the NH hydrogen-bond donor strength of ureas is enhanced by exchanging oxygen in the C=O bond for the less electronegative group 16 elements S or Se.^7–10^ We found that the steric size of the chalcogen atom X, not its electronegativity, is at the origin of the experimentally observed enhanced hydrogen-bond donor strength of thioureas (X = S) and selenoureas (X = Se) compared to ureas (X = O).^7^ Furthermore, we have shown that this trend in hydrogen-bond donor strength and the steric mechanism behind it is not exclusive to group 16. In fact, it can be generalized to varying X in the amide C=X bond down groups 14 and 15 of the periodic table.^9^ Thus, a larger atom X pushes the C=X bond to a longer equilibrium distance at which the 
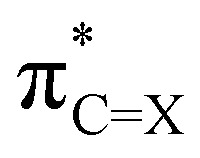
 lowest-unoccupied molecular orbital (LUMO) drops in energy and becomes a better electron acceptor.^7,9^ This makes the C=X group effectively more electronegative upon varying X down a group in the sense that it can accommodate more charge of the lone pairs on the NH groups which, therefore, become more positively charged and thus better hydrogen-bond donors. Note that the higher effective electronegativity of the C=S group also explains the higher experimental acidity (*i.e.*, lower p*K*_a_) of thioureas compared to ureas because it can more effectively stabilize the negative charge upon removal of the (N–)H proton than a C=O group.

In addition to the chalcogen atom in the urea C=X bond, the substituents attached to the NH groups can alter the acidity of (thio)ureas. It is experimentally shown that increasing the electron-withdrawing nature of the substituents in 1,3-substituted (thio)urea hydrogen-bond donors, from cyclohexyl to phenyl to trifluoromethyl (CF_3_)-substituted phenyl, lowers the p*K*_a_ and thus increases the acidic strength of the (thio)urea analog.^6^

The experimental identification of two distinct mechanisms for enhancing the acidity of chalcogenide ureas inspired us to investigate how these two methods can be used in concert to tune the urea hydrogen-bond donor strength, that is (i) upon changing the chalcogen X in the C=X bond from O to S to Se and (ii) by introducing increasingly electron-withdrawing substituents. To this end, we have explored the structure, stability, and bonding of model hydrogen-bonded formaldehyde⋯X-urea complexes (Fig. 1) using dispersion-corrected, relativistic density functional theory (DFT) computations at the ZORA^11^-BLYP^12^-D3(BJ)^13^/TZ2P level of theory using the Amsterdam density functional (ADF)^14^ program as implemented in the Amsterdam modeling suite (AMS) (see ESI,‡ Method S1 for full computational details). The formaldehyde⋯X-urea complexes comprise two intermolecular hydrogen bonds, the so-called bifurcated hydrogen bonds. Herein, we varied systematically the chalcogen X in the urea C=X bond from O to S to Se and screened the effect of introducing 1,3-substituents (R^1^ and R^3^) to the unsubstituted X-ureas (R^1^,R^3^ = H) with increasingly electron-withdrawing nature from cyclohexyl to phenyl (Ph) to 3-(CF_3_)-phenyl to 3,5-(CF_3_)_2_-phenyl. We show that increasing the size of the chalcogen in the C=X bond and the electron-withdrawing nature of the substituent R can be used in an additive manner for 1-monosubstituted X-ureas, that is, for R^3^ = H, to improve the hydrogen-bond donor strength of the X-urea analog. However, for 1,3-disubstituted X-ureas (R^1^ and R^3^ ≠ H), we establish a steric mechanism that can eliminate the positive additivity of the two tuning factors. This leads in some complexes to an enhanced hydrogen-bond interaction for the O-ureas compared to the S- and Se-urea analogs, which is unexpected because of the lower experimental NH acidity of the former. Finally, we provide a proof of principle to overcome this steric limitation by introducing a predistorted urea-type hydrogen-bond donor. Given the broad application of urea derivatives in the field of supramolecular chemistry (*vide supra*), our insights into tuning urea hydrogen-bond donor strengths offer valuable design guidelines for a wide range of supramolecular systems, including but not limited to, hydrogen-bond donor catalysts, anion receptors, and hydrogen-bonded polymers.

## Results and discussion

### 1-Monosubstituted X-urea hydrogen-bond donors

**Fig. 2:**
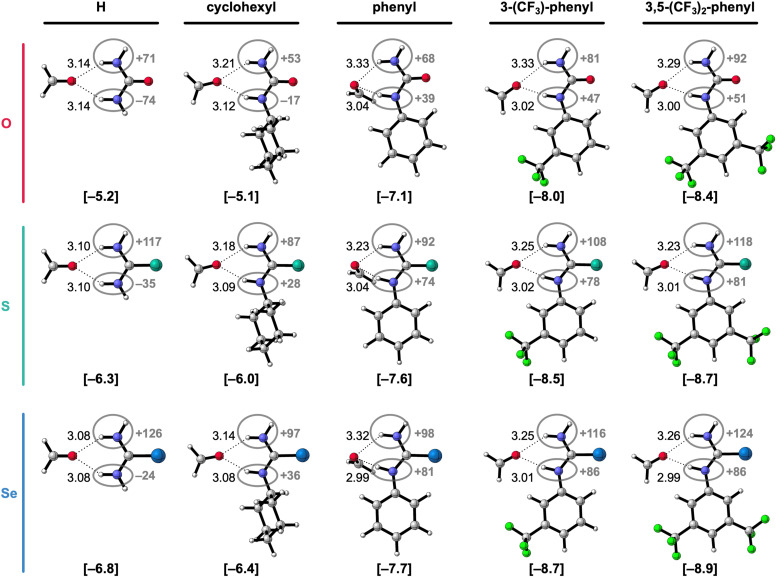
Equilibrium hydrogen-bonded complexes of formaldehyde with different 1-monosubstituted X-urea derivatives (X = O, S, or Se).^16,17^ The equilibrium hydrogen-bond (O⋯(H)N) distances (in Å) are indicated and the hydrogen-bond energies Δ*E* (in kcal mol^−1^) are shown below the structures between square brackets. The total Voronoi deformation density (VDD) atomic charge *Q*_NH(2)_ of the NH and NH_2_ group (in milli-electrons) of the isolated X-urea in the geometry of the hydrogen-bonded complex is highlighted in grey. Color code of the ball-and-stick structures: H = white; C = grey; N = dark blue; O = pink; F = green; S = turquoise; Se = light blue.

To systematically study the effect of simultaneously changing the chalcogen X in the C=X bond and the introduction of substituents at the amino groups on the hydrogen-bond donor ability of X-ureas, we started with adding only one substituent, *i.e.*, R^1^ = H, cyclohexyl, phenyl, 3-(CF_3_)-phenyl, or 3,5-(CF_3_)_2_-phenyl, while R^3^ = H (Fig. 1). The hydrogen-bonded complexes of these 1-monosubstituted X-urea derivatives with formaldehyde are presented in Fig. 2 alongside the equilibrium hydrogen-bond energies Δ*E* and distances, and the charge of the NH_(2)_ groups [this involves Voronoi deformation density (VDD)^15^ charges; see ESI,‡ Method S3 for details about this method]. We chose formaldehyde as hydrogen-bond acceptor molecule to isolate the effect of tuning the urea hydrogen-bond donor strengths in the absence of secondary (non-hydrogen bond) interactions with the steric bulk of other carbonyl compounds.

Fig. 2 shows that the hydrogen-bond energy Δ*E* (bold values in square brackets) becomes more stabilizing and the amino groups more positively charged (highlighted in grey) upon (i) changing X from O to S to Se, and (ii) upon introducing more electron-withdrawing substituents. While there is almost no effect on the hydrogen-bond energy going from R^1^ = H to R^1^ = cyclohexyl, the hydrogen-bond interaction is significantly strengthened for the electron-withdrawing aryl substituents.^16,17^ The phenyl group is known to withdraw electronic density from the NH group through the π-electronic system (see Fig. 2),^15*a*,*c*^ and can additionally improve the N–H hydrogen-bond donor strength through π-resonance assistance (*i.e.*, polarization) which makes the NH groups more *δ*^+^.^18^ Introducing the electron-withdrawing CF_3_ substituents on the phenyl group further enhances the positive charge on the NH groups (Fig. 2) and, consequently, the hydrogen-bond interaction.

To understand the different components that determine the relative stabilities of the formaldehyde⋯X–urea complexes in Fig. 2, Δ*E* was partitioned according to the activation strain model (ASM)^19^ of reactivity and bonding into a strain (Δ*E*_strain_) and interaction energy (Δ*E*_int_) component (eqn (1)).

1 Δ*E* = Δ*E*_strain_ + Δ*E*_int_

In this decomposition, Δ*E*_strain_ is the energy required to deform the X-urea and formaldehyde molecules in their separately optimized equilibrium geometries to the geometry they acquire in the hydrogen-bonded (equilibrium) complex. Δ*E*_int_ accounts for the stabilizing interaction between the two deformed molecules. The results of this analysis are presented in Table 1.

**Table 1:** Hydrogen-bond energies Δ*E* (in kcal mol^−1^) of the formaldehyde-1-monosubstituted X-urea (X = O, S, or Se) complexes, decomposed in terms of the strain energy Δ*E*_strain_ associated with distorting the molecules and the interaction energy Δ*E*_int_ between the distorted molecules: Δ*E* = Δ*E*_strain_ + Δ*E*_int_^a^^b^

R^1^	R^3^	X	Δ*E*	Δ*E*_strain_	Δ*E*_int_
H	H	O	−5.2	0.4	−5.6
		S	−6.3	0.5	−6.8
		Se	−6.8	0.3	−7.1
Cyclohexyl	H	O	−5.1	0.3	−5.4
		S	−6.0	0.3	−6.3
		Se	−6.4	0.2	−6.6
Ph	H	O	−7.1	0.3	−7.5
		S	−7.6	0.4	−8.0
		Se	−7.7	0.4	−8.2
3-(CF_3_)C_6_H_4_	H	O	−8.0	0.3	−8.3
		S	−8.5	0.5	−9.0
		Se	−8.7	0.5	−9.2
3,5-(CF_3_)_2_C_6_H_3_	H	O	−8.4	0.4	−8.7
		S	−8.7	0.7	−9.4
		Se	−8.9	0.7	−9.6

a All computed at ZORA-BLYP-D3(BJ)/TZ2P in *C*_1_ symmetry.

b See Fig. 2 for the corresponding structures.

Fig. 2 and Table 1 show that for a given chalcogen X, the hydrogen-bond interaction (Δ*E*) strengthens for the increasingly electron-withdrawing substituents R^1^, that is, from cyclohexyl to phenyl to 3-(CF_3_)-phenyl to 3,5-(CF_3_)_2_-phenyl. The decomposition in Table 1 shows that the strain energy Δ*E*_strain_ is small but increases (*i.e.*, is more destabilizing) for the effectively larger, more electron-withdrawing substituents. Thus, the strain does not dictate the stabilizing trend in Δ*E* for the more electron-withdrawing substituents. It is, in fact, the interaction energy Δ*E*_int_ that causes the stabilization of Δ*E* along this trend. The stabilization of Δ*E*_int_, and therefore Δ*E*, for the more electron-withdrawing substituents, can be understood from the increase of the positive charge on the X-urea NH_(2)_ groups (Fig. 2) that give rise to more stabilizing electrostatic and orbital interactions in the hydrogen-bonded complex with formaldehyde (see ESI,‡ Data S1: Table S1). Note that going from R^1^ = H to R^1^ = cyclohexyl, Δ*E*_int_, and thus Δ*E*, changes little, but the hydrogen-bond interaction becomes slightly weaker due to more steric Pauli repulsion associated with the steric bulk of the cyclohexyl substituent (see ESI,‡ Data S1: Table S1).

Besides upon introducing more electron-withdrawing substituents, the formaldehyde⋯X-urea hydrogen-bond energy Δ*E* also becomes more stabilizing by changing X from O to S to Se for each type of 1-monosubstituted X-urea derivatives (see Fig. 2 and Table 1). The reason for this is that thio- and selenoureas have more positive NH_(2)_ groups compared to ureas as is displayed in Fig. 2, and demonstrated and explained by us in our previous work.^7,9^ The larger steric size of the S and Se atoms pushes the C=X bond to a longer equilibrium distance at which the 
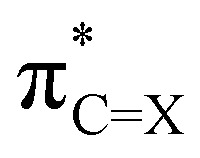
 LUMO drops in energy and becomes a better electron acceptor.^7^ This makes the C=S and C=Se groups effectively more electronegative than the C=O group in the sense that they can accommodate more charge of the lone pairs on the NH groups which become more positively charged (Fig. 2). The more positively charged NH_(2)_ groups cause a more stabilizing hydrogen-bond interaction Δ*E*_int_ for the S- and Se-urea analogs (Table 1) due to more stabilizing electrostatic and orbital interactions (see ESI,‡ Data S1: Table S1). The more stabilizing orbital interactions are also the result of the more positive NH groups, which cause an energetic lowering of the virtual 
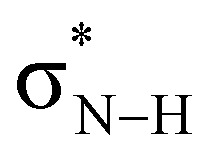
 orbitals involved in the covalent component of the hydrogen-bond interaction with formaldehyde's filled lone-pair orbitals (*i.e.*, the 
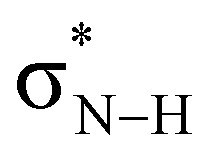
 orbitals become better electron acceptor orbitals).^7^

Note that the stabilizing trend of Δ*E* from X = O to S to Se for the 1-monosubstituted X-ureas is not dictated by the strain (Table 1). Δ*E*_strain_ is small and roughly constant for the different X-urea analogs for R^1^ = H and cyclohexyl. However, for the aryl substituents (R^1^ = Ph, 3-(CF_3_)-phenyl, and 3,5-(CF_3_)_2_-phenyl), the strain is slightly higher for X = S and Se than for X = O. This can be understood from the geometries of the free X-aryl-ureas and their deformation upon hydrogen bonding to formaldehyde. The O-aryl-urea monomers are nearly planar in their own equilibrium geometry which optimizes the π-orbital overlap between the urea core and the aryl substituent. At variance, the free S- and Se-aryl-ureas adopt a conformation in which the phenyl ring is rotated with respect to the urea core (see ESI,‡ Data S2: Table S3). We have shown before that this staggered conformation of the phenyl ring with respect to the amide group is caused by the stronger steric repulsion between the aromatic ring and the larger chalcogen atoms.^8^ However, upon forming the bifurcated hydrogen bonds with formaldehyde, the aryl-substituted X-ureas partially planarize whereby the aryl group rotates into the plane of the C=X bond (ESI,‡ Data S2: Table S3). This geometrical deformation (of which the origin is investigated in the next section) is associated with a destabilizing strain energy Δ*E*_strain_. Due to the larger chalcogen atom size, Δ*E*_strain_ is higher for X = S and Se than for X = O. This is attributed to two factors: (i) the degree of planarization upon hydrogen bonding is higher for the aromatic S- and Se-ureas because they initially adopt a more staggered conformation in the free X-urea compared to the O-ureas (*vide supra*), and (ii) the planarization is less favorable for a larger chalcogen atom X, due to greater steric repulsion, as we have shown in our previous work.^8^

### 1,3-Disubstituted X-urea hydrogen-bond donors

**Fig. 3:**
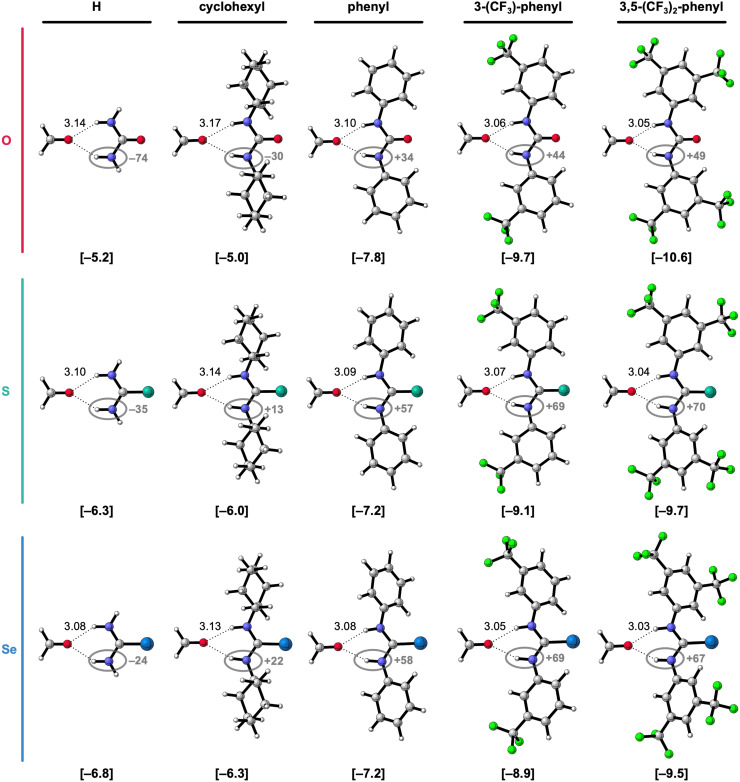
Equilibrium hydrogen-bonded complexes of formaldehyde with 1,3-disubstituted X-urea derivatives (X = O, S, or Se; R^1^ = R^3^).^17^ The equilibrium hydrogen-bond (O⋯(H)N) distances (in Å) are indicated and the hydrogen-bond energies Δ*E* (in kcal mol^−1^) are shown below the structures between square brackets. The total Voronoi deformation density (VDD) atomic charge *Q*_NH_ of the NH groups (in milli-electrons) of the isolated X-urea in the geometry of the hydrogen-bonded complex is highlighted in grey. Color code of the ball-and-stick structures: H = white; C = grey; N = dark blue; O = pink; F = green; S = turquoise; Se = light blue.

In the next step of our investigation, we study the effect of introducing a second substituent to the 1-monosubstituted X-urea analogs, thereby obtaining 1,3-disubstituted X-urea derivatives, which are more common in supramolecular systems than monosubstituted ones.^3–5^ The hydrogen-bonded complexes of these 1,3-disubstituted X-ureas and formaldehyde are presented in Fig. 3 alongside the equilibrium hydrogen-bond energies Δ*E* and distances, and the charges of the NH groups. The decomposition of Δ*E* through the ASM analysis of these complexes is presented in Table 2 (see ESI,‡ Method S2 for details about this method). Note that we only investigate the hydrogen-bond donor ability of the 1,3-disubstituted urea derivatives in the anti–anti conformation, that is, the conformation that can form the two bifurcated hydrogen bonds with formaldehyde and is the most relevant for supramolecular chemistry. Although several studies revealed that diphenylthioureas are more likely than diphenylureas to adopt other conformations (syn–anti or syn–syn),^20*a*,*b*^ it is shown that the increase of the polarity/Lewis basicity of the solvent or the introduction of CF_3_ groups on the phenyl ring switches the equilibrium towards the anti–anti conformer.^20^

**Table 2:** Hydrogen-bond energies Δ*E* (in kcal mol^−1^) of the formaldehyde-1,3-disubstituted X-urea (X = O, S, or Se) complexes, decomposed in terms of the strain energy Δ*E*_strain_ associated with distorting the molecules and the interaction energy Δ*E*_int_ between the distorted molecules: Δ*E* = Δ*E*_strain_ + Δ*E*_int_^a^^,^^b^

R^1^	R^3^	X	Δ*E*	Δ*E*_strain_	Δ*E*_int_
H	H	O	−5.2	0.4	−5.6
		S	−6.3	0.5	−6.8
		Se	−6.8	0.3	−7.1
Cyclohexyl	Cyclohexyl	O	−5.0	0.3	−5.3
		S	−6.0	0.1	−6.1
		Se	−6.3	0.1	−6.4
Ph	Ph	O	−7.8	0.1	−7.9
		S	−7.2	0.6	−7.8
		Se	−7.2	0.5	−7.7
3-(CF_3_)C_6_H_4_	3-(CF_3_)C_6_H_4_	O	−9.7	0.2	−9.9
		S	−9.1	1.3	−10.4
		Se	−8.9	0.8	−9.7
3,5-(CF_3_)_2_C_6_H_3_	3,5-(CF_3_)_2_C_6_H_3_	O	−10.6	0.2	−10.8
		S	−9.7	1.1	−10.8
		Se	−9.5	0.7	−10.2

a All computed at ZORA-BLYP-D3(BJ)/TZ2P in *C*_1_ symmetry.

b See Fig. 3 for the corresponding structures.

Fig. 3 reveals that also for the 1,3-disubstituted X-ureas, for a given chalcogen X, the hydrogen-bond interaction Δ*E* (bold values in square brackets) strengthens upon introducing the more electron-withdrawing substituents, that is, from R^1^,R^3^ = cyclohexyl to phenyl to 3-(CF_3_)-phenyl to 3,5-(CF_3_)_2_-phenyl, because Δ*E*_int_ becomes more stabilizing along this trend (see Table 2).^17^ This is again caused by the increase of the positive charge on the X-urea NH groups along this trend (highlighted in grey Fig. 3), which causes more stabilizing electrostatic and orbital interactions in the hydrogen-bonded complex with formaldehyde (see ESI,‡ Data S1: Table S2 and Fig. S1). Note again that going from R^1^,R^3^ = H to cyclohexyl only minorly influences the hydrogen-bond strength Δ*E* (Table 2). However, the introduction of the steric bulk by the cyclohexyl substituents, associated with an increase in steric Pauli repulsion, leads to a slight weakening of the intermolecular interaction (see ESI,‡ Data S1: Table S2).

Interestingly, Fig. 3 reveals that the hydrogen-bond interaction Δ*E* becomes only more stabilizing from X = O to S to Se for R^1^,R^3^ = H or cyclohexyl. For R^1^ and R^3^ being two aryl substituents, Δ*E* is the most stabilizing for X = O and then decreases for X = S and Se. This result is striking, as the heavier urea analogs are experimentally found more acidic,^6^ and therefore considered stronger hydrogen-bond donors,^7^ as is also expected from the more positively charged NH groups (Fig. 3) and as confirmed by us for the monosubstituted X-ureas (*vide supra*). While Fig. 3 involves symmetrically 1,3-disubstituted X-ureas, we also investigated two asymmetrically 1,3-disubstituted systems where we observed the same effect (see ESI,‡ Data S3). While for the formaldehyde-X-urea complexes with R^1^ = 3,5-(CF_3_)_2_-phenyl and R^3^ = cyclohexyl Δ*E* becomes more stabilizing from X = O to S to Se, for the complexes with R^1^ = 3,5-(CF_3_)_2_-phenyl and R^3^ = phenyl, that is for two aryl substituents, the hydrogen-bond interaction weakens for the larger chalcogens. Thus, our findings reveal that all 1,3-diaryl O-ureas form stronger hydrogen bonds to formaldehyde than the S- and Se-urea analogs (*i.e.*, with identical substituents). This result coincides with the experimental findings of Caillol, Andrioletti, and co-workers, who established that all of the investigated 1,3-diaryl substituted ureas are more active hydrogen-bond donor catalysts than their thiourea analogs in catalyzing carbonate ring-opening reactions, despite the experimentally determined higher acidity of the thioureas.^6*b*^ So, the computed hydrogen-bond energies Δ*E* of the hydrogen-bonded complexes seem to correlate better with the catalytic activity of the X-urea derivatives than experimental p*K*_a_ values. Although ground-state properties, like the hydrogen-bond donor strength of the X-urea derivatives, can only qualitatively correlate to transition-state properties that are crucial in catalysis, the performance of urea-based catalysts in hydrogen-bond activated reactions can often be estimated from the strength of the catalyst–substrate interaction.^21,22^

To get an understanding of what causes the breakdown of the additivity of the two tuning factors for the 1,3-diaryl X-ureas, we decomposed again the hydrogen-bond energy Δ*E* in terms of the strain energy Δ*E*_strain_ associated with distorting the molecules and the interaction energy Δ*E*_int_ between the deformed molecules (see eqn (1)). When looking at the results of this decomposition in Table 2, we observe that for X = S and Se, the 1,3-diaryl X-ureas have a weaker hydrogen-bond interaction Δ*E* because they encounter a more destabilizing Δ*E*_strain_, and in the case of X = Se, also a considerably less stabilizing Δ*E*_int_, compared to X = O.

The more destabilizing Δ*E*_strain_ for the systems with X = S and Se can be understood from the geometries of the free 1,3-diaryl X-ureas and their deformation upon hydrogen bonding to formaldehyde. As for the monosubstituted aryl ureas, the free 1,3-diaryl O-ureas are close to planar in which the π-orbital overlap between the urea core and the aryl substituents is maximized, while the 1,3-diaryl S- and Se-ureas adopt a conformation wherein the phenyl rings are rotated with respect to the urea core (see ESI,‡ Data S2: Table S4). We have shown before that this staggered conformation is caused by the stronger steric repulsion between the phenyl ring and the larger chalcogen atoms in the C=X bond.^8^ However, upon forming the bifurcated hydrogen bonds with formaldehyde, we observe that the diaryl X-ureas partially planarize as the phenyl rings rotate into the plane of the C=X bond (ESI,‡ Data S2: Table S4), giving rise to a destabilizing Δ*E*_strain_. Δ*E*_strain_ increases for the larger chalcogen atoms because they push the phenyl rings of the free X-urea more out of the plane and then also make it more difficult to rotate back. The latter occurs because the planarization strengthens the hydrogen-bond interaction (*vide infra*). Thus, the planarization of 1,3-diaryl X-ureas upon hydrogen bonding is more pronounced and harder for the larger chalcogen atoms, giving rise to a more destabilizing deformation strain. These findings are in line with the study by Ho *et al.* that showed that 1,3-diphenylthiourea has a lower chloride anion binding affinity than 1,3-diphenylurea because the thiourea analog needs to planarize from a twisted to a more sterically congested planar configuration in order to facilitate anion binding, which comes with a cost of increased steric interactions compared to the urea analog.^23^

**Fig. 4:**
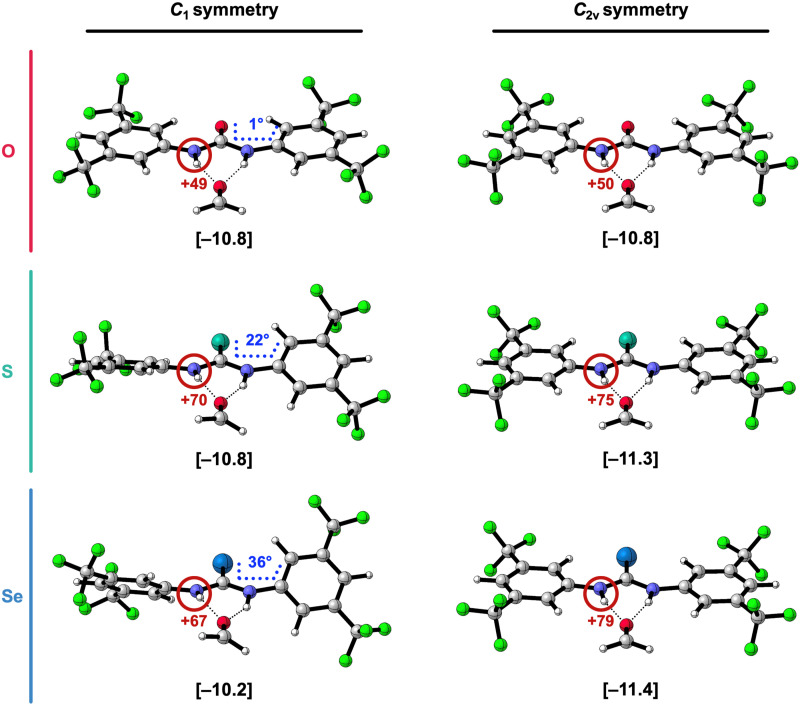
Side-view of the hydrogen-bonded complexes of formaldehyde and [1,3-(3,5-(CF_3_)_2_-phenyl)]X-ureas (X = O, S, or Se) in their equilibrium geometry (*C*_1_ symmetry) and in the geometry of the *C*_2v_ symmetry enforced optimization. The hydrogen-bond interaction energies Δ*E*_int_ (in kcal mol^−1^) are shown below the structures between square brackets. The total Voronoi deformation density (VDD) atomic charge *Q*_NH_ of the NH group (in milli-electrons) of the isolated X-urea in the geometry of the hydrogen-bonded complex is highlighted in red and the XC-C_Ar_C_Ar_ dihedral angle (in degrees) is highlighted in blue (note that this angle is 0° for the *C*_2v_ symmetric systems). Color code of the ball-and-stick structures: H = white; C = grey; N = dark blue; O = pink; F = green; S = turquoise; Se = light blue.

Due to the higher energetic cost of planarization for the larger chalcogens S and Se, the 1,3-diaryl thio- and selenoureas cannot be as flat as for X = O (see Fig. 4 for an example and ESI,‡ Data S2: Table S4 for the other complexes). In addition, the presence of a second aryl substituent further diminishes the degree of possible planarization of the 1,3-diaryl S- and Se-ureas compared to the 1-aryl analogs (ESI,‡ Data S2: Table S3 *vs.* Table S4). The inability of planarization of the 1,3-diaryl thio- and selenoureas weakens the hydrogen-bond interaction Δ*E*_int_ with formaldehyde relative to the oxygen analogs, despite the more positively charged NH groups (highlighted in grey Fig. 3) and higher experimental acidity^6^ of the heavier chalcogen X-urea analogs. This is likely attributed to two effects: for X = S and Se (i) the non-planar NH groups are not optimally aligned towards the formaldehyde hydrogen-bond acceptor; (ii) because the π-electronic system cannot optimally overlap, the nitrogen lone pairs can donate less electron density towards the C=X bond and the aryl substituents so that the NH groups become less positively charged (*vide infra*).^7,15*a*,*c*^ Thus, the poorly aligned and the relatively less positive NH groups make that the higher steric Pauli repulsion for the larger chalcogen analogs dominates in the hydrogen-bond interaction over the slightly more stabilizing electrostatic and orbital interactions compared to the oxygen analogs, which disrupts the usual stabilization of Δ*E*_int_ from X = O to S to Se in the 1,3-diaryl urea-formaldehyde complexes (see ESI,‡ Data S1: Table S2).

To confirm that the 1,3-diaryl thio- and selenoureas are stronger hydrogen-bond donors when they become planar, we reoptimized the hydrogen-bonded complexes of formaldehyde with the by 3,5-(CF_3_)_2_-phenyl 1,3-disubstituted X-ureas while enforcing *C*_2v_ symmetry, to obtain planar structures (Fig. 4). As presented in Fig. 4 and Table 3, the stabilizing trend of the hydrogen-bond interaction energy Δ*E*_int_ from X = O to S to Se is restored in these planar complexes. Note that for X = O, there is no significant effect because the *C*_1_ complex is already close to planar. Thus, the switch in trend going from the equilibrium (*C*_1_) complexes to the *C*_2v_ complexes, is caused by Δ*E*_int_ getting more stabilizing for X = S and Se in the planar systems.

**Table 3:** Decomposition of the hydrogen-bond interaction energy Δ*E*_int_ (in kcal mol^−1^) of the equilibrium (*C*_1_) and planar (*C*_2v_) formaldehyde-1,3-[3,5-(CF_3_)_2_-phenyl]X-urea (X = O, S, or Se) complexes^a^^b^^c^

R^1^	R^3^	X	Δ*E*_int_	Δ*V*_elstat_	Δ*E*_Pauli_	Δ*E*_oi_	Δ*E*_disp_
***C***_**1**_ **symmetric**							
3,5-(CF_3_)_2_C_6_H_3_	3,5-(CF_3_)_2_C_6_H_3_	O	−10.8	−12.2	10.0	−5.9	−2.7
		S	−10.8	−12.4	10.6	−6.1	−2.9
		Se	−10.2	−12.1	10.4	−5.9	−2.7
***C***_**2v**_ **symmetric**							
3,5-(CF_3_)_2_C_6_H_3_	3,5-(CF_3_)_2_C_6_H_3_	O	−10.8	−12.2	10.0	−5.9	−2.7
		S	−11.3	−12.8	11.0	−6.4	−3.1
		Se	−11.4	−13.0	11.3	−6.5	−3.2

a All computed at ZORA-BLYP-D3(BJ)/TZ2P.

b Δ*E*_int_ = Δ*V*_elstat_ + Δ*E*_Pauli_ + Δ*E*_oi_ + Δ*E*_disp_.

c See Fig. 4 for the corresponding structures.

To understand what causes this stabilization, Δ*E*_int_ was partitioned into four physically meaningful terms using a quantitative energy decomposition analysis (EDA)^24^: (i) the classical electrostatic interaction (Δ*V*_elstat_), (ii) the steric Pauli repulsion (Δ*E*_Pauli_) arising from the repulsion between overlapping closed-shell orbitals on the interacting molecules, (iii) the orbital interaction (Δ*E*_oi_) which accounts for charge transfer (*i.e.*, covalency) in the σ-electronic system and polarization of the π-electronic system, and (iv) the dispersion energy (Δ*E*_disp_) (see eqn (2); see ESI,‡ Method S2 for a theoretical overview of this method).

2 Δ*E*_int_ = Δ*V*_elstat_ + Δ*E*_Pauli_ + Δ*E*_oi_ + Δ*E*_disp_

Table 3 reveals that in the *C*_2v_ symmetric systems with X = S and Se, Δ*E*_int_ becomes indeed more stabilizing compared to the equilibrium (*C*_1_) structures mainly because the electrostatic Δ*V*_elstat_ and orbital interactions Δ*E*_oi_ get more stabilizing. This can be understood from the observation that the NH groups are better aligned and become more positively charged when the aromatic substituents are coplanar with the urea core in the *C*_2v_ symmetric complexes (Fig. 4). When the phenyl rings and the C=X bond are coplanar with the NH groups, they have a more pronounced electron-withdrawing effect because there is more orbital overlap in the π-system, thus the C=X group and the aryl groups can accept more π-electronic density from the nitrogen lone pairs of the NH groups.^7,15*a*,*c*^

### A predistorted X-urea hydrogen-bond donor

The above-identified steric limitation of improving the hydrogen-bond donor strength of 1,3-diaryl X-ureas by introducing the larger chalcogens S or Se in the C=X bond is unfortunate because S- and Se-ureas have the potential to engage in stronger orbital interactions with a hydrogen-bond acceptor than O-ureas (*e.g.*, see Δ*E*_oi_ in Table 3 for the *C*_2v_ symmetric systems),^7^ which can be useful for their application in hydrogen-bond activated reactions.^21^ In this section, we demonstrate a proof of principle of a predistorted urea hydrogen-bond donor comprising the group 14 elements in the urea C=X bond where the steric limitation of using larger elements X is eliminated so that the hydrogen-bond donor strength of X-ureas bearing two aryl substituents can be enhanced upon varying X down a group. This suggestion is based on our previous work where we showed that the NH hydrogen-bond donor strength of amides can also be enhanced upon varying X in the amide C=X bonds down groups 14 and 15 of the periodic table.^9^ The tetravalency of the group 14 elements allows for fixation of the conformation of the aryl substituents, which enables preorganization of the substituents in the X-urea monomers (see Table 4).

**Table 4:** Hydrogen-bond energies Δ*E* (in kcal mol^−1^) of the formaldehyde-1,3-disubstituted X-urea (X = C, Si, or Ge) complexes, decomposed in terms of the strain energy Δ*E*_strain_ associated with distorting the fragments and the interaction energy Δ*E*_int_ between the distorted fragments: Δ*E* = Δ*E*_strain_ + Δ*E*_int_^a^^b^

	X	Δ*E*	Δ*E*_strain_	Δ*E*_int_
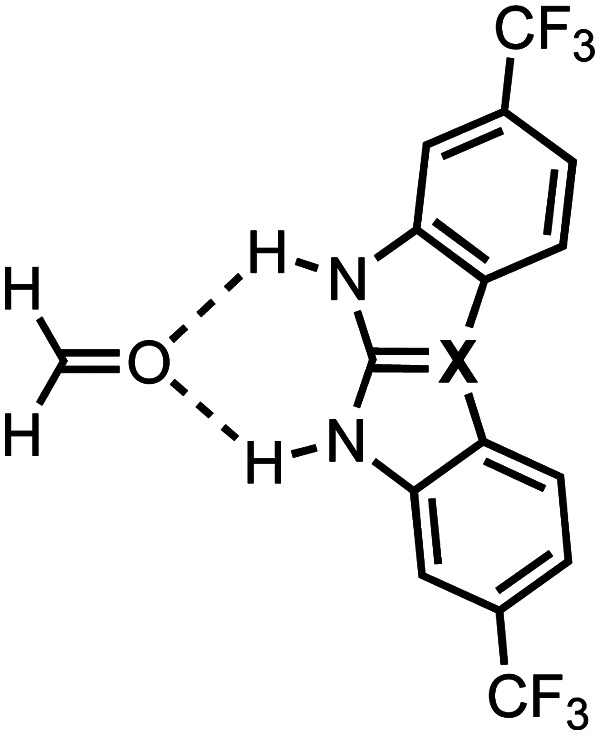	C	−7.7	0.3	−8.0
	Si	−8.4	0.3	−8.7
	Ge	−8.7	0.2	−8.9

a All computed at ZORA-BLYP-D3(BJ)/TZ2P in *C*_1_ symmetry.

b See ESI Data S4: Fig. S3 for the corresponding structures.

The hydrogen-bond energies Δ*E* of these group 14 urea derivatives and formaldehyde are presented in Table 4 and show that Δ*E* becomes more stabilizing down group 14 from X = C to Si to Ge (see ESI,‡ Data S4: Fig. S3 for the corresponding structures). Note that the fixation of the aromatic rings to the tetravalent group 14 elements equalizes the strain Δ*E*_strain_ for all three group 14 elements X, thus the strain no longer dictates the trend of the hydrogen-bond donor strengths (see Table 4). In these predistorted systems, it is again Δ*E*_int_ that causes the hydrogen-bond interaction to strengthen from X = C to Si to Ge (Table 4). Δ*E*_int_ is more stabilizing from X = C to Si to Ge due to more stabilizing electrostatic and orbital interactions between the X-urea and formaldehyde along this trend (see ESI,‡ Data S4: Table S7) as the Si- and Ge-urea analogs have more positive NH groups compared to the C-urea (see ESI,‡ Data S4: Fig. S3), as was also shown in our previous work.^9^

## Conclusions

The hydrogen-bond donor strength of ureas can, in general, be enhanced by increasing the size of the chalcogen X in the C=X bond from O to S to Se and by introducing more electron-withdrawing substituents because both effects increase the positive charge on the NH groups. However, for 1,3-diaryl X-ureas, there is a steric mechanism that disrupts the positive additivity of the two tuning factors, as appears from our relativistic, dispersion-corrected DFT analyses. This leads in these 1,3-diaryl X-urea derivatives to an enhanced hydrogen-bond donor strength for ureas (X = O) compared to the thiourea (X = S) and selenourea (X = Se) analogs (*i.e.*, with identical substituents), despite the experimentally observed lower NH acidity of the former.

The reason for the breakdown of the additivity of N-substituent and X-variation effects is that X-urea hydrogen-bond donors bearing aromatic substituents can form the strongest hydrogen-bond interactions only when they adopt a completely planar conformation. This makes the π-electronic system fully conjugated so that the π-electron withdrawing effect of the C=X bond and the aromatic substituents on the NH groups is the largest (thus making the NH group more positively charged), and it ensures that the NH groups are optimally aligned towards the hydrogen-bond acceptor. However, when having the larger chalcogens S and Se in the urea C=X bond, the rotation of the aromatic substituents into the plane of the urea core is hampered due to the higher steric repulsion of the phenyl rings with the larger chalcogen atoms. The resulting non-planar conformation weakens the hydrogen-bond donor strength of 1,3-diaryl X-ureas with X = S and Se compared to X = O in two ways: (i) hydrogen-bond formation involving the S- and Se-ureas goes with a higher deformation strain that occurs upon the associated conformational change toward (incomplete) planarization; and (ii) by not being fully planar, the π-electron withdrawing effect of the C=X group and the aromatic substituents on the NH groups is diminished due to a reduced π-overlap.

Finally, we provide a proof of principle to eliminate this steric limitation by introducing a predistorted type of X-urea hydrogen-bond donors comprising the tetravalent group 14 elements in the C=X bond. Our novel insights into tuning the hydrogen-bond donor strength of urea derivatives aid in providing design principles for supramolecular systems, including but not limited to, hydrogen-bond donor catalysts, anion receptors, and hydrogen-bonded polymers.

## Author contributions

CFG conceived the project. CN and ANvD carried out the quantum-chemical computations and bonding analyses. CN, CFG, and FMB drafted the manuscript. All authors discussed the results and reviewed the manuscript.

## Data availability

Additional computational results that support the findings in this work, full computational details, and the Cartesian coordinates and energies of the reported molecules and complexes have been uploaded as part of the ESI,‡ which cites additional ref. 25–27

## Conflicts of interest

The authors declare no conflict of interest.

## Supplementary Material

CP-027-D4CP04042B-s001

CP-027-D4CP04042B-s002
