# Supplementary material for: Urea hydrogen-bond donor strengths: bigger is not always better
Source: Phys Chem Chem Phys. 2024 Dec 5;27(8):4099–108. doi: 10.1039/d4cp04042b (PMC11632590; doi:10.1039/d4cp04042b)
Supplement: CP-027-D4CP04042B-s001 [file CP-027-D4CP04042B-s001.pdf]

Supplementary Information (SI) for Physical Chemistry Chemical Physics.
This journal is © the Owner Societies 2024

Electronic Supplementary Material (ESI)

for

Urea Hydrogen-Bond Donor Strengths: Bigger is not Always Better

Celine Nieuwland,[a] Angelina N. van Dam,[a]
F. Matthias Bickelhaupt,[a,b,c] and Célia Fonseca Guerra*[a]

[a] Department of Chemistry and Pharmaceutical Sciences, Amsterdam Institute of Molecular and Life Sciences (AIMMS), Vrije Universiteit Amsterdam, De Boelelaan 1108, 1081 HZ Amsterdam, The Netherlands
E-mail: c.fonsecaguerra@vu.nl | Homepage: https://www.theochem.nl/

[b] Institute for Molecules and Materials, Radboud University, Heyendaalseweg 135, 6525 AJ Nijmegen, The Netherlands

[c] Department of Chemical Sciences, University of Johannesburg, Auckland Park, Johannesburg 2006, South Africa

Table of Contents

Method S1. Computational details **2**
Method S2. Hydrogen-bond and interaction energy analyses **3**
Method S3. Voronoi deformation density (VDD) charges **5**
Data S1. Formaldehyde–X-urea complexes (X = O, S, Se) **6**
Data S2. Geometries of the (di)aryl substituted X-urea derivatives (X = O, S, Se) **8**
Data S3. Asymmetric formaldehyde–1,3-disubstituted X-urea complexes (X = O, S, Se) **9**
Data S4. Formaldehyde–X-urea complexes (X = C, Si, Ge) **11**
Data S5. 1,3-bis[3-(CF_3)phenyl]urea vs. 1,3-bis[4-(CF_3)phenyl]urea **13**
Data S6. Cartesian coordinates and energies of optimized monomers **14**
Data S7. Cartesian coordinates and energies of optimized hydrogen-bonded complexes . **39**

Method S1. Computational details

All calculations were performed using the Amsterdam Density Functional (ADF) program as implemented in the Amsterdam Modeling Suite (AMS, version 2023.1).[S1] The dispersion-corrected relativistic density functional theory (DFT-D) calculations were performed at the ZORA-BLYP-D3(BJ)[S2]/TZ2P level of theory, which has been proven to be adequate for studying these types of hydrogen-bonded systems.[S3] The TZ2P basis set[S1a,b] consists of an uncontracted set of Slater-type orbitals (STOs) of triple-ζ quality for all atoms, augmented with two sets of polarization functions. The ZlmFit[S4a] density fitting scheme with the Becke[S4b,c] integration grid, at "Excellent" quality, was used for geometry optimizations and subsequent (bonding) analyses. All optimized geometries have been checked to be energy minima by performing a vibrational frequency analysis (*i.e.,* no imaginary frequencies for minima, see Data S6 and S7).[S5] The ball-and-stick structures of the optimized structures in this work were created using the CYLview20[S6] visualization software.

[S1] (a) G. te Velde, F. M. Bickelhaupt, E. J. Baerends, C. Fonseca Guerra, S. J. A. van Gisbergen and J. G. Snijders, T. Ziegler, *J. Comput. Chem.*, 2001, **22**, 931; (b) E. van Lenthe, E. J. Baerends, *J. Comput. Chem.,* 2003, **24**, 1142; (c) C. Fonseca Guerra, J. G. Snijders, G. te Velde and E. J. Baerends, *Theor. Chem. Acc.*, 1998, **99**, 391; (d) ADF 2023.1, SCM, Theoretical Chemistry, Vrije Universiteit Amsterdam, Amsterdam, The Netherlands, www.scm.com; (e) AMS 2023.1, SCM, Theoretical Chemistry, Vrije Universiteit Amsterdam, Amsterdam, The Netherlands, www.scm.com.

[S2] (a) E. van Lenthe, A. Ehlers and E. J. Baerends, *J. Chem. Phys.,* 1999, **110**, 8943; (b) A. D. Becke, *Phys. Rev. A,* 1988, **38**, 3098; (c) C. Lee, W. Yang and R. G. Parr, *Phys. Rev. B,* 1988, **37**, 785; (d) Q. Wu and W. Yang, *J. Chem. Phys*., 2002, **116**, 515; (e) S. Grimme, *J. Comput. Chem.,* 2004, **25**, 1463; (f) S. Grimme, *J. Comput. Chem.,* 2006, **27**, 1787; (g) S. Grimme, J. Antony, S. Ehrlich and H. Krieg, *J. Chem. Phys.,* 2010, **132**, 154104; (h) S. Grimme, S. Ehrlich and L. Goerigk, *J. Comput. Chem.,* 2011, **32**, 1456; (i) A. D. Becke and E. R. Johnson, *J. Chem. Phys.,* 2005, **122**, 154101; (j) E. R. Johnson and A. D. Becke, *J. Chem. Phys.,* 2006, **124**, 024101.

[S3] C. Nieuwland and C. Fonseca Guerra, *Chem. Eur. J.,* 2022, **28**, e202200755.

[S4] (a) M. Franchini, P. H. T. Philipsen, E. van Lenthe and L. Visscher, *J. Chem. Theory Comput.,* 2014, **10**, 1994; (b) A. D. Becke, *J. Chem. Phys.,* 1998, **88**, 2547; (c) M. Franchini, P. H. T. Philipsen and L. Visscher, *J. Comput. Chem.,* 2013, **34**, 1819.

[S5] (a) S. K. Wolff, *Int. J. Quantum Chem.,* 2005, **104**, 645; (b) A. Bérces, R. M. Dickson, L. Fan, H. Jacobsen, D. P. Swerhone and T. Ziegler, *Comput. Phys. Commun.,* 1997, **100**, 247; (c) H. Jacobsen, A. Bérces, D. P. Swerhone and T. Ziegler, *Comput. Phys. Commun.,* 1997, **100**, 263.

[S6] C. Y. Legault, *CYLview20*, Université de Sherbrooke, Sherbrooke, Quebec, Canada, 2020, www.cylview.org.

Method S2. Hydrogen-bond and interaction energy analyses

In this work, the hydrogen-bond energy between the various urea hydrogen-bond donors (**X-urea**, with X = O, S, Se, C, Si, or Ge) and the hydrogen-bond acceptor formaldehyde (**F**) was computed. The hydrogen-bond energy (ΔE) is computed following Equation S1.

$$\Delta E = E(\mathbf{F}\cdots\mathbf{X\text{-}urea}) - E(\mathbf{F}) - E(\mathbf{X\text{-}urea}) \tag{S1}$$

In this equation, E(**F**···**X-urea**), E(**F**), and E(**X-urea**) correspond to the electronic energies E of the formaldehyde–X-urea hydrogen-bonded complex (**F**···**X-urea**), formaldehyde, and the X-urea, each in their optimized geometry, respectively.

To understand the different components that determine the relative stabilities of the **F**···**X-urea** complexes, ΔE was partitioned according to the activation strain model (ASM)[S7] of reactivity and bonding into a strain and interaction energy component (Equation S2).

$$\Delta E = \Delta E_{\text{strain}} + \Delta E_{\text{int}} \tag{S2}$$

In this decomposition, the strain energy (ΔE_{strain}) is the energy required to deform the separately optimized geometries of the X-urea and formaldehyde molecules, to the geometry they acquire in the hydrogen-bonded complex. The interaction energy (ΔE_{int}) accounts for the stabilizing interaction between the two prepared (*i.e.,* deformed) molecules.

The interaction energy ΔE_{int} can be further decomposed based on Kohn-Sham molecular orbital theory using a quantitative energy decomposition analysis (EDA).[S8] In the EDA, the total interaction energy (ΔE_{int}) is decomposed into components of electrostatic interaction (ΔV_{elstat}), Pauli repulsion (ΔE_{Pauli}), orbital interaction (ΔE_{oi}), and dispersion (ΔE_{disp}) (see Equation S3).

$$\Delta E_{\text{int}} = \Delta V_{\text{elstat}} + \Delta E_{\text{Pauli}} + \Delta E_{\text{oi}} + \Delta E_{\text{disp}} \tag{S3}$$

[S7] (a) P. Vermeeren, S. C. C. van der Lubbe, C. Fonseca Guerra, F. M. Bickelhaupt and T. A. Hamlin, *Nat. Protoc.,* 2020, **15**, 649; (b) P. Vermeeren, T. A. Hamlin and F. M. Bickelhaupt, *Chem. Commun.,* 2021, **57**, 5880.

[S8] (a) F. M. Bickelhaupt and E. J. Baerends, in *Reviews in Computational Chemistry*, eds. K. B. Lipkowitz and D. B. Boyd, Wiley-VCH, New York, Vol. **15**, 2000, pp. 1–86; (b) T. A. Hamlin, P. Vermeeren, C. Fonseca Guerra and F. M. Bickelhaupt, in *Complementary Bonding Analysis*, ed. S. Grabowsky, De Gruyter, Berlin, 2021, pp. 199–212.

Here, ΔV_{elstat} comprises the (usually attractive) classical electrostatic interactions between the unperturbed charge distributions of the prepared (*i.e.,* deformed) interacting molecules, that is, the X-urea and formaldehyde. ΔE_{Pauli} accounts for the destabilizing interactions between the interacting molecules resulting from overlapping closed-shell orbitals and accounts for any steric repulsion. The ΔE_{oi} term comprises charge transfer between the interacting molecules (*i.e.,* donor–acceptor interactions between occupied and unoccupied orbitals on the interacting molecules, including HOMO–LUMO interactions) and mutual polarization of the interacting molecules (*i.e.,* empty–occupied orbital mixing on one molecule due to the presence of the other molecule).

Method S3. Voronoi deformation density (VDD) charges

The Voronoi deformation density (VDD) charge analysis allows for the quantification of the flow of electronic charge as a direct consequence of chemical-bond formation.[S9] VDD atomic charges (Q) are computed by the spatial integration of the deformation density over the Voronoi cell of atom A, which is the space defined by the bond midplanes on and perpendicular to all bond axes between this atom A and its neighboring atoms (see Equation S4).

$$Q = -\int_{\text{Voronoi cell of A}} [\rho(\mathbf{r}) - \textstyle\sum_i \rho_i(\mathbf{r})]\, d\mathbf{r} \qquad \text{(S4)}$$

Herein, the deformation density $\Delta\rho(\mathbf{r}) = [\rho(\mathbf{r}) - \sum_i \rho_i(\mathbf{r})]$ is the density change going from a superposition of the original atomic densities at the positions of the molecule to the actual density of that molecule. This atomic or so-called *promolecular* density is defined as the sum of the (spherically averaged) ground-state atomic densities $\sum_i \rho_i(\mathbf{r})$. This is the fictitious state in which the charge density has not been affected by chemical bonding and in which all atoms have zero charge. Q in Equation S4 then represents the amount of charge that, due to chemical bonding, flows to a position closer to nucleus A ($Q < 0$) or to a position further away from nucleus A ($Q > 0$).

[S9] (a) C. Nieuwland, P. Vermeeren, F. M. Bickelhaupt and C. Fonseca Guerra, *J. Comput. Chem.,* 2023, **44**, 2108; (b) C. Fonseca Guerra, J. W. Handgraaf, E. J. Baerends and F. M. Bickelhaupt, *J. Comput. Chem.,* 2004, **25**, 189; (c) O. A. Stasyuk, H. Szatylowicz, T. M. Krygowski and C. Fonseca Guerra, *Phys. Chem. Chem. Phys.,* 2016, **18**, 11624.

Data S1. Formaldehyde–X-urea complexes (X = O, S, Se)

Table S1. Decomposition of the hydrogen-bond energy ΔE (in kcal mol^{-1}) of the formaldehyde–1-monosubstituted X-urea (X = O, S, or Se) complexes.[a,b,c]

R^1	R^3	X	ΔE	ΔE_{strain}	ΔE_{int}	ΔV_{elstat}	ΔE_{Pauli}	ΔE_{oi}	ΔE_{disp}
H	H	O	–5.2	0.4	–5.6	–7.0	5.6	–2.7	–1.6
		S	–6.3	0.5	–6.8	–8.4	6.9	–3.6	–1.7
		Se	–6.8	0.3	–7.1	–9.0	7.6	–3.9	–1.8
Cyclohexyl	H	O	–5.1	0.3	–5.4	–6.7	6.1	–2.8	–2.0
		S	–6.0	0.3	–6.3	–7.9	7.4	–3.5	–2.2
		Se	–6.4	0.2	–6.6	–8.5	7.9	–3.9	–2.2
Ph	H	O	–7.1	0.3	–7.5	–8.4	8.4	–3.6	–3.9
		S	–7.6	0.4	–8.0	–8.9	9.1	–3.9	–4.3
		Se	–7.7	0.4	–8.2	–9.7	10.1	–4.6	–3.9
3-(CF_3)C_6H_4	H	O	–8.0	0.3	–8.3	–9.5	8.7	–4.7	–2.9
		S	–8.5	0.5	–9.0	–10.4	9.7	–5.2	–3.1
		Se	–8.7	0.5	–9.2	–10.8	10.1	–5.4	–3.1
3,5-(CF_3)$_2$$C_6H_3$	H	O	–8.4	0.4	–8.7	–10.0	8.8	–4.9	–2.7
		S	–8.7	0.7	–9.4	–10.8	9.8	–5.4	–3.0
		Se	–8.9	0.7	–9.6	–11.1	10.3	–5.7	–3.1

[a] All computed at ZORA-BLYP-D3(BJ)/TZ2P in C_1 symmetry. [b] $\Delta E = \Delta E_{strain} + \Delta E_{int}$; $\Delta E_{int} = \Delta V_{elstat} + \Delta E_{Pauli} + \Delta E_{oi} + \Delta E_{disp}$ (see Method S2 for details). [c] See Figure 2 in the main text for the corresponding structures.

Table S2. Decomposition of the hydrogen-bond energy ΔE (in kcal mol^{-1}) of the symmetric formaldehyde–1,3-disubstituted X-urea (X = O, S, or Se) complexes.[a,b,c]

R^1	R^3	X	ΔE	ΔE_{strain}	ΔE_{int}	ΔV_{elstat}	ΔE_{Pauli}	ΔE_{oi}	ΔE_{disp}
H	H	O	–5.2	0.4	–5.6	–7.0	5.6	–2.7	–1.6
		S	–6.3	0.5	–6.8	–8.4	6.9	–3.6	–1.7
		Se	–6.8	0.3	–7.1	–9.0	7.6	–3.9	–1.8
Cyclohexyl	Cyclohexyl	O	–5.0	0.3	–5.3	–6.5	6.2	–2.8	–2.2
		S	–6.0	0.1	–6.1	–7.7	7.5	–3.5	–2.4
		Se	–6.3	0.1	–6.4	–8.2	8.1	–3.8	–2.5
Ph	Ph	O	–7.8	0.1	–7.9	–9.5	8.6	–4.4	–2.6
		S	–7.2	0.6	–7.8	–9.6	9.0	–4.6	–2.7
		Se	–7.2	0.5	–7.7	–9.7	9.2	–4.6	–2.6
3-(CF_3)C_6H_4	3-(CF_3)C_6H_4	O	–9.7	0.2	–9.9	–11.3	9.6	–5.4	–2.7
		S	–9.1	1.3	–10.4	–12.0	10.6	–5.9	–3.1
		Se	–8.9	0.8	–9.7	–11.6	10.3	–5.6	–2.8
3,5-(CF_3)$_2$$C_6H_3$	3,5-(CF_3)$_2$$C_6H_3$	O	–10.6	0.2	–10.8	–12.2	10.0	–5.9	–2.7
		S	–9.7	1.1	–10.8	–12.4	10.6	–6.1	–2.9
		Se	–9.5	0.7	–10.2	–12.1	10.4	–5.9	–2.7

[a] All computed at ZORA-BLYP-D3(BJ)/TZ2P in C_1 symmetry. [b] $\Delta E = \Delta E_{strain} + \Delta E_{int}$; $\Delta E_{int} = \Delta V_{elstat} + \Delta E_{Pauli} + \Delta E_{oi} + \Delta E_{disp}$ (see Method S2 for details). [c] See Figure 3 in the main text for the corresponding structures.

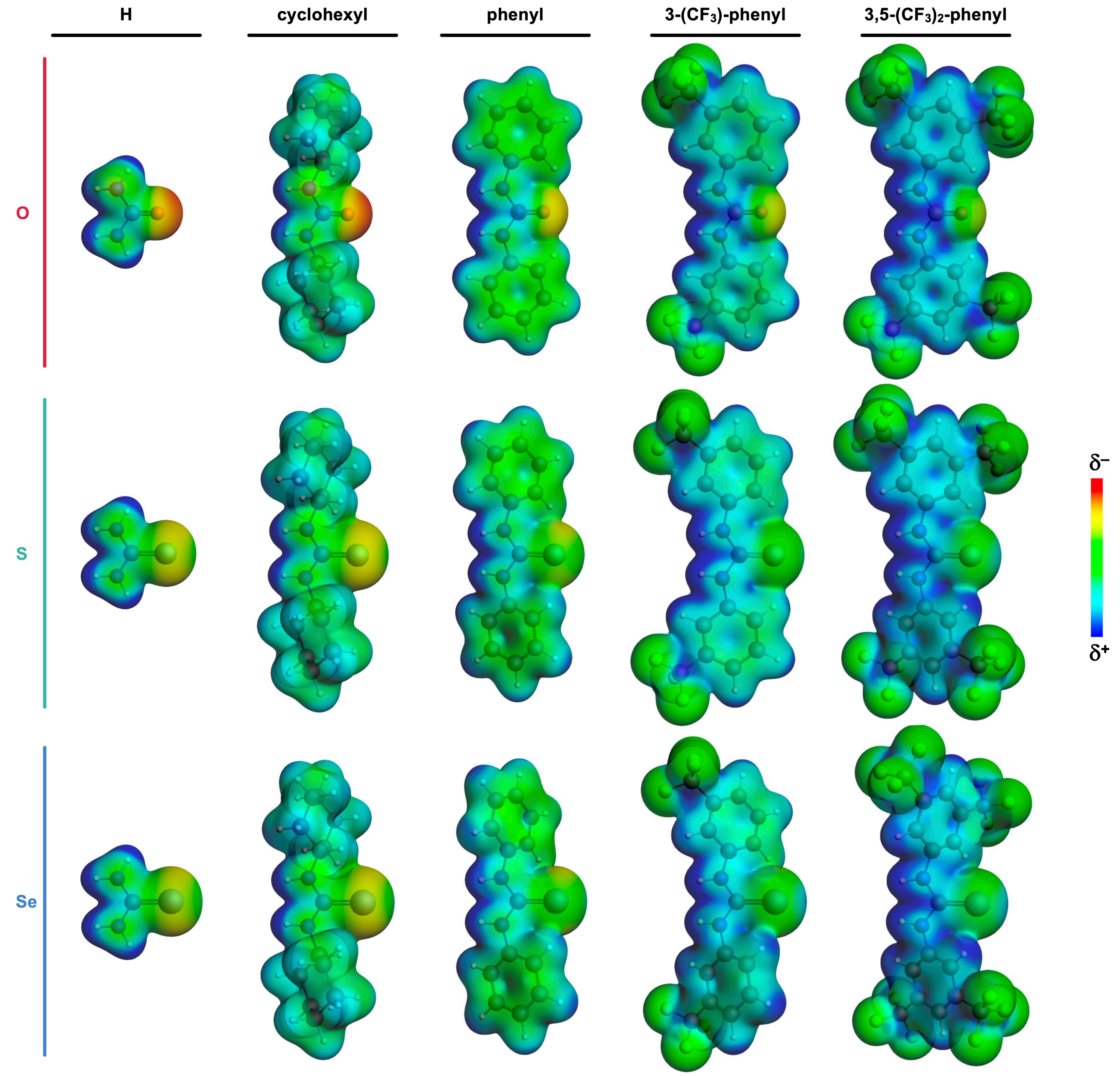

Figure S1. Molecular electrostatic potential surfaces (at 0.01 a.u.) from −0.1 (red) to +0.1 (blue) a.u. of the isolated 1,3-disubstituted X-urea derivatives (X = O, S, or Se; $R^1 = R^3$) in the geometry of the hydrogen-bonded complex with formaldehyde.

Data S2. Geometries of the (di)aryl substituted X-urea derivatives (X = O, S, Se)

Table S3. XC–$C_{Ar}C_{Ar}$ dihedral angle θ (in degrees; highlighted in blue below) of the 1-aryl monosubstituted X-urea derivatives (X = O, S, or Se) in the equilibrium geometry of the X-urea monomer ($\theta_{monomer}$) and that of the hydrogen-bonded complex with formaldehyde ($\theta_{complex}$).[a]

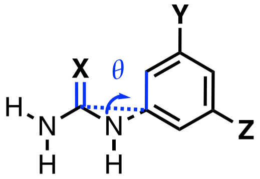

X	Y	Z	$\theta_{monomer}$	$\theta_{complex}$	$\Delta\theta_{monomer\rightarrow complex}$[b]
O	H	H	6.0	3.0	–3.0
S			25.6	1.8	–23.8
Se			30.8	12.1	–18.7
O	H	CF_3	5.4	0.7	–4.7
S			23.0	4.4	–18.6
Se			32.4	7.5	–24.9
O	CF_3	CF_3	6.8	0.1	–6.7
S			34.0	3.1	–30.9
Se			35.9	15.3	–20.6

[a] Optimized at ZORA-BLYP-D3(BJ)/TZ2P in C_1 symmetry. [b] Change in θ going from the equilibrium geometry of the monomer to that of the hydrogen-bonded complex with formaldehyde (see Figure 2 in the main text for the corresponding structures).

Table S4. XC–$C_{Ar}C_{Ar}$ dihedral angle θ (in degrees; highlighted in blue below) of the 1,3-diaryl symmetrically disubstituted X-urea derivatives (X = O, S, or Se) in the equilibrium geometry of the X-urea monomer ($\theta_{monomer}$) and that of the hydrogen-bonded complex with formaldehyde ($\theta_{complex}$).[a]

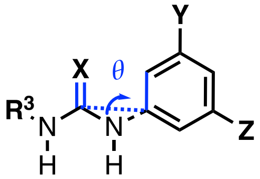

X	R^3	Y	Z	$\theta_{monomer}$	$\theta_{complex}$	$\Delta\theta_{monomer\rightarrow complex}$[b]
O	Ph	H	H	0.6	0.1	–0.5
S				38.0	24.4	–13.6
Se				42.6	34.5	–8.1
O	3-$(CF_3)C_6H_4$	H	CF_3	1.6	0.4	–1.2
S				38.5	6.3	–32.2
Se				41.6	28.6	–13.0
O	3,5-$(CF_3)_2C_6H_3$	CF_3	CF_3	2.6	1.0	–1.6
S				40.0	21.7	–18.3
Se				43.4	35.9	–7.5

[a] Optimized at ZORA-BLYP-D3(BJ)/TZ2P in C_1 symmetry. [b] Change in θ going from the equilibrium geometry of the monomer to that of the hydrogen-bonded complex with formaldehyde (see Figure 3 in the main text for the corresponding structures).

Data S3. Asymmetric formaldehyde–1,3-disubstituted X-urea complexes (X = O, S, Se)

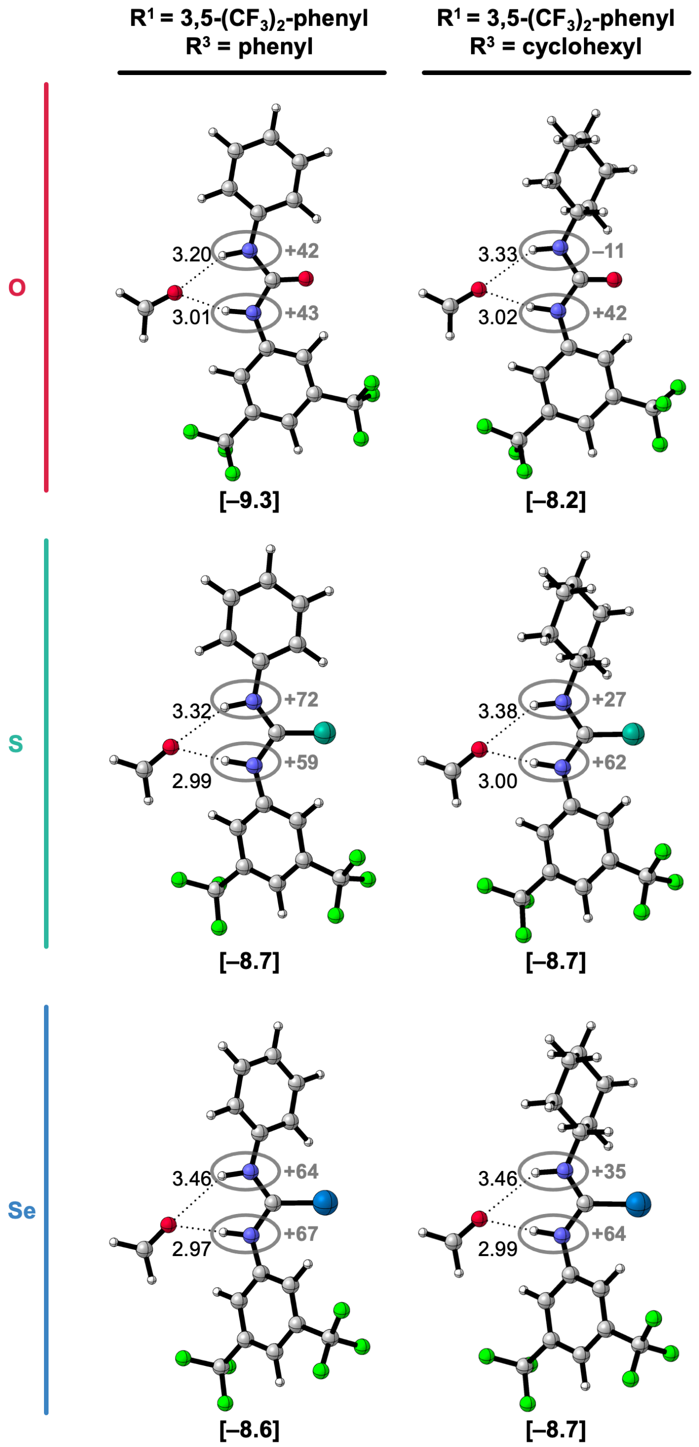

Figure S2. Equilibrium hydrogen-bonded complexes of formaldehyde with asymmetrically 1,3-disubstituted X-urea derivatives (X = O, S, or Se; $R^1 \neq R^3$). The equilibrium hydrogen-bond (O···(H)N) distances (in Å) are indicated and the hydrogen-bond energies ΔE (in kcal mol^{-1}) are shown below the structures between square brackets. The total Voronoi deformation density (VDD) atomic charge Q_{NH} of the NH groups (in milli-electrons) of the isolated X-urea in the geometry of the hydrogen-bonded complex is highlighted in grey. Color code of the ball-and-stick structures: H = white; C = grey; N = dark blue; O = pink; F = green; S = turquoise; Se = light blue.

Table S5. Decomposition of the hydrogen-bond energy ΔE (in kcal mol^{-1}) of the asymmetric formaldehyde–1,3-disubstituted X-urea (X = O, S, or Se) complexes.[a,b,c]

R^1	R^3	X	ΔE	ΔE_{strain}	ΔE_{int}	ΔV_{elstat}	ΔE_{Pauli}	ΔE_{oi}	ΔE_{disp}
3,5-$(CF_3)_2C_6H_3$	Ph	O	–9.3	0.2	–9.5	–10.8	9.6	–5.3	–2.9
		S	–8.7	1.1	–9.8	–11.1	10.6	–5.8	–3.5
		Se	–8.6	0.8	–9.3	–10.8	10.5	–5.6	–3.5
3,5-$(CF_3)_2C_6H_3$	Cyclohexyl	O	–8.2	0.2	–8.4	–9.6	8.9	–4.8	–3.0
		S	–8.7	0.4	–9.1	–10.4	10.0	–5.3	–3.4
		Se	–8.7	0.5	–9.2	–10.6	10.3	–5.5	–3.4

[a] All computed at ZORA-BLYP-D3(BJ)/TZ2P in C_1 symmetry. [b] $\Delta E = \Delta E_{strain} + \Delta E_{int}$; $\Delta E_{int} = \Delta V_{elstat} + \Delta E_{Pauli} + \Delta E_{oi} + \Delta E_{disp}$ (see Method S2 for details). [c] See Figure S2 for the corresponding structures.

Table S6. XC–$C_{Ar}C_{Ar}$ dihedral angle θ (in degrees; highlighted in blue below) of the aromatic 1,3-asymmetrically disubstituted X-urea derivatives (X = O, S, or Se) in the equilibrium geometry of the X-urea monomer ($\theta_{monomer}$) and that of the hydrogen-bonded complex with formaldehyde ($\theta_{complex}$).[a]

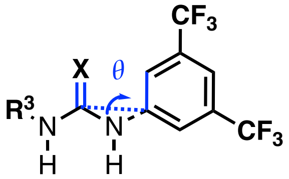

X	R^3	$\theta_{monomer}$	$\theta_{complex}$	$\Delta\theta_{monomer\rightarrow complex}$[b]
O	Ph[c]	1.4 (0.6)	0.8 (0.1)	–0.6 (–0.5)
S		41.1 (35.9)	30.0 (4.8)	–11.1 (–31.1)
Se		43.5 (42.5)	34.3 (34.7)	–9.2 (–7.8)
O	cyclohexyl	5.9	1.1	–4.8
S		37.5	24.0	–13.5
Se		41.0	31.0	–10.0

[a] Optimized at ZORA-BLYP-D3(BJ)/TZ2P in C_1 symmetry. [b] Change in θ going from the equilibrium geometry of the monomer to that of the hydrogen-bonded complex with formaldehyde (see Figure S2 for the corresponding structures). [c] The dihedral angle θ' (in degrees) of the phenyl substituent (*i.e.*, R^3 = Ph) is denoted in between parentheses.

Data S4. Formaldehyde–X-urea complexes (X = C, Si, Ge)

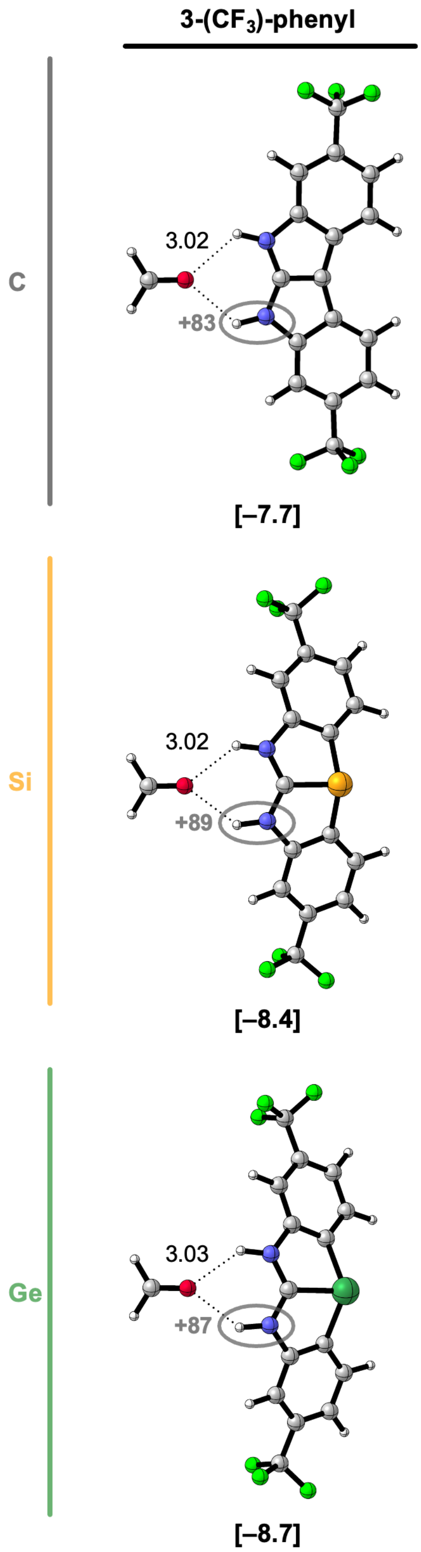

Figure S3. Equilibrium hydrogen-bonded complexes of formaldehyde with 1,3-(3-CF_3-phenyl)-disubstituted predistorted X-urea derivatives (X = C, Si, or Ge). The equilibrium hydrogen-bond (O···(H)N) distances (in Å) are indicated and the hydrogen-bond energies ΔE (in kcal mol^{-1}) are shown below the structures between square brackets. The total Voronoi deformation density (VDD) atomic charge Q_{NH} of the NH groups (in milli-electrons) of the isolated X-urea in the geometry of the hydrogen-bonded complex is highlighted in grey. Color code of the ball-and-stick structures: H = white; C = grey; N = dark blue; O = pink; F = green; Si = yellow; Ge = dark green.

Table S7. Decomposition of the hydrogen-bond energy ΔE (in kcal mol^{-1}) of the formaldehyde–1,3-disubstituted predistorted X-urea (X = C, Si, or Ge) complexes.[a,b,c]

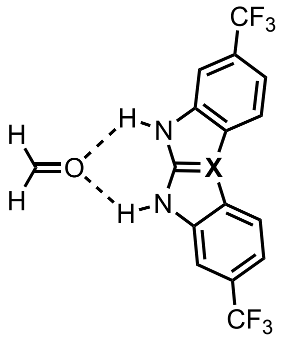

X	ΔE	ΔE_{strain}	ΔE_{int}	ΔV_{elstat}	ΔE_{Pauli}	ΔE_{oi}	ΔE_{disp}
C	–7.7	0.3	–8.0	–8.7	5.8	–3.0	–2.1
Si	–8.4	0.3	–8.7	–10.1	7.7	–4.1	–2.3
Ge	–8.7	0.2	–8.9	–10.6	8.4	–4.4	–2.3

[a] All computed at ZORA-BLYP-D3(BJ)/TZ2P in C_1 symmetry. [b] $\Delta E = \Delta E_{strain} + \Delta E_{int}$; $\Delta E_{int} = \Delta V_{elstat} + \Delta E_{Pauli} + \Delta E_{oi} + \Delta E_{disp}$. [c] See Figure S3 for the corresponding structures.

Data S5. 1,3-bis[3-(CF_3)phenyl]urea vs. 1,3-bis[4-(CF_3)phenyl]urea

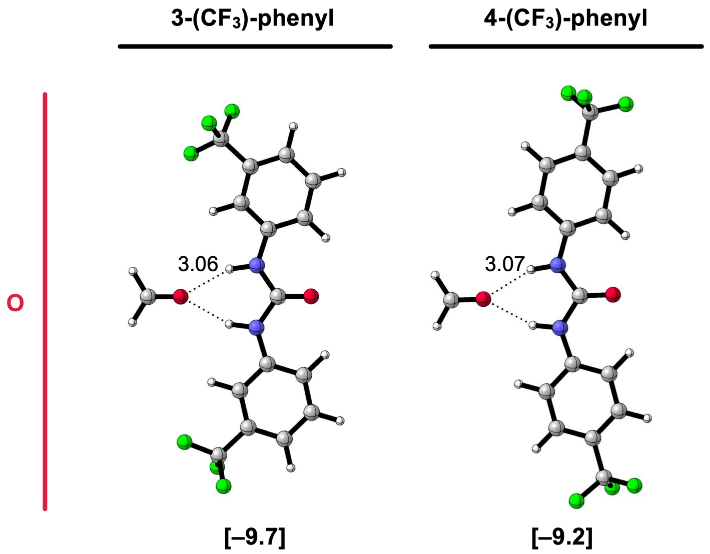

Figure S4. Equilibrium hydrogen-bonded complexes of formaldehyde with 1,3-[*n*-$(CF_3)_2$phenyl]urea [X = O; *n* = 3 (*meta*) or 4 (*para*)]. The equilibrium hydrogen-bond (O···(H)N) distances (in Å) are indicated and the hydrogen-bond energies ΔE (in kcal mol^{-1}) are shown below the structures between square brackets. Color code of the ball-and-stick structures: H = white; C = grey; N = blue; O = pink; F = green.

Table S8. Decomposition of the hydrogen-bond energy ΔE (in kcal mol^{-1}) of the formaldehyde–1,3-[*n*-$(CF_3)_2$phenyl]urea [X = O; *n* = 3 (*meta*) or 4 (*para*)] complexes.[a,b,c]

R^1	R^3	X	ΔE	ΔE_{strain}	ΔE_{int}	ΔV_{elstat}	ΔE_{Pauli}	ΔE_{oi}	ΔE_{disp}
3-(CF_3)C_6H_4	3-(CF_3)C_6H_4	O	–9.7	0.2	–9.9	–11.3	9.6	–5.4	–2.7
4-(CF_3)C_6H_4	4-(CF_3)C_6H_4	O	–9.2	0.1	–9.3	–10.8	9.2	–5.1	–2.6

[a] All computed at ZORA-BLYP-D3(BJ)/TZ2P in C_1 symmetry. [b] $\Delta E = \Delta E_{strain} + \Delta E_{int}$; $\Delta E_{int} = \Delta V_{elstat} + \Delta E_{Pauli} + \Delta E_{oi} + \Delta E_{disp}$ (see Method S2 for details). [c] See Figure S4 for the corresponding structures.

Data S6. Cartesian coordinates and energies of optimized monomers

Below is a list consisting of the Cartesian coordinates (in Å), ADF total bond energies *E*, enthalpies *H*, Gibbs free energies *G* (in kcal mol^{-1}), and the number of imaginary frequencies (N_{imag}) of all optimized monomers reported in this work, optimized at the ZORA-BLYP-D3(BJ)/TZ2P level.

Formaldehyde

```
E: −494.36
H: −475.92
G: −491.92
Nimag = 0

H      1.17359952       0.94518045      -0.00137433
H      1.17359952      -0.94518045      -0.00137433
C      0.58285140       0.00000000      -0.00078141
O     -0.62895043       0.00000000       0.00043007
```

Urea

```
E: −1059.79
H: −1017.84
G: −1037.83
Nimag = 0

N      1.19058131      -0.51423267      -0.20589918
N     -1.10856075      -0.57298645       0.23787953
C      0.01965806       0.21608292       0.00597078
O     -0.01479062       1.44263088      -0.01017708
H      2.03030398       0.05252568      -0.15736625
H     -1.97879583      -0.05584540       0.17508885
H      1.27162565      -1.42924204       0.22558231
H     -1.13771448      -1.50223177      -0.16910903
```

Thiourea

```
E: −981.33
H: −940.56
G: −961.48
Nimag = 0

N      1.18058694      -0.48209422      -0.17255048
N     -1.10037175      -0.54121322       0.20366322
C      0.01918647       0.23320458       0.00580741
S     -0.02789530       1.90661262      -0.01635258
H      2.03215443       0.06588172      -0.16253884
H     -1.98139857      -0.04246125       0.17984260
H      1.25089122      -1.42476688       0.19942532
H     -1.11725683      -1.49590631      -0.14306214
```

Selenourea
E: −960.00
H: −919.61
G: −941.61
N_{imag} = 0

N	1.18308681	−0.47556264	−0.14830592
N	−1.10327084	−0.53548190	0.17917930
C	0.01949367	0.22112692	0.00591295
Se	−0.03210354	2.06244690	−0.01833854
H	2.03530979	0.07029821	−0.15012574
H	−1.98490549	−0.03868044	0.16719893
H	1.25510632	−1.44235687	0.15519136
H	−1.12048046	−1.51259614	−0.09820320

1-Cyclohexylurea
E: −3083.77
H: −2946.20
G: −2975.80
N_{imag} = 0

N	8.23146854	6.13271249	−14.31365898
N	10.03288949	4.86140649	−13.52989913
C	9.34282615	5.37353498	−14.64077448
H	4.56513732	5.07251824	−17.00449070
O	9.70001496	5.14557105	−15.79660773
H	5.81742183	6.08331274	−17.72290031
H	3.90175003	6.91551309	−15.46954316
H	10.95279432	4.51066520	−13.77681244
H	7.84907784	6.00952246	−13.38069314
H	10.02035813	5.41073956	−12.67550539
H	3.85204588	7.49109724	−17.13670227
C	7.27308715	6.54444426	−15.35011753
C	5.51482183	8.27549243	−15.96938790
C	6.52831832	7.81446984	−14.90307402
C	6.28437396	5.41639786	−15.71573367
C	5.27715937	5.87875579	−16.78638088
C	4.52960063	7.15084143	−16.34285317
H	7.87680383	6.77320882	−16.23606782
H	6.06240918	8.58940980	−16.87073187
H	4.97035361	9.15765041	−15.60881060
H	5.99297942	7.60375385	−13.96258059
H	7.25489196	8.60807972	−14.68852301
H	5.74119649	5.11626054	−14.80537702
H	6.85009169	4.54462163	−16.06307237

1-Cyclohexylthiourea
E: −3006.43
H: −2870.05
G: −2900.37
N_{imag} = 0

N	8.22872046	6.05720767	−14.37802963
N	10.05384571	4.93469487	−13.52208257
C	9.37498153	5.37077167	−14.64916197
H	4.51225551	5.09968755	−17.01779367
S	9.94551285	5.02990762	−16.19447427
H	5.76992839	6.10093544	−17.74031108
H	3.90142656	6.92933686	−15.44540894
H	10.98516398	4.58276233	−13.71271600
H	7.88370471	6.02944666	−13.42200312
H	9.96302611	5.47073021	−12.66222879
H	3.83549278	7.53125568	−17.10257450
C	7.26437532	6.50577876	−15.39163534

C	5.52741898	8.27214202	−15.95039586
C	6.55112998	7.78009615	−14.90769513
C	6.25500859	5.39610410	−15.75167216
C	5.23995308	5.89145968	−16.79925903
C	4.51948827	7.16805440	−16.32473702
H	7.85897659	6.73510667	−16.28359111
H	6.06576310	8.59167795	−16.85501193
H	5.00293039	9.15651601	−15.56683664
H	6.02822815	7.56456055	−13.96113451
H	7.29426268	8.55946298	−14.69684818
H	5.72282276	5.09095489	−14.83691370
H	6.80543455	4.52412212	−16.12134939

1-Cyclohexylselenourea

E: −2985.75
H: −2849.72
G: −2881.02
N_{imag} = 0

N	8.22088023	6.04199606	−14.37863517
N	10.06859418	4.95316075	−13.53356372
C	9.37669739	5.38637301	−14.63968082
H	4.50029699	5.10178613	−17.01327532
Se	10.02611144	5.03547053	−16.33844884
H	5.76432739	6.09396365	−17.73741190
H	3.89894518	6.93871757	−15.44546561
H	11.00132522	4.60445613	−13.72064374
H	7.88583672	6.04572627	−13.41770490
H	9.93586017	5.42123554	−12.63992591
H	3.83729370	7.53702655	−17.10405246
C	7.25731168	6.49728107	−15.39150538
C	5.53255719	8.27136396	−15.95313042
C	6.55330223	7.77645686	−14.90867114
C	6.24380619	5.39123713	−15.74738417
C	5.23221559	5.89004057	−16.79646933
C	4.51880647	7.17178388	−16.32505225
H	7.85450280	6.72009358	−16.28381122
H	6.07342230	8.58540203	−16.85804165
H	5.01301813	9.15956472	−15.57188416
H	6.02926164	7.56673558	−13.96133741
H	7.30132479	8.55206785	−14.70109740
H	5.70998765	5.08950336	−14.83248118
H	6.79188500	4.51725699	−16.11624676

1-Phenylurea

E: −2572.45
H: −2478.37
G: −2506.29
N_{imag} = 0

N	1.18494757	−0.86116706	−0.18403123
N	−1.09429938	−0.39563189	−0.15025547
C	0.19384051	0.12756729	−0.08840853
C	−2.32956230	0.28864858	−0.08678747
C	−2.43955252	1.67043429	0.15002307
C	−3.70749621	2.25616177	0.21089530
C	−4.86927995	1.49705472	0.03953504
C	−4.75510841	0.12237193	−0.19754018
C	−3.49806222	−0.47797955	−0.26029246
O	0.46628337	1.32147447	−0.00756059
H	−3.41718291	−1.54912950	−0.44402401
H	−5.64565903	−0.48696269	−0.33472631
H	−3.78113546	3.32613629	0.39382171
H	2.10853419	−0.50252154	0.03490497

H	0.99535594	-1.77876570	0.20732625
H	-1.17005236	-1.38441364	-0.36146645
H	-1.54245050	2.26230425	0.27442225
H	-5.84826550	1.96719920	0.08843030

1-Phenylthiourea

E: −2492.34
H: −2400.07
G: −2427.07
N_{imag} = 0

N	1.14711257	-0.89746257	-0.10323254
N	-1.07260590	-0.33905566	-0.05767201
C	0.21430901	0.12569699	-0.21617699
C	-2.31973878	0.33327965	-0.04365836
C	-2.47303444	1.68702371	0.29501868
C	-3.75313153	2.24699525	0.32152200
C	-4.88556943	1.48210348	0.02408534
C	-4.73025614	0.13033388	-0.30287565
C	-3.45803623	-0.44005996	-0.33825500
S	0.69133046	1.68626119	-0.58334999
H	-3.34111942	-1.49097450	-0.60145335
H	-5.59815010	-0.48193562	-0.53665291
H	-3.86067127	3.29755954	0.58229009
H	2.10851262	-0.57584932	-0.10774049
H	0.95431143	-1.66414431	0.53709373
H	-1.16627425	-1.34980779	-0.10950201
H	-1.60154246	2.28820975	0.51786875
H	-5.87528187	1.93136208	0.04699812

1-Phenylselenourea

E: −2470.96
H: −2379.07
G: −2406.96
N_{imag} = 0

N	-5.28782393	-1.21310921	0.45076443
N	-3.28803396	-0.29198378	-0.16730199
C	-4.17578006	-0.50620865	0.85348627
C	-2.06445354	0.42613630	-0.21075575
C	-1.79931619	1.54466257	0.59301808
C	-0.58257256	2.21663647	0.45250328
C	0.37003403	1.79879904	-0.48293461
C	0.09542071	0.69150803	-1.29294584
C	-1.11321525	0.00733200	-1.15720448
Se	-3.98126321	-0.04175505	2.62446292
H	-1.32003136	-0.86191021	-1.78059026
H	0.82322432	0.35318938	-2.02674035
H	-0.38325877	3.08083271	1.08220975
H	-6.00525793	-1.31605224	1.15903112
H	-5.61706379	-1.13013632	-0.50844375
H	-3.44482973	-0.86037266	-0.99646557
H	-2.53034639	1.86859157	1.32265510
H	1.31358092	2.32981925	-0.58150769

1-[3-(trifluoromethyl)phenyl]urea

Global minimum conformer
E: −2967.31
H: −2869.10
G: −2901.48
N_{imag} = 0

N	−0.98601782	0.21674962	0.23102304
N	−3.17670547	−0.41161402	0.69660829
C	−2.26612757	0.64364133	0.58822818
C	3.82915065	0.25280447	−0.72873758
O	−2.57236914	1.80611255	0.83192370
F	4.66843045	0.91782394	−1.58271190
F	3.60886003	−0.98928037	−1.26961455
H	−4.13596992	−0.09578145	0.79116970
H	−0.83571663	−0.78538409	0.19479727
H	−3.05949827	−1.23054669	0.10866172
F	4.53921100	0.04839524	0.43425881
H	1.36623339	−0.77916706	−0.30774427
C	0.17268321	0.99546288	0.03489726
C	0.18607554	2.40101633	0.09806311
C	1.38204254	3.08648966	−0.12753432
C	2.56930269	2.41005807	−0.41342980
C	2.54850445	1.01085963	−0.47538466
C	1.36495570	0.30671354	−0.25167151
H	−0.72929399	2.93110130	0.32501760
H	1.38214949	4.17273608	−0.07825394
H	3.49196042	2.95343782	−0.59028419

Local minimum conformer
E: −2966.90
H: −2868.71
G: −2901.04
N_{imag} = 0

N	−0.98601782	0.21674962	0.23102304
N	−3.17670547	−0.41161402	0.69660829
C	−2.26612757	0.64364133	0.58822818
C	3.82915065	0.25280447	−0.72873758
O	−2.57236914	1.80611255	0.83192370
F	4.66843045	0.91782394	−1.58271190
F	3.60886003	−0.98928037	−1.26961455
H	−4.13596992	−0.09578145	0.79116970
H	−0.83571663	−0.78538409	0.19479727
H	−3.05949827	−1.23054669	0.10866172
F	4.53921100	0.04839524	0.43425881
H	1.36623339	−0.77916706	−0.30774427
C	0.17268321	0.99546288	0.03489726
C	0.18607554	2.40101633	0.09806311
C	1.38204254	3.08648966	−0.12753432
C	2.56930269	2.41005807	−0.41342980
C	2.54850445	1.01085963	−0.47538466
C	1.36495570	0.30671354	−0.25167151
H	−0.72929399	2.93110130	0.32501760
H	1.38214949	4.17273608	−0.07825394
H	3.49196042	2.95343782	−0.59028419

1-[3-(trifluoromethyl)phenyl]thiourea

Global minimum conformer
E: −2886.85
H: −2790.46
G: −2821.73
N_{imag} = 0

N	−0.96071793	0.18997918	0.15412652
N	−3.12466787	−0.38283818	0.62882461
C	−2.16223795	0.61172432	0.68866322
F	4.55940233	−0.35473008	0.56060975
S	−2.48729674	2.08273711	1.41283961
F	4.74878836	0.24424925	−1.54766542
F	3.54930124	−1.52432478	−1.00736022
H	−4.04872127	−0.08573811	0.92048649
H	−0.89271996	−0.81327004	0.00608075
H	−3.10336679	−1.04988925	−0.13845130
H	1.74044932	3.90430420	−0.41581909
H	−0.47465718	2.88801711	0.02283802

C	0.26544818	0.86353314	-0.04488884
C	1.40104245	0.05463559	-0.22665017
C	2.64544725	0.63320631	-0.47669312
C	2.78284076	2.02601344	-0.53899180
C	1.65046902	2.82223005	-0.36077658
C	0.39470548	2.26012194	-0.11914120
H	1.31145612	-1.02779478	-0.17596421
C	3.86454691	-0.24561013	-0.62286695
H	3.75314866	2.47303518	-0.73112589

Local minimum conformer
E: -2886.54
H: -2789.60
G: -2822.86
N_{imag} = 0

N	1.15093403	-0.90731841	-0.03967222
N	-1.07806389	-0.38234696	0.00062154
C	0.20370661	0.08479354	-0.22901987
C	-2.31679158	0.29819517	-0.04009061
C	-2.45337483	1.65172967	0.30397091
C	-3.72188585	2.23607133	0.28390889
C	-4.86039717	1.49872223	-0.06735822
C	-4.71730755	0.14919322	-0.39331912
C	-3.45657001	-0.44835708	-0.38351101
F	-2.82178528	4.19609806	1.33611177
C	-3.87000040	3.70536909	0.60894090
H	2.10987499	-0.58516558	-0.10249559
H	0.97833544	-1.62817785	0.65658504
H	-3.35144256	-1.49902423	-0.64997193
H	-1.17023012	-1.39443939	-0.00033726
H	-1.58374416	2.23306081	0.57659235
H	-5.83787240	1.97060888	-0.07735924
S	0.63115527	1.61208225	-0.75002971
F	-3.95393538	4.47007387	-0.53200335
F	-5.01214293	3.95936211	1.32849216
H	-5.58966187	-0.44048841	-0.66302472

1-[3-(trifluoromethyl)phenyl]selenourea

Global minimum conformer
E: -2865.41
H: -2768.83
G: -2802.83
N_{imag} = 0

N	-0.97390169	0.17540627	0.00946081
N	-3.19844933	-0.29675909	0.24918146
C	-2.20686559	0.64194098	0.39646532
F	4.33885539	-0.16708317	1.59000799
Se	-2.59278538	2.28269782	1.13313402
F	4.97978398	0.12586291	-0.49402451
F	3.68717017	-1.58031401	0.03297850
H	-4.13648747	0.02607227	0.45435371
H	-0.90687478	-0.83511092	-0.08753078
H	-3.11318916	-1.02835925	-0.45213386
H	1.80476114	3.80740800	-0.65177209
H	-0.45694013	2.80597897	-0.49302136
C	0.28131041	0.82865324	-0.05197539
C	1.42319460	0.03466129	0.13341738
C	2.69532842	0.60230958	0.03331404
C	2.84399370	1.96803207	-0.23799060
C	1.70158141	2.74890778	-0.42656974
C	0.42315903	2.19388125	-0.34374193
H	1.31983363	-1.02420830	0.35681986

C	3.91593706	−0.25051427	0.28266662
H	3.83429035	2.40658344	−0.31071060

Local minimum conformer
E: −2865.14
H: −2768.60
G: −2802.71
N_{imag} = 0

N	1.15397308	−0.87471012	−0.00058249
N	−1.08330188	−0.39369899	0.05648966
C	0.17864281	0.06018003	−0.24837569
C	−2.32476603	0.28618686	−0.01488818
C	−2.45710015	1.63424093	0.34768521
C	−3.71676946	2.23344604	0.29382836
C	−4.84924542	1.51133395	−0.10872067
C	−4.71032554	0.16559336	−0.44880844
C	−3.45524917	−0.44597644	−0.40627957
F	−2.81462875	4.17839597	1.37080776
C	−3.86585889	3.69679050	0.64230426
H	2.10526195	−0.55536075	−0.14043295
H	1.01489969	−1.57594775	0.72302409
H	−3.34866117	−1.49206723	−0.68854279
H	−1.17322521	−1.40295510	0.14633151
H	−1.59051560	2.19928116	0.66276260
H	−5.82105744	1.99384438	−0.14566347
Se	0.60746131	1.68354122	−0.99452668
F	−3.96004381	4.47894976	−0.48536107
F	−5.00470363	3.93567366	1.37330285
H	−5.57921057	−0.40987768	−0.75735461

1-[3,5-bis(trifluoromethyl)phenyl]urea
E: −3360.34
H: −3258.05
G: −3294.75
N_{imag} = 0

N	1.18104315	−0.84083017	−0.17069363
N	−1.10635984	−0.41665849	−0.14585107
C	0.18229727	0.12647749	−0.07121059
C	−2.33620403	0.26149402	−0.08137036
C	−2.44800600	1.63543085	0.19125432
C	−3.71720012	2.21895181	0.25235560
C	−4.88059489	1.47545301	0.04509787
C	−4.75676284	0.10890011	−0.22754961
C	−3.50328621	−0.49658041	−0.28961787
C	−6.00168361	−0.73239304	−0.39797946
C	−3.81180818	3.71066503	0.49906452
H	2.10688862	−0.48233659	0.03655775
H	1.00397223	−1.78224798	0.16362183
H	−3.43017542	−1.55980967	−0.50407039
H	−1.17318713	−1.40215554	−0.37492524
H	−1.55359284	2.22391986	0.34487237
H	−5.85614208	1.94468573	0.09294555
O	0.42415404	1.32393897	0.02432491
F	−7.01773437	−0.03924918	−0.99466793
F	−6.48215675	−1.17165465	0.81437873
F	−5.77476619	−1.85197665	−1.15602837
F	−2.89934093	4.13959556	1.42372952
F	−5.04898052	4.08656703	0.94758709
F	−3.57386303	4.42436977	−0.65156998

1-[3,5-bis(trifluoromethyl)phenyl]thiourea

E: −3279.74
H: −3178.73
G: −3216.28
N_{imag} = 0

N	1.14177741	−0.88213372	−0.05072534
N	−1.09319474	−0.39397766	0.06615654
C	0.17032402	0.07339743	−0.26863864
C	−2.33155767	0.28133747	0.02766360
C	−2.45789874	1.64697259	0.32885009
C	−3.72250791	2.23683311	0.31089826
C	−4.87107341	1.49657445	0.00912685
C	−4.73544919	0.13600672	−0.27213813
C	−3.47690168	−0.47116617	−0.26793991
C	−5.95911732	−0.71111290	−0.53854124
C	−3.85218356	3.72059388	0.58427249
H	2.09269227	−0.56460110	−0.19761303
H	1.01267457	−1.57139754	0.68512391
H	−3.38645888	−1.52765476	−0.50795428
H	−1.17664241	−1.40533317	0.12252196
H	−1.58158402	2.23242170	0.57015044
H	−5.84686595	1.96826591	−0.00085044
S	0.52938978	1.56146725	−0.93099415
F	−7.01548359	0.02426603	−0.99334616
F	−6.38311373	−1.35325363	0.60329323
F	−5.71548410	−1.69158862	−1.46612038
F	−2.84276389	4.19526112	1.37442278
F	−5.03316026	4.02560977	1.20894886
F	−3.82341006	4.44897153	−0.57999776

1-[3,5-bis(trifluoromethyl)phenyl]selenourea

E: −3258.31
H: −3157.69
G: −3196.02
N_{imag} = 0

N	1.14254632	−0.86615821	−0.02684356
N	−1.09348564	−0.39440086	0.09232798
C	0.16011654	0.06003906	−0.25867270
C	−2.33495797	0.27970219	0.04684137
C	−2.45989720	1.64387347	0.35232387
C	−3.72221990	2.23731881	0.31923037
C	−4.86830420	1.49946583	0.00015550
C	−4.73370869	0.13922143	−0.28079890
C	−3.47571573	−0.47119842	−0.26292424
C	−5.95510973	−0.70572629	−0.56258007
C	−3.85561503	3.71987500	0.59591558
H	2.09033092	−0.55893733	−0.20922696
H	1.02200155	−1.58338154	0.68370842
H	−3.38356201	−1.52698039	−0.50523154
H	−1.17802303	−1.40274355	0.19700903
H	−1.58434492	2.22440101	0.60869670
H	−5.84284935	1.97363148	−0.02136446
Se	0.55709829	1.67367894	−1.04052413
F	−7.01246146	0.03395247	−1.00728671
F	−6.37903052	−1.36719448	0.56844510
F	−5.70737857	−1.67113832	−1.50490045
F	−2.82988647	4.20292733	1.35854156
F	−5.02271529	4.01488481	1.25193380
F	−3.86526838	4.44779574	−0.56880436

1,3-Dicyclohexylurea

E: −5107.43
H: −4874.29
G: −4913.45
N_{imag} = 0

N	2.94169612	−0.64143172	0.53965770
N	0.71154619	−0.19633054	0.00255798
C	1.88750428	0.26799830	0.58777661
H	5.11020146	−2.24427355	0.69878214
H	5.09322336	−0.88523446	4.68071369
H	5.47902902	−0.84280471	−0.31437620
H	3.67241308	−1.86181948	2.87796595
O	1.98648287	1.38521433	1.10279357
H	5.46994137	0.50494268	3.66487019
H	6.26340362	−2.40869504	3.09884148
H	3.12731454	−0.21535358	3.24007324
H	−3.83128286	0.32075955	−0.74342293
H	2.69044271	−1.61705291	0.40520752
H	0.80954738	−0.95751157	−0.66384666
H	7.35759139	−1.11348102	3.58734056
C	4.15170879	−0.41308169	1.34316398
C	6.63194825	−0.94700726	1.54038805
C	5.34228055	−1.16672603	0.72485992
C	3.95738932	−0.79864241	2.82588711
C	5.24612600	−0.57216249	3.63982215
C	6.44037586	−1.32464110	3.02222307
H	4.34179777	0.66519032	1.29102924
H	6.92128613	0.11254655	1.47294603
H	7.45430814	−1.52627234	1.10100021
C	−2.66791199	1.75256859	−1.90803028
H	−1.64792787	−0.78369613	−1.17315790
H	−1.15988928	3.08978367	−2.74249970
H	−1.88166114	−0.59928476	0.56981338
H	−0.02213416	0.89712753	−2.39627742
H	−1.40357279	3.26569153	−1.00581102
H	−2.64280905	1.09176663	−2.78841355
H	0.77676426	2.14322103	−1.42254630
H	−3.50402060	2.44790677	−2.05855681
C	−0.39448103	0.73332785	−0.27060405
C	−2.90596833	0.90290486	−0.64452661
C	−1.72084360	−0.04146986	−0.36110525
C	−0.15496388	1.57864772	−1.54060779
C	−1.33836787	2.52591665	−1.81781311
H	−0.43098382	1.41045531	0.59060872
H	−3.04463364	1.57138458	0.21850596

1,3-Dicyclohexylthiourea

E: −5030.67
H: −4798.75
G: −4838.59
N_{imag} = 0

N	2.92201346	−0.48767717	0.66657360
N	0.76994553	−0.00402649	0.01750933
C	1.90093920	0.43011076	0.66347672
H	5.01315831	−2.19282221	0.66723876
H	5.18131009	−1.02637942	4.70905342
H	5.41753074	−0.75874879	−0.28811218
H	3.66107000	−1.85158496	2.91045551
S	2.02477712	1.95169072	1.38691141
H	5.58969713	0.39209357	3.74582231
H	6.22977992	−2.52484002	3.02126422
H	3.20576964	−0.19893599	3.36397033

H	-3.78408171	0.16992100	-0.78415219
H	2.69041789	-1.43734000	0.38525707
H	0.85596447	-0.84306723	-0.55103864
H	7.39801093	-1.30505888	3.53177830
C	4.16642940	-0.35070928	1.43449822
C	6.61885277	-1.00899965	1.52039118
C	5.29596212	-1.12921432	0.73756652
C	3.99419661	-0.80156723	2.90008102
C	5.31667429	-0.67162081	3.67931759
C	6.45469233	-1.44746503	2.98870937
H	4.40353535	0.71919338	1.42835740
H	6.95574581	0.03792241	1.49192020
H	7.39801882	-1.60432180	1.02732160
C	-2.70836341	1.65275152	-1.96970521
H	-1.52608816	-0.79081510	-1.16151998
H	-1.28599373	3.07131358	-2.81992476
H	-1.79372886	-0.57767784	0.57506174
H	-0.00379873	0.97027816	-2.41006616
H	-1.56034887	3.26948136	-1.09013593
H	-2.62768171	0.97538361	-2.83402756
H	0.69510907	2.29068261	-1.45499703
H	-3.58870366	2.28476714	-2.14467944
C	-0.39502310	0.83753060	-0.28499569
C	-2.90328288	0.81782671	-0.68905636
C	-1.65941202	-0.03529988	-0.36929577
C	-0.19391584	1.66177343	-1.57382533
C	-1.43636513	2.51800765	-1.88409480
H	-0.48707059	1.53200424	0.55772289
H	-3.09839158	1.49411194	0.15646013

1,3-Dicyclohexylselenourea

E: −5010.40
H: −4778.80
G: −4819.57
N_{imag} = 0

N	2.91298434	-0.48487047	0.69239822
N	0.78267925	0.01669731	-0.00048797
C	1.90100497	0.42787181	0.66156772
H	5.01326834	-2.18141914	0.66066838
H	5.19100071	-1.05896682	4.71573532
H	5.40691458	-0.73295300	-0.27879897
H	3.66718721	-1.87277744	2.91474816
Se	2.03590427	2.10430280	1.46084376
H	5.58687958	0.37237165	3.76629408
H	6.24124113	-2.53308138	3.00759715
H	3.20626016	-0.22589789	3.38642077
H	-3.77833719	0.16659408	-0.76816298
H	2.70987559	-1.42241539	0.35189097
H	0.83997762	-0.85569795	-0.52184824
H	7.40456383	-1.31220639	3.52652261
C	4.16217131	-0.35217241	1.45486727
C	6.61608538	-0.99880741	1.52179239
C	5.29075082	-1.11713193	0.74255158
C	3.99554116	-0.82129887	2.91449593
C	5.32034887	-0.69220699	3.68951040
C	6.45982946	-1.45412579	2.98585931
H	4.39306566	0.71942254	1.46084575
H	6.94706226	0.05010520	1.50325315
H	7.39611701	-1.58468974	1.01900750
C	-2.71614320	1.64455446	-1.97214358
H	-1.52075968	-0.79067786	-1.15489297
H	-1.30450222	3.06121564	-2.84321383
H	-1.77608630	-0.56360019	0.58258387

H	−0.01225871	0.96691458	−2.42932316
H	−1.56504776	3.27034952	−1.11252097
H	−2.64049161	0.96149938	−2.83244356
H	0.68897326	2.29590388	−1.48642462
H	−3.59967514	2.27282160	−2.14430297
C	−0.38984173	0.85011394	−0.30038182
C	−2.89905863	0.81801061	−0.68427890
C	−1.65022575	−0.02922282	−0.36759414
C	−0.19879616	1.66351725	−1.59664678
C	−1.44601643	2.51402613	−1.90250038
H	−0.47678959	1.55141927	0.53764573
H	−3.08952186	1.49938015	0.15806215

1,3-Diphenylurea

E: −4085.03
H: −3938.84
G: −3974.97
N_{imag} = 0

N	0.86627103	1.13945697	−0.45165770
N	−1.21068196	0.14554469	−0.67193359
C	−0.39850712	0.96513550	0.11610078
C	−2.54156078	−0.26678900	−0.43554385
C	−3.13166213	−1.10328361	−1.40363959
C	−4.44082268	−1.55603354	−1.24680899
C	−5.18618316	−1.18337008	−0.12219772
C	−4.59885715	−0.35313107	0.83724605
C	−3.28734225	0.11013136	0.69591208
C	1.95769988	1.89083000	0.03960543
C	3.13592658	1.88404589	−0.73299065
C	4.25902586	2.59950169	−0.32041820
C	4.22870456	3.33521287	0.86997618
C	3.05846932	3.34094087	1.63506969
C	1.92306489	2.62927602	1.23624968
H	3.02006846	3.90832427	2.56258496
H	−2.55817451	−1.39826693	−2.28229807
O	−0.75271150	1.47038422	1.17690315
H	1.05279281	0.66705086	−1.32888302
H	−2.83806888	0.75233899	1.44108792
H	−0.81486165	−0.21347043	−1.53328129
H	−4.87745333	−2.20112322	−2.00597439
H	−6.20720354	−1.53518087	0.00226406
H	−5.16609972	−0.05518680	1.71653010
H	3.16815910	1.31352377	−1.66111021
H	5.15856405	2.58063886	−0.93164307
H	5.10323626	3.89379986	1.19387185
H	1.02076833	2.63678137	1.83239640

1,3-Diphenylthiourea

E: −4003.03
H: −3858.19
G: −3894.89
N_{imag} = 0

N	0.80577305	1.20105324	−0.39780870
N	−1.19000653	0.12703447	−0.55087866
C	−0.42356425	0.99526525	0.21879062
C	−2.55037124	−0.24395133	−0.41011562
C	−2.90707111	−1.55559809	−0.76707387
C	−4.24018880	−1.96619192	−0.70821331
C	−5.23155610	−1.07520267	−0.28413613
C	−4.87400891	0.23165994	0.06683778
C	−3.54544399	0.65667556	0.00171917
C	1.95295747	1.88171081	0.08091728

C	2.73152053	2.58234785	−0.85605090
C	3.90487337	3.22647722	−0.45911808
C	4.31021164	3.18601045	0.87900533
C	3.53537954	2.48351903	1.80910165
C	2.36569377	1.82647257	1.42157359
H	3.84516231	2.43868476	2.85081752
H	−2.13465968	−2.25565119	−1.08327927
S	−0.90432764	1.68316016	1.65932012
H	0.80798065	1.05743155	−1.40396821
H	−3.27722904	1.66956193	0.27364522
H	−0.65698368	−0.49724169	−1.15015288
H	−4.49977262	−2.98517621	−0.98586301
H	−6.26939135	−1.39441694	−0.22964021
H	−5.63762259	0.93541668	0.39054040
H	2.40958355	2.62819199	−1.89572647
H	4.49525241	3.76644768	−1.19578970
H	5.21905559	3.69357759	1.19262302
H	1.77236345	1.28432187	2.14672201

1,3-Diphenylselenourea

E: −3981.41
H: −3836.94
G: −3874.46
N_{imag} = 0

N	0.80052530	1.21148778	−0.41911726
N	−1.17827865	0.10971417	−0.54753586
C	−0.42048485	0.99730963	0.19271303
C	−2.54266847	−0.25610762	−0.40932793
C	−2.88836136	−1.59353439	−0.66065254
C	−4.22310253	−2.00015129	−0.59557343
C	−5.22268503	−1.07742684	−0.27109645
C	−4.87442368	0.25641888	−0.02709873
C	−3.54479072	0.67577107	−0.10002549
C	1.94950827	1.88933382	0.06628301
C	2.68500652	2.66504665	−0.84360776
C	3.85524077	3.30912194	−0.43458724
C	4.29583121	3.19082992	0.88737952
C	3.56258192	2.41120691	1.79000232
C	2.39786192	1.75449822	1.38876081
H	3.90195943	2.30579965	2.81796319
H	−2.10801722	−2.31592357	−0.89614717
Se	−0.95620064	1.78008237	1.76406683
H	0.81277964	1.05334630	−1.42391392
H	−3.27988594	1.70881318	0.08776095
H	−0.64453057	−0.52152360	−1.14037711
H	−4.47708044	−3.03970119	−0.78927438
H	−6.26138874	−1.39301498	−0.21134925
H	−5.64546331	0.98356560	0.21736571
H	2.33178007	2.77193900	−1.86828417
H	4.41422748	3.90998188	−1.14814625
H	5.20152817	3.69812560	1.21056604
H	1.83443354	1.15051270	2.08916626

1,3-bis[3-(trifluoromethyl)phenyl]urea

Global minimum conformer
E: −4874.27
H: −4720.35
G: −4762.50
N_{imag} = 0

N	0.00890112	1.15686929	0.21307243
N	−0.00890112	−1.15686929	0.21307243
C	0.00000000	−0.00000000	0.99695622

C	−0.02970946	−2.50164169	0.64047012
C	−0.00955867	−3.48574847	−0.36443999
C	−0.03730340	−4.83831815	−0.02569332
C	−0.08070345	−5.23695346	1.31654442
C	−0.09954517	−4.25474000	2.30779701
C	−0.07438641	−2.89437853	1.99046364
C	0.02970946	2.50164169	0.64047012
C	0.00955867	3.48574847	−0.36443999
C	0.03730340	4.83831815	−0.02569332
C	0.08070345	5.23695346	1.31654442
C	0.09954517	4.25474000	2.30779701
C	0.07438641	2.89437853	1.99046364
F	0.40223200	5.41401579	−2.32586054
H	0.02105562	−3.19480781	−1.41171317
O	0.00000000	0.00000000	2.22322074
H	−0.01888167	1.04122719	−0.79375932
H	−0.08975188	−2.14031407	2.76534906
H	0.01888167	−1.04122719	−0.79375932
C	0.03806153	−5.88266255	−1.11399635
H	−0.10483629	−6.29070427	1.57532529
F	0.70545294	6.99415194	−0.81951750
H	−0.02105562	3.19480781	−1.41171317
C	−0.03806153	5.88266255	−1.11399635
H	0.10483629	6.29070427	1.57532529
H	0.08975188	2.14031407	2.76534906
F	−0.70545294	−6.99415194	−0.81951750
F	−1.32967712	6.31816776	−1.31126526
H	0.13666425	4.54875664	3.35391455
H	−0.13666425	−4.54875664	3.35391455
F	1.32967712	−6.31816776	−1.31126526
F	−0.40223200	−5.41401579	−2.32586054

Local minimum conformer

E: −4873.44

H: −4719.50

G: −4761.99

N_{imag} = 0

N	0.01766460	1.15730684	1.29451410
C	0.00000000	0.00000000	0.51028436
H	0.05939637	1.04175103	2.30068133
C	0.02868485	2.50196845	0.86710663
N	−0.01766460	−1.15730684	1.29451410
O	0.00000000	0.00000000	−0.71649538
C	0.10102992	3.49258381	1.86581005
C	−0.03657502	2.88680169	−0.48219726
H	−0.05939637	−1.04175103	2.30068133
C	−0.02868485	−2.50196845	0.86710663
C	0.10816652	4.84293792	1.52500574
H	0.14993988	3.19902382	2.91377622
C	−0.03003286	4.24848396	−0.80268408
H	−0.09363264	2.13278586	−1.25448240
C	−0.10102992	−3.49258381	1.86581005
C	0.03657502	−2.88680169	−0.48219726
C	0.04414542	5.23584238	0.18549957
H	0.16250563	5.59384380	2.30910493
C	−0.04554718	4.65163109	−2.26088321
C	−0.10816652	−4.84293792	1.52500574
H	−0.14993988	−3.19902382	2.91377622
C	0.03003286	−4.24848396	−0.80268408
H	0.09363264	−2.13278586	−1.25448240
H	0.04582116	6.28644746	−0.08528292
F	−0.73498477	3.76793976	−3.04383241
F	−0.61151170	5.88586974	−2.45246258
F	1.22962895	4.72335623	−2.77656819

C	−0.04414542	−5.23584238	0.18549957
H	−0.16250563	−5.59384380	2.30910493
C	0.04554718	−4.65163109	−2.26088321
H	−0.04582116	−6.28644746	−0.08528292
F	0.73498477	−3.76793976	−3.04383241
F	0.61151170	−5.88586974	−2.45246258
F	−1.22962895	−4.72335623	−2.77656819

1,3-bis[3-(trifluoromethyl)phenyl]thiourea

Global minimum conformer

E: −4791.92

H: −4639.40

G: −4682.44

N_{imag} = 0

N	−1.05235146	0.38800767	0.21286455
N	1.11424986	−0.25328385	−0.03035825
C	0.24241359	0.55193196	0.69569145
C	2.48680287	−0.51270022	0.19690485
C	3.30656880	−0.68887851	−0.92728461
C	4.65751546	−1.00795081	−0.76734775
C	5.20979149	−1.13740177	0.51215591
C	4.38517252	−0.96360351	1.62673218
C	3.03060329	−0.66067550	1.48308411
C	−2.22400810	1.10811243	0.54702866
C	−3.43412271	0.40001844	0.57569130
C	−4.63231195	1.06975331	0.83709834
C	−4.63575129	2.44631016	1.09021490
C	−3.42587770	3.14454494	1.05701601
C	−2.22339108	2.49264394	0.78099668
F	−5.89221185	−0.84379428	0.13406212
H	2.89181026	−0.57703880	−1.92594841
S	0.67944694	1.55685306	1.94900895
H	−1.23245330	−0.50195957	−0.24413887
H	2.39945099	−0.53687315	2.35352676
H	0.80203634	−0.48797536	−0.96887492
C	5.53706586	−1.14590851	−1.98702525
H	6.26072707	−1.38035190	0.63234210
F	−7.00238292	1.02323761	0.50992169
H	−3.44176188	−0.67185063	0.39469435
C	−5.92096055	0.28620471	0.91215043
H	−5.56796565	2.96197597	1.29775893
H	−1.29324986	3.04516433	0.74835111
F	6.11891940	0.05271645	−2.33372754
F	4.83887929	−1.57063224	−3.08961522
F	6.56067335	−2.03396846	−1.79331250
F	−6.18855904	−0.13251946	2.19604567
H	−3.41838817	4.21639456	1.23800702
H	4.80005611	−1.07586923	2.62521000

Local minimum conformer

E: −4791.43

H: −4638.97

G: −4682.12

N_{imag} = 0

N	−0.34293673	1.08432017	−1.10752971
N	0.34293673	−1.08432017	−1.10752971
C	0.00000000	−0.00000000	−0.30381131
C	0.61951772	−2.41610833	−0.71644478
C	1.63423817	−3.10893038	−1.39425982
C	1.90765316	−4.44227406	−1.08222753
C	1.18131164	−5.09611507	−0.08653426

C	0.16289719	−4.40142677	0.58106607
C	−0.12777526	−3.07111448	0.27379454
C	−0.61951772	2.41610833	−0.71644478
C	−1.63423817	3.10893038	−1.39425982
C	−1.90765316	4.44227406	−1.08222753
C	−1.18131164	5.09611507	−0.08653426
C	−0.16289719	4.40142677	0.58106607
C	0.12777526	3.07111448	0.27379454
C	0.60653620	5.09999331	1.67914068
H	2.21543107	−2.59868767	−2.16058992
S	0.00000000	−0.00000000	1.35951249
H	−0.69572411	0.84214559	−2.02949583
H	−0.92167184	−2.54996263	0.79083550
H	0.69572411	−0.84214559	−2.02949583
F	−0.86629668	−6.41214006	1.36902848
H	1.39366982	−6.13055847	0.16474437
C	−0.60653620	−5.09999331	1.67914068
H	−2.21543107	2.59868767	−2.16058992
F	−0.09717151	5.11294048	2.86074280
H	−1.39366982	6.13055847	0.16474437
H	0.92167184	2.54996263	0.79083550
F	0.86629668	6.41214006	1.36902848
F	1.80953140	4.50543286	1.94293272
H	−2.69707577	4.96800586	−1.61305388
F	−1.80953140	−4.50543286	1.94293272
F	0.09717151	−5.11294048	2.86074280
H	2.69707577	−4.96800586	−1.61305388

1,3-bis[3-(trifluoromethyl)phenyl]selenourea

Global minimum conformer

E: −4770.22

H: −4618.08

G: −4661.54

N_{imag} = 0

N	0.04307034	1.13383157	0.10287065
N	−0.04307034	−1.13383157	0.10287065
C	−0.00000000	0.00000000	0.89381783
C	−0.26330441	−2.48222308	0.48024429
C	0.46520182	−3.47224754	−0.19209526
C	0.24373193	−4.82191437	0.09812817
C	−0.69031689	−5.19390507	1.07144733
C	−1.41535701	−4.19909346	1.73337168
C	−1.21718753	−2.84913895	1.44205479
C	0.26330441	2.48222308	0.48024429
C	−0.46520182	3.47224754	−0.19209526
C	−0.24373193	4.82191437	0.09812817
C	0.69031689	5.19390507	1.07144733
C	1.41535701	4.19909346	1.73337168
C	1.21718753	2.84913895	1.44205479
F	−1.46361978	5.48472753	−1.85439016
H	1.20466271	−3.19221728	−0.93798105
Se	−0.00000000	0.00000000	2.72675783
H	−0.34800639	1.02677762	−0.83025868
H	−1.79053713	−2.08475347	1.95108751
H	0.34800639	−1.02677762	−0.83025868
C	1.06947888	−5.87622705	−0.59931531
H	−0.85531469	−6.24253609	1.29768953
F	−0.39325481	7.05800880	−0.74123682
H	−1.20466271	3.19221728	−0.93798105
C	−1.06947888	5.87622705	−0.59931531
H	0.85531469	6.24253609	1.29768953
H	1.79053713	2.08475347	1.95108751
F	2.22132719	−6.16519100	0.09698607

F	1.46361978	−5.48472753	−1.85439016
F	0.39325481	−7.05800880	−0.74123682
F	−2.22132719	6.16519100	0.09698607
H	2.15444579	4.47847673	2.47986029
H	−2.15444579	−4.47847673	2.47986029

Local minimum conformer

E: −4769.87
H: −4618.39
G: −4659.94
N_{imag} = 0

N	−0.36617986	1.07527384	−1.09921521
N	0.36617986	−1.07527384	−1.09921521
C	0.00000000	0.00000000	−0.30846959
C	0.64474174	−2.40746676	−0.70248917
C	1.72129899	−3.07117116	−1.30664260
C	1.99906343	−4.40154165	−0.98122233
C	1.21366445	−5.07595940	−0.04670677
C	0.13150305	−4.40857215	0.54580910
C	−0.16230955	−3.08289968	0.22414635
C	−0.64474174	2.40746676	−0.70248917
C	−1.72129899	3.07117116	−1.30664260
C	−1.99906343	4.40154165	−0.98122233
C	−1.21366445	5.07595940	−0.04670677
C	−0.13150305	4.40857215	0.54580910
C	0.16230955	3.08289968	0.22414635
C	0.70474084	5.13409989	1.57486021
H	2.34801137	−2.54168079	−2.02206066
Se	0.00000000	0.00000000	1.52227600
H	−0.71325703	0.83633515	−2.02514650
H	−1.00445977	−2.57990586	0.67987972
H	0.71325703	−0.83633515	−2.02514650
F	−0.94306554	−6.43796941	1.21453744
H	1.42890822	−6.10734181	0.21506482
C	−0.70474084	−5.13409989	1.57486021
H	−2.34801137	2.54168079	−2.02206066
F	0.07685849	5.17909224	2.79742764
H	−1.42890822	6.10734181	0.21506482
H	1.00445977	2.57990586	0.67987972
F	0.94306554	6.43796941	1.21453744
F	1.92245539	4.54584119	1.77736505
H	−2.83777165	4.90721976	−1.45251029
F	−1.92245539	−4.54584119	1.77736505
F	−0.07685849	−5.17909224	2.79742764
H	2.83777165	−4.90721976	−1.45251029

1,3-bis[3,5-bis(trifluoromethyl)phenyl]urea

E: −5659.48
H: −5497.99
G: −5546.70
N_{imag} = 0

H	−2.82350257	−0.09103455	4.00078762
H	−4.91764024	−0.01792181	4.00079789
N	−5.02869591	−0.05799406	2.99385861
C	−3.87057700	−0.05443162	2.21039922
H	−7.08384551	0.01615454	4.60111851
N	−2.71245142	−0.05092517	2.99384935
H	−1.75407039	0.06778769	0.43728135
O	−3.87058573	−0.05439005	0.98588356
C	−1.37264133	−0.03508765	2.55714239
C	−0.37763326	−0.07933045	3.55158864
C	0.96928060	−0.05501425	3.19958259

C	1.35833484	0.00882261	1.85643755
C	0.36471005	0.05326194	0.87861050
C	−0.99536273	0.03091896	1.20684266
H	−0.65730748	−0.12500428	4.60111154
C	2.02883209	−0.15243383	4.27524897
H	2.40689842	0.03254174	1.58421242
C	0.74021692	0.09546940	−0.58931141
C	−6.36850629	−0.07378239	2.55715217
C	−6.74577969	−0.13977682	1.20685047
C	−8.10585113	−0.16208237	0.87861215
C	−9.09947909	−0.11761156	1.85643518
C	−8.71042985	−0.05379557	3.19958244
C	−7.36351661	−0.02951358	3.55159450
H	−5.98706928	−0.17665949	0.43729241
C	−8.48135725	−0.20427220	−0.58931047
H	−10.14804208	−0.14130670	1.58420545
C	−9.76998261	0.04364244	4.27524590
F	−8.23197040	0.99907815	−1.20246027
F	−9.80521896	−0.48331499	−0.78223902
F	−7.76399291	−1.14991243	−1.27180673
F	2.06409064	0.37445564	−0.78223658
F	0.49077093	−1.10785260	−1.20249237
F	0.02289499	1.04116157	−1.27178130
F	2.46342571	−1.44668108	4.43504354
F	1.56918004	0.26115444	5.49837360
F	3.13140939	0.59993293	3.98113082
F	−10.20452704	1.33790309	4.43506451
F	−9.31035161	−0.36998760	5.49836456
F	−10.87258748	−0.70867597	3.98110781

1,3-bis[3,5-bis(trifluoromethyl)phenyl]thiourea

E: −5577.25
H: −5417.16
G: −5466.43
N_{imag} = 0

N	0.93847736	0.78623557	0.05366854
N	−1.14599596	−0.12550294	0.09297390
C	−0.42555439	1.06314000	−0.00589803
C	−2.53879263	−0.31277194	−0.05778750
C	−3.17615049	−1.23352604	0.78296427
C	−4.54334581	−1.48664499	0.63619816
C	−5.28982693	−0.82136343	−0.33717068
C	−4.64079930	0.09169879	−1.17676936
C	−3.27549708	0.34859792	−1.05272510
C	2.01201115	1.70322740	0.11842792
C	3.18343520	1.39702417	−0.58524038
C	4.28862461	2.25013870	−0.51158425
C	4.23697410	3.41743513	0.25121697
C	3.06333097	3.71001123	0.95613408
C	1.95471165	2.86517518	0.90388094
C	2.98887669	4.99199051	1.75936092
H	−2.61223695	−1.74287442	1.56025710
S	−1.09147122	2.57615942	−0.16983756
H	1.20520230	−0.14515707	−0.25446064
H	−2.78743498	1.04689369	−1.71857079
H	−0.67530696	−0.87844747	0.58803689
C	−5.19749390	−2.52827687	1.51657194
H	−6.35141741	−1.01270196	−0.44481742
C	−5.45016235	0.84160305	−2.21439981
H	3.23097228	0.50261579	−1.20124730
C	5.56158107	1.86817614	−1.23393642
H	5.09176922	4.08227503	0.30129666
H	1.06091357	3.09993765	1.46518678

F	-5.02149925	-3.79462249	1.00711358
F	-6.54248353	-2.33508040	1.64221376
F	-4.66633005	-2.54053099	2.78038235
F	-4.68029111	1.28975406	-3.25104498
F	-6.06571313	1.94097452	-1.66880645
F	-6.44021201	0.06192062	-2.75111818
F	4.18587847	5.29186933	2.35412503
F	2.04805439	4.93326704	2.74884305
F	2.66534314	6.06225169	0.96238032
F	6.35318879	2.94672830	-1.50222565
F	5.30744865	1.24074962	-2.42618880
F	6.31702221	0.99382358	-0.48634128

1,3-bis[3,5-bis(trifluoromethyl)phenyl]selenourea

E: −5555.68
H: −5396.00
G: −5446.09
N_{imag} = 0

H	-2.87990544	-0.53380834	3.87180882
H	-4.86120763	0.42483957	3.87193338
N	-5.00376561	0.03385273	2.94342931
C	-3.87060195	-0.05438863	2.15253613
H	-6.91408462	1.60162165	3.85545389
N	-2.73738607	-0.14274743	2.94332983
H	-1.62397666	1.53951365	1.14365294
Se	-3.87069233	-0.05427999	0.32343948
C	-1.38298980	-0.09585568	2.53362231
C	-0.47776281	-0.97774362	3.13243633
C	0.87686436	-0.93178472	2.78382603
C	1.33557131	-0.01893441	1.83425997
C	0.42047579	0.86520118	1.24862005
C	-0.93074875	0.84078372	1.59194071
H	-0.82701660	-1.71034417	3.85545097
C	1.84577035	-1.85810201	3.48410993
H	2.38383718	0.01029557	1.55929775
C	0.90840663	1.83837985	0.19621038
C	-6.35817729	-0.01298364	2.53374037
C	-6.81044796	-0.94967293	1.59213335
C	-8.16167527	-0.97406520	1.24880038
C	-9.07673344	-0.08985688	1.83437729
C	-8.61799494	0.82304247	2.78388854
C	-7.26336659	0.86898592	3.13249060
H	-6.11723902	-1.64846368	1.14390459
C	-8.64954057	-1.94728609	0.19640065
H	-10.12499944	-0.11907146	1.55941787
C	-9.58690478	1.74939009	3.48412668
F	-8.73453355	-1.34932770	-1.03647884
F	-9.89882171	-2.42905382	0.48686551
F	-7.82076492	-3.02552815	0.06016881
F	2.15715830	2.32110344	0.48741826
F	0.99473036	1.24002059	-1.03638144
F	0.07901090	2.91600295	0.05897674
F	1.29803990	-3.09361911	3.71547595
F	2.21419029	-1.35921971	4.71300950
F	2.99419014	-2.04860295	2.77271416
F	-9.03902438	2.98476331	3.71588150
F	-9.95572172	1.25031356	4.71282965
F	-10.73511919	1.94023128	2.77248472

1-phenyl-3-[3,5-bis(trifluoromethyl)phenyl]urea

E: −4872.83
H: −4718.98
G: −4761.65

N_{imag} = 0

N	0.94878088	0.77512379	0.19856156
N	−1.14902472	−0.18028393	−0.01199382
C	−0.40696429	1.01428914	0.01098595
C	−2.53572352	−0.33436877	−0.17798355
C	−3.03784690	−1.64976214	−0.19570540
C	−4.40189401	−1.88195216	−0.35664437
C	−5.29897856	−0.81784938	−0.50011633
C	−4.79285436	0.48297647	−0.47873670
C	−3.42788876	0.74231341	−0.32135303
C	2.00746372	1.71366701	0.27604667
C	3.29975120	1.19580362	0.48515626
C	4.39797314	2.05006107	0.57296393
C	4.22542349	3.43382015	0.45357127
C	2.94090967	3.94464878	0.24583287
C	1.82847088	3.10243010	0.15529375
H	2.79259230	5.01810068	0.15135709
H	−2.35925524	−2.49236533	−0.08841498
O	−0.90930265	2.12488258	−0.11950238
H	1.24469345	−0.18868986	0.30524328
H	−3.04973277	1.75509677	−0.30751380
H	−0.64004124	−1.05126048	0.08742821
C	−4.92764141	−3.29928927	−0.31698005
H	−6.35970428	−1.00014021	−0.62535336
C	−5.72741750	1.65793885	−0.68181535
H	3.44161242	0.11931903	0.57910028
H	5.38871210	1.63183680	0.73488476
H	5.08020778	4.10182359	0.52179176
H	0.83716791	3.50387481	−0.00481818
F	−3.98562847	−4.21594455	−0.70763039
F	−6.01704847	−3.47016615	−1.12495454
F	−5.31787412	−3.65636969	0.95303754
F	−5.40229080	2.71984814	0.11698306
F	−7.03150527	1.33813439	−0.41842295
F	−5.68507383	2.11213121	−1.97909383

1-phenyl-3-[3,5-bis(trifluoromethyl)phenyl]thiourea

E: −4790.71
H: −4637.65
G: −4682.92
N_{imag} = 0

C	3.20572336	1.10229703	−1.34744942
C	4.06896578	2.14473195	−1.68860579
C	3.69166459	3.47208689	−1.45959125
C	2.44918877	3.74207593	−0.87544096
C	1.58065457	2.70716291	−0.52149827
C	−1.84718177	−1.68091682	0.27916223
C	−2.20440934	−2.35446644	1.45438732
C	−3.44017886	−3.00277978	1.54540775
C	−4.33742898	−2.97914801	0.47702226
C	−3.96832314	−2.30884260	−0.69572922
C	−2.73531212	−1.66703043	−0.80925695
C	−4.94772239	−2.25015611	−1.84848697
H	3.49525335	0.06978740	−1.53874672
S	−1.31796855	1.23400780	−0.98922810
H	0.08129914	−1.41947165	0.91459114
H	0.62154210	2.92447665	−0.06936373
H	1.66960816	−0.60439991	−0.32464996
H	5.03168930	1.91729318	−2.14010894
H	4.35894685	4.28613783	−1.73126790
H	2.14952351	4.77017363	−0.68578354
H	−1.52621732	−2.36222956	2.30395938

C	−3.77443460	−3.77078360	2.80375765
H	−5.29821862	−3.47531063	0.55131299
H	−2.46052392	−1.16641422	−1.72739103
N	−0.57090998	−1.08593653	0.20994897
N	1.15244414	0.26805277	−0.39520235
C	−0.22300053	0.13408293	−0.38860035
C	1.95642836	1.37781509	−0.76665721
F	−5.84666027	−1.22313861	−1.69185591
F	−5.68345317	−3.40172811	−1.95521736
F	−4.32772530	−2.05440368	−3.05124882
F	−3.30158075	−3.14637602	3.92992777
F	−3.20953303	−5.02673143	2.79059755
F	−5.11827716	−3.94432204	2.97050672

1-phenyl-3-[3,5-bis(trifluoromethyl)phenyl]selenourea

E: −4769.12
H: −4617.05
G: −4661.07
N_{imag} = 0

N	−0.93421772	−0.94004236	−0.07701141
N	−0.78082112	1.31861242	0.10613567
C	−0.06843061	0.14802992	−0.00802232
C	−0.32411876	2.66443785	0.04105116
C	−0.90016956	3.59237521	0.92169365
C	−0.52778612	4.93751847	0.86463218
C	0.42583773	5.36228857	−0.06587011
C	0.99258807	4.43321560	−0.94634731
C	0.62015274	3.08838279	−0.90458766
C	−0.61199681	−2.31416051	−0.02833505
C	−1.29706689	−3.18916790	−0.87808080
C	−1.03703584	−4.56385244	−0.82733729
C	−0.08866173	−5.07568841	0.05758825
C	0.58455482	−4.19106901	0.91061148
C	0.32993797	−2.82057502	0.88212386
C	1.63447039	−4.73825745	1.85299894
H	−1.62978317	3.25713654	1.65725171
Se	1.75809230	0.01498651	−0.06584920
H	−1.84881244	−0.74145388	−0.47557894
H	1.05764596	2.37254622	−1.58967007
H	−1.72175873	1.22099676	0.48102364
H	−0.97724082	5.64829524	1.55390639
H	0.72283178	6.40728072	−0.10676708
H	1.72812422	4.75653591	−1.67917990
H	−2.02022464	−2.80074022	−1.59077670
C	−1.83101282	−5.48931451	−1.72005693
H	0.11803219	−6.13924712	0.09048354
H	0.84548306	−2.15128760	1.55707853
F	−3.09013540	−5.71992718	−1.21125206
F	−1.23754121	−6.70852497	−1.87434154
F	2.83674917	−4.92118849	1.21399316
F	1.27527106	−5.95814248	2.36633285
F	1.87017522	−3.90789349	2.91281758
F	−2.01296275	−4.96292502	−2.97414891

1-cyclohexyl-3-[3,5-bis(trifluoromethyl)phenyl]urea

E: −5385.07
H: −5188.34
G: −5230.67
N_{imag} = 0

H	2.79301723	4.02400729	3.27992051
H	2.52200984	4.48625847	1.60129327
H	4.60575635	2.46075499	2.59993117

H	4.94729079	4.06999966	1.96314419
N	0.95722491	0.78157240	0.47002487
N	−1.14369096	−0.18325633	0.23365146
C	−0.38435422	1.00243669	0.22459701
C	−2.52410119	−0.32931325	0.03305828
C	−3.03876602	−1.63966958	−0.00059372
C	−4.40253833	−1.85829245	−0.18510092
C	−5.28783751	−0.78580701	−0.33699405
C	−4.76955035	0.51050641	−0.30042777
C	−3.40531836	0.75610888	−0.12035935
C	−5.69061648	1.69355520	−0.51404044
F	−4.00825465	−4.19093714	−0.56947612
F	−5.64058173	2.14030470	−1.81391513
F	−5.35868923	2.75782370	0.27941409
F	−6.99967073	1.38906574	−0.25329115
F	−6.03698308	−3.42431167	−0.96105617
F	−5.32290966	−3.64305076	1.10881148
H	−2.36949211	−2.48875786	0.11384650
O	−0.86004112	2.11158928	−0.00856849
H	1.23315663	−0.09505015	0.90156192
H	−3.01489539	1.76449443	−0.10158670
H	−0.63010732	−1.05420692	0.30945727
C	−4.93961613	−3.27111899	−0.15999093
H	−6.34791455	−0.95770699	−0.48045292
C	1.88676232	1.90943849	0.64846707
C	4.31685104	2.62956628	0.45744032
C	3.31459876	1.47170076	0.27925109
C	1.82881208	2.49006692	2.07749953
C	2.82772843	3.65086805	2.24868758
C	4.25981047	3.21860798	1.88024404
H	1.55299055	2.68206155	−0.05354284
H	4.08259607	3.42003906	−0.27103170
H	5.33122337	2.28021498	0.22729381
H	3.61198929	0.63282015	0.92990970
H	3.32915584	1.10057604	−0.75301203
H	2.07067446	1.68797072	2.79272088
H	0.80607947	2.82190489	2.28900910

1-cyclohexyl-3-[3,5-bis(trifluoromethyl)phenyl]thiourea

E: −5305.03
H: −5109.64
G: −5152.67
N_{imag} = 0

H	3.28519007	3.18398971	3.78743646
H	2.68294425	4.18500362	2.46808693
H	4.89043598	2.05729855	2.25640279
H	5.12418121	3.80367035	2.17057879
N	0.85523317	1.03425692	0.44915201
N	−1.15175394	−0.03056140	0.18316524
C	−0.50067087	1.17770528	0.46050713
C	−2.53019165	−0.26562597	0.01461241
C	−3.05640911	−1.47624561	0.48653488
C	−4.40847409	−1.77823263	0.29863768
C	−5.25630608	−0.87828588	−0.34862507
C	−4.72030710	0.32506110	−0.82292738
C	−3.37110931	0.63850554	−0.65666271
C	−5.63813106	1.32145589	−1.49781802
F	−4.67417053	−4.10807392	−0.15353528
F	−4.96765079	2.16520487	−2.33882403
F	−6.28818178	2.10933573	−0.57841304
F	−6.61178942	0.70199913	−2.23876676
F	−6.28354108	−3.10882230	0.96821889
F	−4.34874215	−3.51813785	1.94125390

H	-2.41498899	-2.17795821	1.01393816
S	-1.28212589	2.62233831	0.79233047
H	1.24522860	0.14762672	0.14219611
H	-2.97094059	1.56692291	-1.03937948
H	-0.61546269	-0.86849054	0.39053658
C	-4.93303378	-3.11593182	0.76652651
H	-6.30620392	-1.10928555	-0.48686476
C	1.82142306	2.07929229	0.81843231
C	4.17243723	2.94437720	0.41245072
C	3.13154970	1.86945579	0.04031494
C	2.06771866	2.11251816	2.34085913
C	3.10051081	3.19543646	2.70605588
C	4.41839819	2.99770181	1.93283814
H	1.36840318	3.03221317	0.51941113
H	3.81046539	3.92468551	0.06930928
H	5.11081301	2.75012093	-0.12182038
H	3.53916039	0.87340595	0.28012005
H	2.92774660	1.88801313	-1.03805373
H	2.43562193	1.12535873	2.66001472
H	1.11548939	2.29200874	2.85175755

1-cyclohexyl-3-[3,5-bis(trifluoromethyl)phenyl]selenourea

E: –5284.08
H: –5089.04
G: –5132.76
N_{imag} = 0

H	3.33332587	3.11201664	3.81674143
H	2.68134005	4.14729122	2.54827054
H	4.92291348	2.07183540	2.20985010
H	5.12207718	3.82433600	2.18184513
N	0.86328861	1.04102158	0.45961523
N	-1.12988826	-0.04468936	0.19816693
C	-0.48791444	1.15507320	0.48268555
C	-2.50996900	-0.28388986	0.02409942
C	-3.04854183	-1.46791491	0.54117743
C	-4.40336278	-1.76447456	0.35343288
C	-5.23486872	-0.88347685	-0.33815077
C	-4.68331389	0.29348789	-0.86031406
C	-3.33260253	0.59947161	-0.69426322
C	-5.58545032	1.26585919	-1.58827162
F	-4.65743566	-4.11503675	0.01736779
F	-4.89572686	2.08417476	-2.43857665
F	-6.26002764	2.08011454	-0.71082558
F	-6.53944154	0.62027529	-2.33324853
F	-6.30054052	-3.05839951	1.03306027
F	-4.39750030	-3.41392366	2.08674633
H	-2.41833836	-2.15066209	1.10549053
Se	-1.36575627	2.72712814	0.89004922
H	1.26684883	0.15767210	0.15619378
H	-2.91637057	1.50408930	-1.11559303
H	-0.58552467	-0.88606504	0.37244613
C	-4.94464203	-3.07517521	0.87457279
H	-6.28602358	-1.10951933	-0.47581127
C	1.82271506	2.08817761	0.84446051
C	4.14578136	3.01015106	0.41585829
C	3.11646699	1.92925714	0.02854573
C	2.10210620	2.06986068	2.36075679
C	3.12323691	3.15853686	2.74098309
C	4.42602022	3.01384848	1.93124170
H	1.34381120	3.04155851	0.59066203
H	3.75739241	3.99468651	0.11669625
H	5.07478888	2.85339389	-0.14628734
H	3.54779677	0.93305730	0.22215893

H	2.88630355	1.98299475	−1.04323220
H	2.49478640	1.07916595	2.63688954
H	1.15793139	2.21448173	2.89718343

5,6-dihydroindolo[2,3-*b*]indole (X = C; R = 3-CF_3-phenyl)

E: −4738.32
H: −4597.40
G: −4637.56
N_{imag} = 0

C	0.00000000	−0.00000000	−0.07955100
C	−0.02751736	−1.38155841	0.32591486
C	0.02751736	1.38155841	0.32591486
C	−0.00000000	−0.00000000	−1.48034639
C	−0.03888366	−2.06177578	1.55456623
C	−0.04200278	−2.15965596	−0.88505787
C	0.03888366	2.06177578	1.55456623
C	0.04200278	2.15965596	−0.88505787
N	0.02246621	1.27641814	−1.98830637
N	−0.02246621	−1.27641814	−1.98830637
C	−0.06342945	−3.45488796	1.56635814
H	−0.02645621	−1.50894141	2.48994659
C	−0.06652871	−3.54786224	−0.88190300
C	0.06342945	3.45488796	1.56635814
H	0.02645621	1.50894141	2.48994659
C	0.06652871	3.54786224	−0.88190300
H	0.03585183	1.55394994	−2.96000200
H	−0.03585183	−1.55394994	−2.96000200
C	−0.07425440	−4.19153230	0.36373189
H	−0.06887410	−3.98638234	2.51328339
H	−0.07397931	−4.12205678	−1.80343861
C	0.07425440	4.19153230	0.36373189
H	0.06887410	3.98638234	2.51328339
H	0.07397931	4.12205678	−1.80343861
C	−0.14882287	−5.69287512	0.42915474
C	0.14882287	5.69287512	0.42915474
F	0.66776925	−6.21575918	1.40144199
F	0.20380809	−6.29360680	−0.75358080
F	−1.42238903	−6.13764190	0.72329386
F	−0.66776925	6.21575918	1.40144199
F	−0.20380809	6.29360680	−0.75358080
F	1.42238903	6.13764190	0.72329386

5,6-dihydroindolo[2,3-*b*]indole (X = Si; R = 3-CF_3-phenyl)

E: −4617.19
H: −4479.29
G: −4520.44
N_{imag} = 0

Si	−0.05579678	0.92510223	−1.18028164
C	0.35358305	−0.87767080	−0.93475243
C	0.07453290	1.01782643	0.72883939
C	−1.48968098	0.25552965	−2.26013252
N	−0.19545604	−1.72541750	−1.83863221
N	0.90474110	−1.18926950	0.26369736
C	−0.09180429	2.08003091	1.63279055
C	0.79332917	−0.12816356	1.20456894
C	−2.50657746	0.90322601	−2.98090347
C	−1.20825336	−1.10363668	−2.62043546
H	−0.09506952	−2.73726197	−1.83398135
H	1.16216571	−2.12458662	0.56838760
C	0.37994346	2.00078138	2.94372839
C	1.58059361	0.78779814	4.79179044
C	1.32491897	−0.19840754	2.49152573

C	-3.23680820	0.23807273	-3.96668388
C	-3.72933553	-1.80028404	-5.35635782
C	-1.88588331	-1.76323912	-3.64487459
C	1.09784189	0.86898425	3.36630423
H	0.20711085	2.81850216	3.63732922
F	2.05591591	1.98897610	5.25081679
F	2.58535507	-0.13128554	4.95835754
F	0.56981877	0.41821980	5.65446534
H	1.89527563	-1.06454554	2.81636579
C	-2.91685701	-1.08763814	-4.30579790
H	-4.04433882	0.74647889	-4.48536840
F	-4.04778373	-0.98509270	-6.41155533
F	-3.08155096	-2.89286149	-5.87473249
F	-4.92672398	-2.26264492	-4.85144468
H	-1.62974838	-2.78251470	-3.92144704
H	-2.74085848	1.94306752	-2.76295133
H	-0.60771970	2.98258923	1.31204331

5,6-dihydroindolo[2,3-*b*]indole (X = Ge; 3-CF_3-phenyl)

E: −4587.14
H: −4449.76
G: −4491.98
N_{imag} = 0

Ge	0.88365101	1.42536351	-0.00000000
C	1.19094448	-0.56592563	-0.00000000
C	0.03463634	0.88073924	1.79337044
C	0.03463634	0.88073924	-1.79337044
N	1.15064865	-1.17306491	-1.19771632
N	1.15064865	-1.17306491	1.19771632
C	-0.57328665	1.68213558	2.76693305
C	0.55874242	-0.37062960	2.22434508
C	-0.57328665	1.68213558	-2.76693305
C	0.55874242	-0.37062960	-2.22434508
H	1.20626763	-2.18266066	-1.32997925
H	1.20626763	-2.18266066	1.32997925
C	-0.71962349	1.24136904	4.08693195
H	-0.95936696	2.66266574	2.49497045
C	0.47548997	-0.80789496	3.54503563
C	-0.71962349	1.24136904	-4.08693195
H	-0.95936696	2.66266574	-2.49497045
C	0.47548997	-0.80789496	-3.54503563
C	-0.18610624	0.00400095	4.47498838
H	-1.23551039	1.85879215	4.81648324
H	0.91088925	-1.75496768	3.85361483
C	-0.18610624	0.00400095	-4.47498838
H	-1.23551039	1.85879215	-4.81648324
H	0.91088925	-1.75496768	-3.85361483
C	-0.37041717	-0.48579765	5.88883190
C	-0.37041717	-0.48579765	-5.88883190
F	-0.39573246	0.53594666	6.79967627
F	0.62366385	-1.34846409	6.27915165
F	-1.55759249	-1.17188875	6.04146528
F	-0.39573246	0.53594666	-6.79967627
F	0.62366385	-1.34846409	-6.27915165
F	-1.55759249	-1.17188875	-6.04146528

1,3-bis[4-(trifluoromethyl)phenyl]urea

E: −4875.34
H: −4722.00
G: −4762.80
N_{imag} = 0

C	0.06319095	-8.41952394	0.82605314
C	0.07750647	-8.81355901	-0.52448146
C	0.05724666	-9.40701837	1.83193078
N	0.05759936	-7.07599571	1.25009783
C	0.08457797	-10.17099274	-0.84414501
H	0.08422109	-8.06252826	-1.30214477
C	0.06463906	-10.75705567	1.50205985
H	0.04905076	-9.11132823	2.88016332
C	0.05512759	-5.91864109	0.46493062
H	0.04907232	-6.95892232	2.25717363
C	0.08142818	-11.14921848	0.15657761
H	0.09885138	-10.46741547	-1.88877466
H	0.06378049	-11.50536538	2.28887011
N	0.05270534	-4.76128890	1.25010164
O	0.05508915	-5.91863945	-0.76021634
C	0.03648224	-12.61057646	-0.20173425
C	0.04708177	-3.41775908	0.82606224
H	0.06129701	-4.87836572	2.25717649
F	-1.25764355	-13.08505279	-0.26921026
F	0.68288719	-13.39405877	0.72171047
F	0.60646159	-12.87074739	-1.41989997
C	0.03267459	-3.02371825	-0.52446957
C	0.05308884	-2.43026876	1.83194382
C	0.02557565	-1.66628297	-0.84412696
H	0.02591162	-3.77474558	-1.30213573
C	0.04566788	-1.08023033	1.50207913
H	0.06135560	-2.72596342	2.88017455
C	0.02878672	-0.68806173	0.15659949
H	0.01123129	-1.36985583	-1.88875429
H	0.04657463	-0.33192395	2.28889262
C	0.07369993	0.77329911	-0.20170479
F	-0.49631187	1.03346906	-1.41985498
F	1.36781619	1.24779778	-0.26920619
F	-0.57269683	1.55676446	0.72176078

Data S7. Cartesian coordinates and energies of optimized hydrogen-bonded complexes

Below is a list consisting of the Cartesian coordinates (in Å), ADF total bond energies *E*, enthalpies *H*, Gibbs free energies *G* (in kcal mol^{-1}), and the number of imaginary frequencies (N_{imag}) of all optimized hydrogen-bonded complexes reported in this work, optimized at the ZORA-BLYP-D3(BJ)/TZ2P level. It is specified if C_{2v} symmetry is enforced during the optimization process.

Formaldehyde–Urea

E: −1559.34
H: −1497.47
G: −1526.53
N_{imag} = 0

```
N       1.15351322      -0.46839336      -0.26503547
N      -1.11817925      -0.45487371       0.28420239
C       0.02345509       0.29150225       0.01513160
C      -0.01607548      -4.58541910      -0.03744995
O       0.03297064       1.52266954       0.02409967
O      -0.00672882      -3.37089887      -0.01746189
H       2.02675962       0.04007522      -0.20852825
H      -1.98347145       0.06780697       0.23573500
H       1.17200607      -1.45268468      -0.01961169
H      -1.15188645      -1.43512230       0.02463312
H       0.91469765      -5.17859775       0.09080988
H      -0.95583087      -5.15967113      -0.18493820
```

Formaldehyde–Thiourea

E: −1481.96
H: −1421.26
G: −1451.64
N_{imag} = 0

```
N       1.15772326      -0.45957692      -0.16373446
N      -1.12307039      -0.44644661       0.17045731
C       0.02337417       0.27975018       0.01617927
C      -0.01570226      -4.54440136      -0.03253933
S       0.03733297       1.96458766       0.04532135
O      -0.00622696      -3.32843844      -0.03157409
H       2.03428966       0.04094910      -0.18879618
H      -1.99126107       0.06716077       0.21457674
H       1.15630215      -1.47085856      -0.07861506
H      -1.13802496      -1.45443015       0.05371417
H       0.92507071      -5.13397724      -0.01748645
H      -0.96557504      -5.11919043      -0.04854847
```

Formaldehyde–Selenourea

E: −1461.19
H: −1400.77
G: −1431.77
N_{imag} = 0

```
N       1.16464857      -0.46852983      -0.09231803
N      -1.13005006      -0.45253332       0.10541604
C       0.02289441       0.25660871       0.01352752
C      -0.01540095      -4.53117682      -0.03391233
Se      0.03733358       2.11052964       0.03153876
O      -0.00602287      -3.31424834      -0.02229106
H       2.03575099       0.03697806      -0.16322662
H      -1.99317705       0.06507003       0.18594170
```

H	1.16116448	−1.48408686	−0.10403711
H	−1.14245493	−1.46805454	0.09669327
H	0.92177961	−5.11917779	−0.12329958
H	−0.96155915	−5.10625402	0.04433119

Formaldehyde–1-cyclohexylurea

E: −3583.20
H: −3426.89
G: −3461.73
N_{imag} = 0

N	3.27482333	−0.24713294	−5.96070818
N	1.00213148	0.08412114	−6.38481949
C	2.18080458	0.58327307	−5.81938038
C	2.27266307	−3.87998088	−7.73137135
H	5.71934466	−0.75223811	−2.01719951
O	2.13733361	−2.69017478	−7.52843861
H	1.58186334	−4.44021423	−8.39727135
O	2.21611645	1.66759189	−5.23007485
H	5.92020322	0.76336012	−2.89424330
H	6.88464303	−1.99997021	−3.82802307
H	3.09314885	−4.46401142	−7.26113074
H	0.29535906	0.79496973	−6.53282633
H	3.11756372	−1.18297723	−6.32478091
H	1.06982199	−0.62769185	−7.10549715
H	7.91247784	−0.68022269	−3.26596604
C	4.51378207	−0.00508877	−5.21880574
C	7.03569934	−0.35560261	−5.23339538
C	5.71216977	−0.58953550	−5.98802083
C	4.45374552	−0.56272365	−3.77886552
C	5.77496628	−0.31961921	−3.02462236
C	6.97589814	−0.90299062	−3.79387071
H	4.62252362	1.08449405	−5.15784701
H	7.24093968	0.72491915	−5.19925534
H	7.86658399	−0.81624176	−5.78371423
H	5.55745346	−1.67323427	−6.12059326
H	5.75293889	−0.14639874	−6.99098823
H	4.25148377	−1.64449965	−3.82988197
H	3.61235038	−0.09724504	−3.25312635

Formaldehyde–1-cyclohexylthiourea

E: −3506.80
H: −3351.02
G: −3388.31
N_{imag} = 0

H	0.66238050	−0.37001684	1.34224472
H	−1.35148484	−0.74811382	0.51622071
N	−1.20588488	−0.01474794	−0.17137512
C	0.06125029	0.46180403	−0.42409573
S	0.38553010	1.38076091	−1.80468772
N	0.98278057	0.14619259	0.52557667
C	2.47131995	2.01006121	1.24543394
C	3.93128694	2.49912130	1.28266667
C	4.85441343	1.46219604	1.95125242
C	4.73703480	0.08622015	1.26680895
C	3.27617354	−0.40369011	1.21798475
H	5.89657971	1.80654858	1.92754725
H	2.65882063	0.76204974	−0.50103213
H	1.83195579	2.72451457	0.71555084
H	2.08478157	1.91596838	2.27236770
H	−1.85849634	0.05138160	−0.94101765
H	4.57756800	1.36274167	3.01249172
H	3.20523218	−1.35606148	0.67700096

C	2.36251623	0.63765668	0.54766722
H	2.92076799	−0.58608278	2.24596030
H	4.27476746	2.68104385	0.25339470
H	3.99191052	3.46048364	1.80877650
H	5.36158289	−0.65374712	1.78408761
H	5.12189593	0.16082607	0.23894538
H	0.44638395	−2.46780698	4.02356338
O	−0.56240523	−1.73911754	2.41699891
H	−1.24694847	−3.21416023	3.63384333
C	−0.45265356	−2.48394388	3.37135742

Formaldehyde–1-cyclohexylselenourea

E: −3486.50
H: −3331.04
G: −3369.27
N_{imag} = 0

N	3.22209046	−0.27532978	−5.83276676
N	1.03309910	0.08212880	−6.44011295
C	2.15895956	0.56025573	−5.83348804
C	2.33284166	−3.79616462	−7.87356063
H	5.80028545	−0.62000488	−1.97292194
O	2.14590732	−2.67837613	−7.43135050
H	1.59963130	−4.28420256	−8.54922572
Se	2.15363898	2.25604572	−5.06643743
H	5.99486244	0.84143556	−2.93905977
H	6.87287102	−1.98881203	−3.75350775
H	3.24456583	−4.37763227	−7.62126580
H	0.26663379	0.73102458	−6.55314062
H	3.09765652	−1.19945014	−6.24382862
H	1.04827394	−0.77097922	−6.99149135
H	7.94588940	−0.66202735	−3.30400443
C	4.49826906	−0.01859275	−5.15730982
C	7.00261408	−0.42677200	−5.25210862
C	5.64709673	−0.67270682	−5.94414319
C	4.47304061	−0.50021654	−3.69210011
C	5.82654716	−0.24379492	−3.00362493
C	6.98586024	−0.89374950	−3.78338488
H	4.62643489	1.07087410	−5.15861277
H	7.22905875	0.64914431	−5.28591126
H	7.80195347	−0.93483644	−5.80700485
H	5.46635308	−1.75845185	−6.01256657
H	5.65934605	−0.28100786	−6.96929198
H	4.24602308	−1.57782222	−3.67607883
H	3.66239693	0.01773289	−3.16773697

Formaldehyde–1-phenylurea

Global minimum
E: −3073.92
H: −2959.79
G: −2995.21
N_{imag} = 0

N	−0.98946711	0.03556856	0.53508894
N	−3.27370354	−0.34877490	0.54483561
C	−2.24647496	0.52538037	0.18738715
C	−0.61353805	−2.99992311	−0.68792553
O	−2.45939827	1.61769026	−0.33493422
O	−1.24287763	−2.98369736	0.35447337
H	−0.41516285	−3.95017121	−1.22698175
H	−4.17300308	−0.10887960	0.14525651
H	−0.97752976	−0.89009652	0.95531548
H	−3.07646865	−1.33987396	0.64133325

H	-0.21133362	-2.06998567	-1.14126449
H	-0.39271575	2.40341245	-0.68066399
C	0.27698701	0.59542113	0.28122147
C	1.40171428	-0.14573803	0.70022815
C	2.69194562	0.33192331	0.47009510
C	2.88654271	1.55623655	-0.17957914
C	1.76983197	2.29280237	-0.58909832
C	0.46986627	1.83007269	-0.36678997
H	1.25690876	-1.09185550	1.22015732
H	3.54576158	-0.25332777	0.80489171
H	3.89125614	1.93123779	-0.35786650
H	1.90626480	3.24886045	-1.09041854

Local minimum
E: −3073.52
H: −2960.01
G: −2994.31
N_{imag} = 0

N	-0.97041216	0.09619172	0.12687970
N	-3.25826861	-0.25778940	0.27610916
C	-2.23471989	0.67951603	0.13404334
C	-0.63500407	-3.83553764	-0.08988807
O	-2.45695417	1.88745561	0.06720745
O	-1.42916602	-2.91852977	-0.01233618
H	-0.96947336	-4.88049904	-0.25783244
H	-4.17976135	0.10623133	0.06746270
H	-0.94600859	-0.91966110	0.18020973
H	-3.09419817	-1.23206819	0.04657623
H	0.45970547	-3.67001420	0.00343008
H	-0.41050618	2.75976788	-0.06341879
C	0.28558423	0.72414690	0.04366493
C	1.42373220	-0.10887885	0.05941541
C	2.70560063	0.43368641	-0.02120020
C	2.88085639	1.81909764	-0.11868306
C	1.75235562	2.64555783	-0.13293085
C	0.46063667	2.11781975	-0.05313874
H	1.29489742	-1.18773636	0.13893176
H	3.56905383	-0.22821853	-0.00630934
H	3.87921306	2.24495302	-0.18109108
H	1.87283706	3.72450898	-0.20710173

Formaldehyde–1-phenylthiourea

E: −2994.31
H: −2881.33
G: −2917.71
N_{imag} = 0

N	-1.01128868	0.10055562	0.52661417
N	-3.22648151	-0.44356180	0.59205885
C	-2.28652408	0.47919240	0.17446365
C	-0.63612808	-2.88697388	-0.75010821
S	-2.76280592	1.88132561	-0.61674107
O	-1.17688789	-2.92594249	0.34071381
H	-0.40076613	-3.81615865	-1.30989554
H	-4.16623336	-0.27849837	0.25420650
H	-0.96570787	-0.79919114	1.00176453
H	-2.95214524	-1.41055242	0.74752709
H	-0.35614570	-1.92574457	-1.22863500
H	-0.32432507	2.43463731	-0.77104751
C	0.26670792	0.64053417	0.26514767
C	1.36056098	-0.11849592	0.73509313
C	2.66865697	0.31227582	0.52196247
C	2.91380322	1.50803857	-0.16385797

C	1.82973082	2.26111543	−0.62421171
C	0.51103600	1.84444482	−0.41750371
H	1.17626203	−1.04443942	1.27836242
H	3.49619665	−0.28633499	0.89615679
H	3.93300122	1.84782075	−0.33028027
H	2.00469822	3.19550773	−1.15330382

Formaldehyde–1-phenylselenourea

E: −2973.04
H: −2861.02
G: −2896.77
N_{imag} = 0

N	−0.99956755	0.05284079	0.37954009
N	−3.22071242	−0.46572825	0.40411813
C	−2.27330779	0.49921734	0.16922890
C	−0.63440783	−3.04021609	−0.74398404
Se	−2.83419999	2.17329930	−0.38212261
O	−1.00525354	−2.93394302	0.41146438
H	−0.50798139	−4.03458262	−1.21938668
H	−4.18316252	−0.15368839	0.41064737
H	−0.94224886	−0.91172649	0.71444150
H	−2.99153429	−1.33047337	0.88642694
H	−0.40824423	−2.14996190	−1.36733862
H	−0.31393785	2.40460354	−0.88051153
C	0.27677488	0.63576492	0.19777805
C	1.36415280	−0.09337823	0.72291243
C	2.66888094	0.37386975	0.57288687
C	2.91335921	1.57685118	−0.10051848
C	1.83378673	2.29727804	−0.62012810
C	0.51977043	1.84028439	−0.48142827
H	1.17750398	−1.02248518	1.25911240
H	3.49364706	−0.20008595	0.98937080
H	3.92954391	1.94616912	−0.21482555
H	2.00886522	3.23331105	−1.14626149

Formaldehyde–1-[3-(trifluoromethyl)phenyl]urea

E: −3469.63
H: −3351.95
G: −3390.27
N_{imag} = 0

N	−0.98280576	0.23205759	0.24931064
N	−3.16734823	−0.39925818	0.69137348
C	−2.27820743	0.65981892	0.54267842
C	−0.14688563	−3.43446941	−0.80369116
O	−2.61342545	1.83236312	0.69774919
O	−1.02229814	−2.72485604	−0.34455027
H	−0.35257442	−4.48096718	−1.10964444
H	−4.14289122	−0.12985193	0.70937194
H	−0.85380684	−0.77184080	0.13236095
H	−2.95080804	−1.31464032	0.31246616
H	0.89330988	−3.06975842	−0.93804959
H	1.34954795	−0.76557048	−0.30211745
C	0.16636071	1.00959605	0.05117765
C	0.19433450	2.41602607	0.12766566
C	1.39427610	3.09406690	−0.09869383
C	2.57624379	2.41355612	−0.40075247
C	2.54369738	1.01468444	−0.47411474
C	1.35688219	0.31889942	−0.24660737
H	−0.71850341	2.94945252	0.35890373
H	1.40281335	4.17995904	−0.03978432
H	3.50088452	2.95272662	−0.58015376

C	3.81239372	0.24548160	−0.74206202
F	4.65400734	0.90068494	−1.60014128
F	3.57210564	−0.99545422	−1.29045488
F	4.53210994	0.01887136	0.41110628

Formaldehyde–1-[3-(trifluoromethyl)phenyl]thiourea

E: −3389.73
H: −3273.22
G: −3312.34
N_{imag} = 0

N	−0.94336428	0.22638416	0.23484750
N	−3.09811587	−0.39436637	0.65344835
C	−2.21668862	0.65546620	0.54123713
C	−0.28752804	−3.47191346	−0.92779589
S	−2.73407419	2.23132120	0.80591558
O	−1.07738698	−2.72578157	−0.37846951
H	−0.58702577	−4.48849115	−1.25517034
H	−4.07187733	−0.14945056	0.77051820
H	−0.85218776	−0.78454500	0.12589352
H	−2.86807388	−1.32679091	0.32441057
H	0.76222950	−3.17027151	−1.12547924
H	−0.41871699	2.93930701	0.19117969
C	0.26786976	0.90532526	0.01354409
C	1.38686495	0.08341542	−0.22798123
C	2.63652629	0.64008609	−0.49043141
C	2.80716698	2.03139847	−0.50478477
C	1.69824116	2.84174878	−0.25584171
C	0.43502644	2.30163726	0.00109938
H	1.27283707	−0.99598315	−0.20875709
C	3.82194723	−0.26718859	−0.70301464
H	3.78099234	2.46450161	−0.71023623
F	4.52612096	−0.47909102	0.46097359
F	4.71405243	0.23862043	−1.60920109
F	3.45526839	−1.51528693	−1.15969825
H	1.81284530	3.92308213	−0.26504701

Formaldehyde–1-[3-(trifluoromethyl)phenyl]selenourea

E: −3368.51
H: −3252.95
G: −3291.38
N_{imag} = 0

N	−0.96629331	0.24241420	0.12693599
N	−3.16341246	−0.34225368	0.24253217
C	−2.26258617	0.68181690	0.18568985
C	−0.15937843	−3.56675657	−0.34429664
Se	−2.88606305	2.42358160	0.21443550
O	−1.01944704	−2.76566427	−0.02526532
H	−0.40243813	−4.63517535	−0.51538437
H	−4.14252402	−0.09647515	0.28831598
H	−0.86381839	−0.77437594	0.15223083
H	−2.89154790	−1.31993477	0.21039599
H	0.90032233	−3.26514013	−0.47771729
H	−0.41467618	2.92467315	−0.23338166
C	0.27435575	0.90159405	0.03188616
C	1.40804804	0.07105334	0.12486537
C	2.69062586	0.60311380	0.01038389
C	2.87502988	1.97911237	−0.18377260
C	1.74827075	2.79879198	−0.26742382
C	0.45416624	2.28113793	−0.16556901
H	1.28071344	−0.99447190	0.28935944
C	3.88524402	−0.30549746	0.15462194
H	3.87454643	2.39290679	−0.27291302

F	4.36256693	−0.33136584	1.44561247
F	4.93294767	0.07562765	−0.63886424
F	3.59423511	−1.61321630	−0.17107076
H	1.87387712	3.86781411	−0.42194160

Formaldehyde–1-[3,5-bis(trifluoromethyl)phenyl]urea

E: −3863.05
H: −3741.30
G: −3784.01
N_{imag} = 0

N	−0.97865241	0.12952767	0.03490702
N	−3.23617740	−0.37668520	−0.08534019
C	−2.27220850	0.61413700	−0.19128587
C	−0.27469045	−3.74323726	0.29661636
O	−2.54539534	1.78922533	−0.42219710
O	−1.11242036	−2.86402552	0.21779704
H	−0.55386721	−4.81478764	0.23205209
H	−4.15800324	−0.11343544	−0.40927532
H	−0.89058879	−0.87234729	0.19918796
H	−2.98840495	−1.35873536	−0.12774571
H	0.80289592	−3.51433710	0.43569218
H	−0.59059356	2.79700717	−0.37811427
C	0.22268913	0.84340967	0.02587821
C	1.40389549	0.11180606	0.26058567
C	2.64349387	0.74631178	0.25910305
C	2.74441115	2.12340134	0.03163558
C	1.56896849	2.84406539	−0.19385940
C	0.31338330	2.22912284	−0.20177312
H	1.34627911	−0.95738709	0.44153816
C	3.88813935	−0.05216293	0.56621352
H	3.70888825	2.61722398	0.03122475
C	1.64715401	4.32617816	−0.49543749
F	2.80836181	4.89081471	−0.03827116
F	0.61261403	5.02486289	0.06219575
F	1.60027104	4.56508995	−1.84988841
F	4.98148076	0.40103074	−0.11749566
F	3.74415446	−1.38478206	0.25651459
F	4.21019671	−0.00007429	1.90308728

Formaldehyde–1-[3,5-bis(trifluoromethyl)phenyl]thiourea

E: −3782.83
H: −3662.28
G: −3705.82
N_{imag} = 0

N	−1.03854796	0.20608217	0.05181554
N	−3.21969139	−0.45721752	−0.01712838
C	−2.35287499	0.60238049	−0.11125092
C	−0.14210512	−3.65482475	0.21676287
S	−2.92572581	2.15816220	−0.36574473
O	−1.00525183	−2.79613712	0.23084602
H	−0.40062915	−4.73119383	0.15235242
H	−4.19412875	−0.26409484	−0.20319190
H	−0.91189981	−0.79525342	0.20562784
H	−2.90346389	−1.42132050	0.00199734
H	0.93713311	−3.40014956	0.26684883
H	−0.56568618	2.88413763	−0.40719436
C	0.17426347	0.90899558	0.03088509
C	1.32980055	0.14035207	0.27982794
C	2.59173024	0.72590964	0.26527637
C	2.74253188	2.09464821	0.01265385
C	1.59412697	2.85210543	−0.22505405
C	0.31596965	2.28358961	−0.22217888

H	1.23250234	−0.92112297	0.48487030
C	3.80627176	−0.11369107	0.58331306
H	3.72440213	2.55331628	0.00278803
C	1.72592376	4.32490644	−0.55559173
F	2.89576714	4.86012630	−0.08495506
F	0.70379393	5.06698214	−0.03542143
F	1.71622748	4.53434851	−1.91537134
F	4.90568848	0.26818585	−0.13272379
F	3.60034422	−1.44955874	0.32217295
F	4.15144776	−0.02943669	1.91166064

Formaldehyde–1-[3,5-bis(trifluoromethyl)phenyl]selenourea

E: −3761.56
H: −3641.37
G: −3685.80
N_{imag} = 0

N	−1.04517801	0.18928275	0.09720800
N	−3.23210999	−0.44544651	0.06069290
C	−2.35595956	0.59940690	0.07293091
C	−0.08426563	−3.60350248	−0.08353648
Se	−3.00292862	2.32888063	0.08220039
O	−0.98141561	−2.80110738	0.10359799
H	−0.29558153	−4.68467117	−0.20849460
H	−4.21803332	−0.22464657	0.09062619
H	−0.91405439	−0.82198683	0.17397554
H	−2.93512639	−1.41573541	0.09982132
H	0.97878935	−3.28940222	−0.13736659
H	−0.56546172	2.82672110	−0.52719381
C	0.17091670	0.89124303	0.07156997
C	1.31850012	0.14414752	0.39519326
C	2.58231006	0.73008343	0.35752489
C	2.73423576	2.07682007	0.01371060
C	1.58901544	2.81405665	−0.30146357
C	0.31395788	2.24286431	−0.28407002
H	1.21637633	−0.89786293	0.68347281
C	3.78490975	−0.09252003	0.75476082
H	3.71550553	2.53660970	−0.00942070
C	1.74094076	4.25526704	−0.74328500
F	2.80569117	4.86973434	−0.13676669
F	0.63372991	5.00476730	−0.47618773
F	1.96217678	4.33915447	−2.10005321
F	4.93594229	0.34136089	0.16293476
F	3.63497653	−1.42074160	0.41913662
F	4.00006041	−0.06254946	2.11237398

Formaldehyde–1,3-dicyclohexylurea

E: −5606.83
H: −5353.74
G: −5401.58
N_{imag} = 0

H	−0.05777810	1.09467044	−1.13238953
H	0.05777810	−1.09467044	−1.13238953
N	0.26463644	−1.13583656	−0.13835780
C	−0.00000000	0.00000000	0.61597988
O	−0.00000000	0.00000000	1.85359185
N	−0.26463644	1.13583656	−0.13835780
H	2.17816014	4.80703455	1.40201692
H	0.74561562	4.35425424	2.32397147
H	0.76309224	5.54873471	−0.50629247
H	0.25222783	6.37944570	0.96460919
C	−0.25665674	2.46337665	0.48155254
C	−1.13301874	4.84558631	0.27584117

C	-1.11690114	3.43516896	-0.34590272
C	1.17339261	3.01680103	0.67476917
C	1.15431624	4.42282332	1.30447932
C	0.29235914	5.39617597	0.47753237
H	-0.70958609	2.33719862	1.47217926
H	-1.64241824	4.80069532	1.25026630
H	-1.72011160	5.52610073	-0.35487060
H	-0.70674404	3.49428040	-1.36763972
H	-2.13552232	3.03639839	-0.43198616
H	1.66841728	3.05966900	-0.30855927
H	1.74277541	2.31789502	1.29800555
C	-1.17339261	-3.01680103	0.67476917
C	-1.15431624	-4.42282332	1.30447932
C	0.25665674	-2.46337665	0.48155254
H	-1.74277541	-2.31789502	1.29800555
H	-1.66841728	-3.05966900	-0.30855927
C	-0.29235914	-5.39617597	0.47753237
H	-2.17816014	-4.80703455	1.40201692
H	-0.74561562	-4.35425424	2.32397147
C	1.11690114	-3.43516896	-0.34590272
H	0.70958609	-2.33719862	1.47217926
C	1.13301874	-4.84558631	0.27584117
H	-0.25222783	-6.37944570	0.96460919
H	-0.76309224	-5.54873471	-0.50629247
H	2.13552232	-3.03639839	-0.43198616
H	0.70674404	-3.49428040	-1.36763972
H	1.72011160	-5.52610073	-0.35487060
H	1.64241824	-4.80069532	1.25026630
C	0.00000000	0.00000000	-4.29855089
H	0.08706112	0.94147473	-4.88262234
H	-0.08706112	-0.94147473	-4.88262234
O	0.00000000	0.00000000	-3.08422616

Formaldehyde–1,3-dicyclohexylthiourea

E: −5531.02
H: −5280.22
G: −5324.90
N_{imag} = 0

H	-0.06778414	1.05314379	-1.01341577
H	0.06591666	-1.05336422	-1.01334941
N	0.17041779	-1.13499789	-0.00483617
C	-0.00104085	-0.00006341	0.74210287
S	-0.00116237	-0.00001850	2.43750596
N	-0.17239021	1.13483175	-0.00491800
H	2.15896043	4.92559541	1.37825902
H	0.70842080	4.50097236	2.28540449
H	0.78770776	5.52904054	-0.60915990
H	0.22023066	6.43482594	0.79511822
C	-0.20968316	2.49517064	0.53660689
C	-1.11869875	4.83912817	0.15987156
C	-1.06241366	3.39470273	-0.37586057
C	1.20629534	3.07881850	0.73129450
C	1.14476172	4.51832618	1.27547706
C	0.29128476	5.42565705	0.36844788
H	-0.68635082	2.41921625	1.52103652
H	-1.65568883	4.84235381	1.12009828
H	-1.69803517	5.46976505	-0.52715699
H	-0.62517125	3.40023460	-1.38813718
H	-2.07247717	2.97437849	-0.46298037
H	1.72772790	3.07090198	-0.23890569
H	1.76357402	2.42614060	1.41215680
C	-1.20842796	-3.07892005	0.73124541
C	-1.14701489	-4.51840235	1.27550880

C	0.20759432	−2.49531037	0.53676466
H	−1.76580692	−2.42619786	1.41198338
H	−1.72969995	−3.07104030	−0.23904142
C	−0.29340519	−5.42579488	0.36866651
H	−2.16123922	−4.92564529	1.37814161
H	−0.71084177	−4.50100777	2.28550809
C	1.06046071	−3.39490573	−0.37551343
H	0.68409894	−2.41931249	1.52126971
C	1.11662578	−4.83930616	0.16029970
H	−0.22244498	−6.43494503	0.79539671
H	−0.78966594	−5.52921398	−0.60901987
H	2.07054740	−2.97460592	−0.46248271
H	0.62338973	−3.40047900	−1.38786387
H	1.69606568	−5.46998875	−0.52659956
H	1.65345322	−4.84249536	1.12061755
C	−0.00026440	−0.00015061	−4.14688062
H	0.01992670	0.94521176	−4.72958873
H	−0.02008999	−0.94549871	−4.72962429
O	−0.00065231	−0.00016547	−2.93153970

Formaldehyde–1,3-dicyclohexylselenourea

E: −5511.05
H: −5260.55
G: −5305.91
N_{imag} = 0

H	−0.06871339	1.05614501	−1.01483806
H	0.06783163	−1.05639429	−1.01475180
N	0.14430769	−1.13696759	−0.00258851
C	−0.00124661	−0.00010623	0.73086577
Se	−0.00212658	−0.00009106	2.59901886
N	−0.14610158	1.13673998	−0.00274550
H	2.16159996	4.94364855	1.36689495
H	0.71781840	4.50794741	2.27967258
H	0.77837082	5.53759018	−0.61518379
H	0.20954225	6.43886423	0.79140085
C	−0.19269883	2.49920808	0.53636993
C	−1.12014285	4.83389819	0.16105142
C	−1.05573861	3.38968938	−0.37465012
C	1.22014399	3.09039173	0.72386875
C	1.14995674	4.52928720	1.26811860
C	0.28646213	5.43035176	0.36429132
H	−0.66431734	2.41992138	1.52323191
H	−1.65355282	4.83308634	1.12316681
H	−1.70654167	5.46000084	−0.52403367
H	−0.62287351	3.39764424	−1.38882396
H	−2.06330937	2.96224790	−0.45644070
H	1.73853952	3.08528039	−0.24794070
H	1.78251827	2.44135962	1.40415929
C	−1.22284546	−3.09042883	0.72282141
C	−1.15334350	−4.52932108	1.26716404
C	0.19024504	−2.49943245	0.53659765
H	−1.78575410	−2.44130811	1.40258484
H	−1.74035214	−3.08527067	−0.24946149
C	−0.28915025	−5.43051324	0.36413449
H	−2.16513107	−4.94354601	1.36503196
H	−0.72211863	−4.50801808	2.27910861
C	1.05399776	−3.39004250	−0.37361788
H	0.66097471	−2.42018770	1.52388756
C	1.11771917	−4.83425089	0.16216842
H	−0.21275481	−6.43903184	0.79132394
H	−0.78018243	−5.53769486	−0.61578676
H	2.06170052	−2.96273738	−0.45449556
H	0.62205916	−3.39795891	−1.38818703

H	1.70466104	−5.46044374	−0.52236875
H	1.65024873	−4.83349310	1.12477169
C	0.00106207	0.00007079	−4.12892038
H	0.01802878	0.94550710	−4.71102382
H	−0.01530723	−0.94521687	−4.71128183
O	0.00043713	−0.00008462	−2.91269435

Formaldehyde–1,3-diphenylurea

E: −4587.19
H: −4421.98
G: −4462.30
N_{imag} = 0

N	0.17471484	3.05648197	−0.24606526
N	0.19881751	0.75011517	−0.24606474
C	0.18676841	1.90329876	0.54188283
C	0.21360525	−0.59524717	0.17070599
C	0.22178803	−1.57612818	−0.84293322
C	0.23687596	−2.93217792	−0.52040817
C	0.24404008	−3.33997667	0.81892908
C	0.23587590	−2.36706478	1.82350231
C	0.22073631	−1.00298838	1.51807934
C	0.15992418	4.40184446	0.17070483
C	0.15173956	5.38272495	−0.84293488
C	0.13665113	6.73877486	−0.52041053
C	0.12948292	7.14657426	0.81892650
C	0.13765717	6.17366300	1.82350024
C	0.15279624	4.80958643	1.51807797
H	0.13225072	6.47504649	2.86907306
H	0.21625181	−1.26508438	−1.88698154
O	0.18676854	1.90329900	1.77104729
H	0.17701025	2.92588943	−1.25328404
H	0.21436909	−0.25472174	2.29912835
H	0.19651917	0.88070722	−1.25328355
H	0.24297452	−3.67150334	−1.31862185
H	0.25575652	−4.39701352	1.07292701
H	0.24128030	−2.66844777	2.86907529
H	0.15727364	5.07168062	−1.88698304
H	0.13054910	7.47809983	−1.31862460
H	0.11776120	8.20361119	1.07292387
H	0.15916352	4.06132021	2.29912738
H	0.19505760	0.95761582	−4.91910048
H	0.17848023	2.84899885	−4.91909197
C	0.18676591	1.90330469	−4.33804757
O	0.18676425	1.90329924	−3.12224829

Formaldehyde–1,3-diphenylthiourea

E: −4504.57
H: −4341.86
G: −4379.59
N_{imag} = 0

N	0.14227043	3.03602369	−0.06349009
N	0.23225054	0.77083317	−0.06276762
C	0.18635320	1.90360441	0.73573897
C	0.18692813	−0.60624153	0.24737387
C	0.70448121	−1.48115864	−0.72992234
C	0.66498974	−2.86203967	−0.54246400
C	0.11215257	−3.39828749	0.62648597
C	−0.40641585	−2.53082705	1.59307808
C	−0.37951482	−1.14469714	1.41561533
C	0.18658842	4.41310470	0.24691265
C	−0.33374840	5.28794416	−0.72889427
C	−0.29492150	6.66880764	−0.54092256

C	0.25993534	7.20495967	0.62709668
C	0.78131156	6.33751955	1.59222714
C	0.75517626	4.95147302	1.41413801
H	1.22209644	6.74026321	2.50174522
H	1.14522639	−1.06688752	−1.63577605
S	0.18439365	1.90433157	2.41062578
H	−0.03829490	2.86353674	−1.04997149
H	−0.77927098	−0.48309629	2.17179392
H	0.41369279	0.94313463	−1.04912443
H	1.07368938	−3.51837072	−1.30773950
H	0.08603368	−4.47438719	0.77964223
H	−0.84555288	−2.93358900	2.50338619
H	−0.77619723	4.87367584	−1.63391371
H	−0.70578798	7.32516028	−1.30501680
H	0.28548260	8.28101209	0.78068657
H	1.15722599	4.28988376	2.16912233
H	0.55473489	1.03003938	−4.73463871
H	−0.17624214	2.77465536	−4.73534419
C	0.18897972	1.90247116	−4.15413267
O	0.18842359	1.90273082	−2.93814686

Formaldehyde–1,3-diphenylselenourea

E: −4482.93
H: −4320.06
G: −4360.41
N_{imag} = 0

N	−0.06014730	1.13085606	0.04731188
N	0.06014730	−1.13085606	0.04731188
C	0.00000000	−0.00000000	0.83291408
C	0.00269119	−2.50491123	0.38443809
C	0.71668479	−3.39204335	−0.44167640
C	0.66856104	−4.76801340	−0.21178301
C	−0.08819818	−5.27680461	0.84946115
C	−0.80441031	−4.39320103	1.66475071
C	−0.77131940	−3.01508027	1.43880763
C	−0.00269119	2.50491123	0.38443809
C	−0.71668479	3.39204335	−0.44167640
C	−0.66856104	4.76801340	−0.21178301
C	0.08819818	5.27680461	0.84946115
C	0.80441031	4.39320103	1.66475071
C	0.77131940	3.01508027	1.43880763
H	1.40434869	4.77790751	2.48667062
H	1.31935120	−2.99427400	−1.25681160
Se	0.00000000	−0.00000000	2.67688616
H	−0.27237192	0.96938591	−0.93580373
H	−1.32726699	−2.33689784	2.07298175
H	0.27237192	−0.96938591	−0.93580373
H	1.23123964	−5.43966249	−0.85639729
H	−0.12052806	−6.34734334	1.03715830
H	−1.40434869	−4.77790751	2.48667062
H	−1.31935120	2.99427400	−1.25681160
H	−1.23123964	5.43966249	−0.85639729
H	0.12052806	6.34734334	1.03715830
H	1.32726699	2.33689784	2.07298175
H	0.42550597	−0.84466544	−4.61145154
H	−0.42550597	0.84466544	−4.61145154
C	0.00000000	−0.00000000	−4.03077073
O	0.00000000	−0.00000000	−2.81434258

Formaldehyde–1,3-bis[3-(trifluoromethyl)phenyl]urea

E: −5378.32
H: −5204.75
G: −5252.56

N_{imag} = 0

N	0.00897777	1.15194557	0.23712105
N	−0.00897777	−1.15194557	0.23712105
C	0.00000000	−0.00000000	1.02685307
C	−0.02229998	−2.49333209	0.65611415
C	−0.02502089	−3.47025066	−0.35798771
C	−0.04493502	−4.82587772	−0.03244235
C	−0.05736069	−5.23903916	1.30657467
C	−0.05353113	−4.26557998	2.30731736
C	−0.03586364	−2.90219407	2.00319160
C	0.02229998	2.49333209	0.65611415
C	0.02502089	3.47025066	−0.35798771
C	0.04493502	4.82587772	−0.03244235
C	0.05736069	5.23903916	1.30657467
C	0.05353113	4.26557998	2.30731736
C	0.03586364	2.90219407	2.00319160
F	0.47496786	5.37870915	−2.32830831
H	−0.01822349	−3.16501933	−1.40094739
O	0.00000000	−0.00000000	2.25514199
H	0.00482112	1.02017145	−0.77084542
H	−0.03464821	−2.15467091	2.78499001
H	−0.00482112	−1.02017145	−0.77084542
C	0.00710607	−5.85628083	−1.13340533
H	−0.07544453	−6.29522280	1.55569429
F	0.71549839	6.98013780	−0.83286883
H	0.01822349	3.16501933	−1.40094739
C	−0.00710607	5.85628083	−1.13340533
H	0.07544453	6.29522280	1.55569429
H	0.03464821	2.15467091	2.78499001
F	1.29632919	−6.27756200	−1.37947160
F	−0.47496786	−5.37870915	−2.32830831
F	−0.71549839	−6.98013780	−0.83286883
F	−1.29632919	6.27756200	−1.37947160
H	0.06663936	4.57044849	3.35105584
H	−0.06663936	−4.57044849	3.35105584
H	−0.00609724	−0.94597226	−4.39717925
H	0.00609724	0.94597226	−4.39717925
C	−0.00000000	−0.00000000	−3.81822171
O	−0.00000000	−0.00000000	−2.60097896

Formaldehyde–1,3-bis[3-(trifluoromethyl)phenyl]thiourea

E: −5295.39
H: −5123.61
G: −5170.58
N_{imag} = 0

N	−0.94076821	0.61734903	0.55031729
N	1.22600294	0.03615879	0.25421073
C	0.35579668	0.81517389	1.00355377
C	2.61705638	−0.17836268	0.30117604
C	3.13073630	−0.99011925	−0.73201836
C	4.48840786	−1.29416973	−0.78817418
C	5.37224561	−0.79252880	0.17797451
C	4.86013049	0.00909077	1.19800836
C	3.49977127	0.32192010	1.27490611
C	−2.18651602	1.16478353	0.91319843
C	−3.30003902	0.60222592	0.25448612
C	−4.58693761	1.06842596	0.51026291
C	−4.80083031	2.10171143	1.43374930
C	−3.69714822	2.65544448	2.08227874
C	−2.39687130	2.20495922	1.83591392
F	−5.41816685	−0.22218935	−1.33555214
H	2.45843694	−1.38667790	−1.48803926

S	0.79998190	1.83285680	2.25641470
H	−1.03779282	−0.07848470	−0.18665195
H	3.11776807	0.94486381	2.07143909
H	0.79223324	−0.48412247	−0.50596904
C	5.02172399	−2.12368163	−1.93015744
H	6.43011935	−1.03138878	0.13260995
F	−6.74685631	1.31936721	−0.48827194
H	−3.15055584	−0.19905152	−0.46413900
C	−5.76626269	0.41785202	−0.16987515
H	−5.80482673	2.46401097	1.63107927
H	−1.55137766	2.64392904	2.34669382
F	5.54116963	−1.33795171	−2.93521384
F	4.05325114	−2.90500020	−2.51430481
F	6.02862173	−2.96493084	−1.53756771
F	−6.35824580	−0.53407016	0.63058334
H	−3.84423212	3.46154157	2.79709358
H	5.53040996	0.40169897	1.95890960
H	−0.17954655	−2.67711109	−3.25910577
H	−2.00359903	−2.32474359	−2.89863577
C	−0.93818476	−2.14949652	−2.64622722
O	−0.61510564	−1.40928258	−1.73495602

Formaldehyde–1,3-bis[3-(trifluoromethyl)phenyl]selenourea

E: −5273.48
H: −5102.81
G: −5148.41
N_{imag} = 0

N	0.05068670	1.12932955	0.36387432
N	−0.05068670	−1.12932955	0.36387432
C	−0.00000000	−0.00000000	1.15468111
C	−0.21294794	−2.49562229	0.68036322
C	0.31566766	−3.41173652	−0.24545370
C	0.15539263	−4.78454210	−0.05052489
C	−0.51989320	−5.26777972	1.07748801
C	−1.04862306	−4.35187186	1.98963514
C	−0.91079299	−2.97496174	1.80158445
C	0.21294794	2.49562229	0.68036322
C	−0.31566766	3.41173652	−0.24545370
C	−0.15539263	4.78454210	−0.05052489
C	0.51989320	5.26777972	1.07748801
C	1.04862306	4.35187186	1.98963514
C	0.91079299	2.97496174	1.80158445
F	−0.84694408	5.22572983	−2.30333253
H	0.85219590	−3.04741731	−1.11757252
Se	0.00000000	0.00000000	2.99647198
H	−0.14501675	0.97710345	−0.62490900
H	−1.33025293	−2.27507505	2.51169097
H	0.14501675	−0.97710345	−0.62490900
C	0.77347904	−5.74802677	−1.03328423
H	−0.63845700	−6.33583388	1.22972142
F	−0.07529258	6.92210222	−1.12525262
H	−0.85219590	3.04741731	−1.11757252
C	−0.77347904	5.74802677	−1.03328423
H	0.63845700	6.33583388	1.22972142
H	1.33025293	2.27507505	2.51169097
F	2.06130900	−6.08605015	−0.68158013
F	0.84694408	−5.22572983	−2.30333253
F	0.07529258	−6.92210222	−1.12525262
F	−2.06130900	6.08605015	−0.68158013
H	1.58962478	4.71326929	2.86072641
H	−1.58962478	−4.71326929	2.86072641
H	0.26696135	−0.90774590	−4.26547080
H	−0.26696135	0.90774590	−4.26547080

C	0.00000000	-0.00000000	-3.68707115
O	0.00000000	-0.00000000	-2.46948305

Formaldehyde–1,3-bis[3,5-bis(trifluoromethyl)phenyl]urea

E: -6164.47
H: -5982.69
G: -6038.97
N_{imag} = 0

H	-2.84625191	-0.05379943	3.97625614
H	-4.89489854	-0.05499483	3.97625304
N	-5.02343068	-0.06273296	2.96742562
C	-3.87057256	-0.05442479	2.17785454
H	-7.05267928	-0.04798157	4.59002750
N	-2.71771728	-0.04607840	2.96742894
H	-1.73995370	0.00573026	0.41425462
O	-3.87056930	-0.05443441	0.95127873
C	-1.38150003	-0.03437292	2.53976736
C	-0.39397619	-0.04501952	3.54435410
C	0.95625680	-0.02536141	3.20602744
C	1.36018423	0.00015245	1.86574433
C	0.37536466	0.01152327	0.87700857
C	-0.98777107	-0.00692195	1.19131152
H	-0.68846437	-0.06083123	4.59003094
C	2.00164408	-0.08775287	4.29617793
H	2.41128378	0.02073431	1.60348295
C	0.76928335	0.00599216	-0.58605054
C	-6.35964774	-0.07443681	2.53976242
C	-6.75338011	-0.10190237	1.19130841
C	-8.11651799	-0.12031892	0.87700832
C	-9.10133444	-0.10893949	1.86574543
C	-8.69740310	-0.08346515	3.20602888
C	-7.34717021	-0.06377906	3.54435130
H	-6.00119968	-0.11452065	0.41424850
C	-8.51042896	-0.11479193	-0.58605288
H	-10.15243438	-0.12952223	1.60348662
C	-9.74279029	-0.02113231	4.29618262
F	-8.32282152	1.12456933	-1.14965129
F	-9.82535447	-0.44091350	-0.77493812
F	-7.76566750	-0.99720859	-1.32120397
F	2.08414742	0.33236140	-0.77494414
F	0.58192908	-1.23344212	-1.14957589
F	0.02435351	0.88821441	-1.32125995
F	2.42588820	-1.37778117	4.51596089
F	1.53076333	0.37672316	5.49912180
F	3.11469952	0.64432188	3.98992935
F	-10.16708208	1.26887694	4.51598153
F	-9.27189124	-0.48560393	5.49912099
F	-10.85582058	-0.75324299	3.98992614
C	-3.87057802	-0.05438139	7.00502259
O	-3.87057756	-0.05438784	5.78709157
H	-4.81640363	-0.05266208	7.58344124
H	-2.92475280	-0.05609983	7.58344187

C_{2v} symmetry enforced
E: -6164.31
H: -5983.15
G: -6037.12
N_{imag} = 4 (-5.7; -6.0; -6.2; -7.3 cm^{-1})

H	1.02250541	0.00000000	1.38235625
H	-1.02250541	0.00000000	1.38235625
N	-1.15253537	0.00000000	0.37363306
C	-0.00000000	0.00000000	-0.41666650

H	-3.18211674	0.00000000	1.99725311
N	1.15253537	0.00000000	0.37363306
H	2.13140298	0.00000000	-2.17968708
O	-0.00000000	0.00000000	-1.64328007
C	2.48998555	0.00000000	-0.05042175
C	3.47551039	0.00000000	0.95014550
C	4.83008190	0.00000000	0.61014191
C	5.23131753	0.00000000	-0.72532074
C	4.24212985	0.00000000	-1.71742078
C	2.88377876	0.00000000	-1.40405837
H	3.18211674	0.00000000	1.99725311
C	5.84560695	0.00000000	1.72960261
H	6.28284966	0.00000000	-0.98830178
C	4.68906191	0.00000000	-3.16473588
C	-2.48998555	0.00000000	-0.05042175
C	-2.88377876	0.00000000	-1.40405837
C	-4.24212985	0.00000000	-1.71742078
C	-5.23131753	0.00000000	-0.72532074
C	-4.83008190	0.00000000	0.61014191
C	-3.47551039	0.00000000	0.95014550
H	-2.13140298	0.00000000	-2.17968708
C	-4.68906191	0.00000000	-3.16473588
H	-6.28284966	0.00000000	-0.98830178
C	-5.84560695	0.00000000	1.72960261
F	-5.46176589	1.10034866	-3.45023031
F	-5.46176589	-1.10034866	-3.45023031
F	-3.64910062	0.00000000	-4.04483698
F	5.46176589	1.10034866	-3.45023031
F	5.46176589	-1.10034866	-3.45023031
F	3.64910062	0.00000000	-4.04483698
F	5.70135442	-1.09979642	2.54467826
F	5.70135442	1.09979642	2.54467826
F	7.13203689	0.00000000	1.28058648
F	-5.70135442	1.09979642	2.54467826
F	-5.70135442	-1.09979642	2.54467826
F	-7.13203689	0.00000000	1.28058648
C	-0.00000000	0.00000000	4.41131358
O	-0.00000000	0.00000000	3.19337973
H	-0.94577903	0.00000000	4.98979515
H	0.94577903	0.00000000	4.98979515

Formaldehyde–1,3-bis[3,5-bis(trifluoromethyl)phenyl]thiourea

E: −6081.27
H: −5901.44
G: −5956.41
N_{imag} = 0

H	-0.16306477	-0.96533739	1.12358460
H	0.16306477	0.96533739	1.12358460
N	0.00299116	1.13279501	0.13155202
C	0.00000000	-0.00000000	-0.67231653
H	0.77834192	2.99363012	1.74610743
N	-0.00299116	-1.13279501	0.13155202
H	0.93947786	-2.35590241	-2.15113500
S	0.00000000	-0.00000000	-2.34127943
C	0.06257729	-2.50157033	-0.18418685
C	-0.38132521	-3.38800348	0.81455216
C	-0.31105357	-4.76633892	0.62065997
C	0.18817512	-5.29506591	-0.57363964
C	0.63461730	-4.40899815	-1.55749232
C	0.58290351	-3.02374531	-1.38012351
H	-0.77834192	-2.99363012	1.74610743
C	-0.83782155	-5.69130886	1.69404052
H	0.24215842	-6.36681571	-0.72554021

C	1.15207985	−4.95856422	−2.87110835
C	−0.06257729	2.50157033	−0.18418685
C	−0.58290351	3.02374531	−1.38012351
C	−0.63461730	4.40899815	−1.55749232
C	−0.18817512	5.29506591	−0.57363964
C	0.31105357	4.76633892	0.62065997
C	0.38132521	3.38800348	0.81455216
H	−0.93947786	2.35590241	−2.15113500
C	−1.15207985	4.95856422	−2.87110835
H	−0.24215842	6.36681571	−0.72554021
C	0.83782155	5.69130886	1.69404052
F	−0.13989444	5.08857489	−3.79136343
F	−1.71280487	6.19976393	−2.72532583
F	−2.09910940	4.15082991	−3.43590863
F	1.71280487	−6.19976393	−2.72532583
F	0.13989444	−5.08857489	−3.79136343
F	2.09910940	−4.15082991	−3.43590863
F	−2.18298622	−5.93091442	1.54142218
F	−0.67843444	−5.16515689	2.95367203
F	−0.21653902	−6.90762137	1.68465936
F	2.18298622	5.93091442	1.54142218
F	0.67843444	5.16515689	2.95367203
F	0.21653902	6.90762137	1.68465936
C	−0.00000000	−0.00000000	4.17466944
O	−0.00000000	−0.00000000	2.95655845
H	0.26148519	0.90916412	4.75262215
H	−0.26148519	−0.90916412	4.75262215

C_{2v} symmetry enforced
E: −6081.00
H: −5901.09
G: −5956.26
N_{imag} = 6 (−8.8; −9.9; −10.9 −13.4; −13.5; −14.1 cm^{-1})

H	0.94885540	0.00000000	1.03489333
H	−0.94885540	−0.00000000	1.03489333
N	−1.13152530	0.00000000	0.03289127
C	0.00000000	−0.00000000	−0.77388354
H	−2.91402575	−0.00000000	1.88154573
N	1.13152530	−0.00000000	0.03289127
H	2.45970883	−0.00000000	−2.40053103
S	0.00000000	0.00000000	−2.44417857
C	2.50974212	−0.00000000	−0.24251165
C	3.35077864	−0.00000000	0.88565130
C	4.73814415	−0.00000000	0.74120099
C	5.32332347	−0.00000000	−0.52546146
C	4.48181753	−0.00000000	−1.64404044
C	3.09210197	−0.00000000	−1.52475634
H	2.91402575	0.00000000	1.88154573
C	5.58453298	0.00000000	1.99358750
H	6.40139658	−0.00000000	−0.63873523
C	5.12358586	0.00000000	−3.01674841
C	−2.50974212	0.00000000	−0.24251165
C	−3.09210197	0.00000000	−1.52475634
C	−4.48181753	0.00000000	−1.64404044
C	−5.32332347	0.00000000	−0.52546146
C	−4.73814415	0.00000000	0.74120099
C	−3.35077864	0.00000000	0.88565130
H	−2.45970883	0.00000000	−2.40053103
C	−5.12358586	−0.00000000	−3.01674841
H	−6.40139658	0.00000000	−0.63873523
C	−5.58453298	−0.00000000	1.99358750
F	−5.92789139	1.10034949	−3.19294218
F	−5.92789139	−1.10034949	−3.19294218
F	−4.21392470	−0.00000000	−4.03060124

F	5.92789139	1.10034949	−3.19294218
F	5.92789139	−1.10034949	−3.19294218
F	4.21392470	0.00000000	−4.03060124
F	5.32551027	−1.09969657	2.78019390
F	5.32551027	1.09969657	2.78019390
F	6.92112199	0.00000000	1.73156037
F	−5.32551027	1.09969657	2.78019390
F	−5.32551027	−1.09969657	2.78019390
F	−6.92112199	−0.00000000	1.73156037
C	0.00000000	0.00000000	4.09391448
O	0.00000000	−0.00000000	2.87558889
H	−0.94586984	0.00000000	4.67183125
H	0.94586984	−0.00000000	4.67183125

Formaldehyde–1,3-bis[3,5-bis(trifluoromethyl)phenyl]selenourea

E: −6059.59
H: −5880.29
G: −5935.88
N_{imag} = 0

H	−0.21862778	−0.99217372	1.27411188
H	0.21862778	0.99217372	1.27411188
N	0.00946360	1.13398771	0.28611229
C	0.00000000	−0.00000000	−0.49979239
H	1.29862218	3.06655476	1.47417451
N	−0.00946360	−1.13398771	0.28611229
H	1.47432550	−2.24039428	−1.71292951
Se	0.00000000	−0.00000000	−2.33772972
C	0.07042182	−2.49139515	−0.09203797
C	−0.66058200	−3.41527739	0.66655583
C	−0.58818288	−4.78035451	0.37254014
C	0.20264502	−5.24004238	−0.68161901
C	0.93912466	−4.31000852	−1.42498023
C	0.88791965	−2.94481049	−1.13970038
H	−1.29862218	−3.06655476	1.47417451
C	−1.34485151	−5.75597942	1.24356920
H	0.25206138	−6.29745761	−0.91465616
C	1.78059849	−4.79495867	−2.58626176
C	−0.07042182	2.49139515	−0.09203797
C	−0.88791965	2.94481049	−1.13970038
C	−0.93912466	4.31000852	−1.42498023
C	−0.20264502	5.24004238	−0.68161901
C	0.58818288	4.78035451	0.37254014
C	0.66058200	3.41527739	0.66655583
H	−1.47432550	2.24039428	−1.71292951
C	−1.78059849	4.79495867	−2.58626176
H	−0.25206138	6.29745761	−0.91465616
C	1.34485151	5.75597942	1.24356920
F	−1.03480511	4.90609725	−3.73433441
F	−2.31918842	6.03279844	−2.34738984
F	−2.81792915	3.95023759	−2.86665148
F	2.31918842	−6.03279844	−2.34738984
F	1.03480511	−4.90609725	−3.73433441
F	2.81792915	−3.95023759	−2.86665148
F	−2.57498876	−5.27189602	1.61173398
F	−0.66569375	−6.01364621	2.41544864
F	−1.54830407	−6.95884817	0.63303229
F	2.57498876	5.27189602	1.61173398
F	0.66569375	6.01364621	2.41544864
F	1.54830407	6.95884817	0.63303229
C	0.00000000	−0.00000000	4.31681001
O	−0.00000000	−0.00000000	3.09873131
H	0.33417087	0.88491974	4.89513701
H	−0.33417087	−0.88491974	4.89513701

***C_{2v}* symmetry enforced**
E: −6058.87
H: −5879.91
G: −5933.79
N_{imag} = 6 (−8.1; −9.3; −10.0; −11.9; −14.9; −15.1 cm^{-1})

H	0.94017144	0.00000000	0.98536820
H	−0.94017144	−0.00000000	0.98536820
N	−1.12800126	−0.00000000	−0.01663463
C	0.00000000	0.00000000	−0.81756555
H	−2.87211020	−0.00000000	1.86314494
N	1.12800126	0.00000000	−0.01663463
H	2.50431446	−0.00000000	−2.42670295
Se	0.00000000	0.00000000	−2.65652610
C	2.51204769	−0.00000000	−0.26896379
C	3.32939730	0.00000000	0.87648209
C	4.71933356	0.00000000	0.76093451
C	5.33038637	−0.00000000	−0.49366808
C	4.51206833	−0.00000000	−1.62913286
C	3.12022807	−0.00000000	−1.53821977
H	2.87211020	0.00000000	1.86314494
C	5.53937670	0.00000000	2.03064432
H	6.41061704	−0.00000000	−0.58469889
C	5.18073150	−0.00000000	−2.98902736
C	−2.51204769	0.00000000	−0.26896379
C	−3.12022807	0.00000000	−1.53821977
C	−4.51206833	0.00000000	−1.62913286
C	−5.33038637	0.00000000	−0.49366808
C	−4.71933356	−0.00000000	0.76093451
C	−3.32939730	−0.00000000	0.87648209
H	−2.50431446	0.00000000	−2.42670295
C	−5.18073150	0.00000000	−2.98902736
H	−6.41061704	0.00000000	−0.58469889
C	−5.53937670	−0.00000000	2.03064432
F	−5.98839991	1.10035337	−3.14918275
F	−5.98839991	−1.10035337	−3.14918275
F	−4.29095385	0.00000000	−4.02002597
F	5.98839991	1.10035337	−3.14918275
F	5.98839991	−1.10035337	−3.14918275
F	4.29095385	−0.00000000	−4.02002597
F	5.26382827	−1.09967487	2.81176008
F	5.26382827	1.09967487	2.81176008
F	6.88101296	0.00000000	1.79658223
F	−5.26382827	1.09967487	2.81176008
F	−5.26382827	−1.09967487	2.81176008
F	−6.88101296	−0.00000000	1.79658223
C	0.00000000	0.00000000	4.04865235
O	0.00000000	0.00000000	2.83014374
H	−0.94587956	0.00000000	4.62641112
H	0.94587956	−0.00000000	4.62641112

Formaldehyde–1-phenyl-3-[3,5-bis(trifluoromethyl)phenyl]urea

E: −5376.46
H: −5202.98
G: −5251.39
N_{imag} = 0

N	−0.89608056	−1.06096431	−0.07995655
N	−0.82598198	1.24078745	−0.00072550
C	−0.09439564	0.06876543	0.15004694
C	−0.38579265	2.57548452	0.14395318
C	−1.34395274	3.58589320	−0.07092339
C	−0.99467677	4.92992563	0.05121398

C	0.31487625	5.29089737	0.38947577
C	1.26389697	4.28641303	0.60216558
C	0.93101177	2.93364086	0.48401443
C	−0.52375364	−2.40670750	−0.02020827
C	−1.52461546	−3.35801580	−0.30601084
C	−1.24234903	−4.72013574	−0.25917848
C	0.04062196	−5.17707877	0.06573233
C	1.02720469	−4.22998666	0.34726731
C	0.76813294	−2.85612516	0.30855731
C	2.43721442	−4.68117704	0.66716217
H	−2.36433475	3.31026394	−0.33479799
O	1.09629656	0.01419509	0.44460257
H	−1.87258581	−0.89363702	−0.31593755
H	1.66933062	2.16071803	0.64951095
H	−1.80637828	1.14416078	−0.24612587
H	−1.74834887	5.69564132	−0.11871722
H	0.58926637	6.33845119	0.48507251
H	2.28537047	4.55253646	0.86549409
H	−2.52676769	−3.02381998	−0.55831124
C	−2.31801051	−5.71844977	−0.61721846
H	0.25882051	−6.23752418	0.10710091
H	1.54094990	−2.13329865	0.53290258
H	−5.65712158	0.13424392	−1.03173214
H	−4.95559023	−1.61677169	−0.89955645
C	−4.77694011	−0.52166949	−0.87433897
O	−3.66779126	−0.05838052	−0.68275845
F	3.27613645	−4.50547009	−0.40820371
F	2.49973431	−6.00671044	1.00299132
F	2.98102450	−3.97639908	1.70710787
F	−2.23643812	−6.10277706	−1.93597416
F	−3.58366783	−5.21121342	−0.44063398
F	−2.23738788	−6.86131905	0.12873879

Formaldehyde–1-phenyl-3-[3,5-bis(trifluoromethyl)phenyl]thiourea

E: −5293.77
H: −5121.60
G: −5170.72
N_{imag} = 0

N	−0.67144979	−0.99490727	−0.00473801
N	−0.54099844	1.26529960	−0.00882838
C	0.17560588	0.09945260	−0.18328667
C	−0.22228520	2.63475286	−0.16845300
C	−1.31217396	3.52559061	−0.06969786
C	−1.12048928	4.89941586	−0.19308455
C	0.16213566	5.41380385	−0.41977010
C	1.24099476	4.53112946	−0.51540855
C	1.06616261	3.14915690	−0.39030487
C	−0.39744977	−2.36879030	0.02901370
C	−1.45489180	−3.22941793	−0.31198844
C	−1.30323885	−4.61429075	−0.22474111
C	−0.08868666	−5.17442863	0.17550123
C	0.96303908	−4.31234070	0.50958415
C	0.82580235	−2.92482899	0.44792565
C	2.29490251	−4.90671745	0.91654853
H	−2.31307849	3.13039116	0.10105004
S	1.79403818	−0.02493122	−0.58456388
H	−1.67124462	−0.78637540	0.00614862
H	1.90759749	2.47452638	−0.46428472
H	−1.51939541	1.13251251	0.23648211
H	−1.97485449	5.56783300	−0.11479865
H	0.31516631	6.48553835	−0.51886253
H	2.24357036	4.91629565	−0.68813415
H	−2.39721429	−2.80977496	−0.65294184

C	-2.46633984	-5.49318833	-0.61792020
H	0.03248537	-6.24934498	0.23995459
H	1.64592567	-2.27698072	0.72303778
H	-5.42372145	-0.44753601	1.01801323
H	-4.39013616	-1.98472216	0.63657990
C	-4.45737773	-0.87819307	0.68618182
O	-3.51560712	-0.16402800	0.39132770
F	3.06765055	-5.20365937	-0.18081394
F	2.14266490	-6.07742058	1.61495730
F	3.02882518	-4.06248915	1.70100586
F	-2.61233573	-5.57213100	-1.98230028
F	-3.66241981	-5.00129600	-0.13462575
F	-2.34537460	-6.77037789	-0.15589374

Formaldehyde–1-phenyl-3-[3,5-bis(trifluoromethyl)phenyl]selenourea

E: −5272.06
H: −5100.99
G: −5148.90
N_{imag} = 0

N	-0.76886128	-0.94643751	-0.14061027
N	-0.69168936	1.30991148	0.00176507
C	0.05626828	0.15695166	-0.01927190
C	-0.29761843	2.67429841	0.00095473
C	-1.11796913	3.57513038	0.70123448
C	-0.82378348	4.93959989	0.70420494
C	0.29483578	5.41781056	0.01329610
C	1.10359725	4.51890224	-0.69061457
C	0.81386905	3.15250659	-0.70872878
C	-0.48080889	-2.31880542	-0.06011086
C	-1.29944069	-3.18189967	-0.80287455
C	-1.13455977	-4.56738224	-0.71558315
C	-0.13479859	-5.11511619	0.08829123
C	0.68080294	-4.24632138	0.82596404
C	0.51770275	-2.86192132	0.76893528
C	1.78137627	-4.83350025	1.68344453
H	-1.98038042	3.20050819	1.25058684
Se	1.89156923	0.07534210	0.08234873
H	-1.73591464	-0.75587452	-0.41526270
H	1.44135643	2.46217011	-1.25732615
H	-1.69042228	1.18159577	0.15197212
H	-1.46459445	5.62516925	1.25371771
H	0.53116271	6.47901780	0.02071350
H	1.96908553	4.88177911	-1.24034045
H	-2.05883565	-2.76616514	-1.45934025
C	-2.04597622	-5.44808230	-1.53502324
H	-0.00172857	-6.18874042	0.15281683
H	1.14385112	-2.20653056	1.35808872
H	-5.61598907	-0.95447901	-0.24557401
H	-4.34763824	-2.31422071	-0.59065323
C	-4.55818121	-1.23818019	-0.42042939
O	-3.67265150	-0.40147323	-0.42311922
F	2.18706706	-3.98421306	2.67282939
F	-1.78532540	-5.34907640	-2.88028329
F	-3.37131089	-5.08698030	-1.37786768
F	-1.95189239	-6.76664916	-1.20448603
F	2.89354365	-5.13590283	0.93476646
F	1.38999200	-6.00191421	2.28830249

Formaldehyde–1-cyclohexyl-3-[3,5-bis(trifluoromethyl)phenyl]urea

E: −5887.63
H: −5671.38
G: −5719.66
N_{imag} = 0

H	2.92222317	3.83977740	3.42019201
H	2.55835802	4.43777676	1.80291041
H	4.68840259	2.34001763	2.51196857
H	4.99945632	3.99753681	1.99342275
N	0.92408765	0.83850798	0.46190374
N	−1.16655339	−0.13157741	0.25292494
C	−0.42365677	1.06187699	0.27866575
C	−2.53683564	−0.28972768	0.04686322
C	−3.04298149	−1.60549290	0.07222635
C	−4.39788373	−1.85056797	−0.13615533
C	−5.29101765	−0.79844890	−0.36738419
C	−4.78520311	0.50426262	−0.38396781
C	−3.42884497	0.77561010	−0.18357420
C	−5.71403237	1.66165997	−0.68443296
F	−3.95483988	−4.19567953	−0.37560837
F	−5.65223822	2.02411934	−2.01094781
F	−5.40494136	2.77890355	0.04133737
F	−7.02479221	1.36103507	−0.42234201
F	−5.98099700	−3.48619595	−0.87731288
F	−5.33595247	−3.57938686	1.22369838
H	−2.36787943	−2.43608432	0.25499142
O	−0.91667524	2.18110300	0.13266430
H	1.24084568	−0.09519432	0.70335604
H	−3.04826669	1.78825332	−0.20023785
H	−0.64170995	−0.99617784	0.37766491
C	−4.91609153	−3.26611125	−0.04942978
H	−6.34528075	−0.98910248	−0.52862055
C	1.86180903	1.94738569	0.67698063
C	4.28058430	2.68644275	0.41062894
C	3.26630516	1.54614407	0.19308344
C	1.88811924	2.40730681	2.15112383
C	2.89729032	3.55236574	2.36139020
C	4.30539941	3.15501134	1.87854864
H	1.49283216	2.77799057	0.06380840
H	4.00833565	3.53497012	−0.23469907
H	5.27987237	2.35968203	0.09576479
H	3.59648433	0.65563604	0.75255543
H	3.22176960	1.26347416	−0.86619573
H	2.16696392	1.54838105	2.78135404
H	0.87933376	2.71735113	2.44667477
C	0.52140284	−3.67174702	0.42798003
H	1.35024815	−4.39851516	0.55240838
H	−0.46070657	−4.08282607	0.11208256
O	0.68550212	−2.48286740	0.62876908

Formaldehyde–1-cyclohexyl-3-[3,5-bis(trifluoromethyl)phenyl]thiourea

E: −5808.04
H: −5592.41
G: −5642.94
N_{imag} = 0

H	3.24190478	3.46185494	3.66895377
H	2.77147822	4.31472049	2.20015066
H	4.83703712	2.04702330	2.38615602
H	5.19302706	3.74980564	2.09150451
N	0.83395765	1.04281387	0.50227022
N	−1.21774115	0.08110704	0.27530329
C	−0.51108425	1.26996737	0.42515393
C	−2.57941689	−0.16941682	0.06833265
C	−3.03374067	−1.46173131	0.38877651
C	−4.35695934	−1.83291025	0.14959148
C	−5.26817022	−0.92106117	−0.38856972
C	−4.81256610	0.36511657	−0.69994759

C	−3.48797512	0.75173436	−0.48625911
C	−5.79408961	1.37764742	−1.25136895
F	−3.83788144	−4.16864265	0.21788938
F	−5.19012561	2.32440814	−2.02953014
F	−6.44369119	2.04807562	−0.24167960
F	−6.76792244	0.78741973	−2.01652017
F	−5.94819009	−3.60756902	−0.09205395
F	−5.01580375	−3.34358986	1.88355129
H	−2.34549940	−2.17459574	0.83375182
S	−1.19542668	2.80202904	0.52545892
H	1.16194314	0.08046702	0.48037606
H	−3.15120297	1.74736689	−0.74046858
H	−0.66678826	−0.77047435	0.39581968
C	−4.79660175	−3.22565252	0.53125790
H	−6.29698485	−1.20667144	−0.57301998
C	1.84702398	2.06564228	0.78824426
C	4.26863192	2.72681050	0.40704923
C	3.17684037	1.67721566	0.11855290
C	2.02389189	2.28533196	2.30563839
C	3.10932198	3.34152941	2.58635955
C	4.44603128	2.96428614	1.91942173
H	1.47364177	2.99634544	0.34547255
H	3.98967412	3.67422617	−0.07741041
H	5.21665266	2.40887611	−0.04511034
H	3.50374462	0.69807266	0.50525576
H	3.02492955	1.56491070	−0.96250021
H	2.30820444	1.32824397	2.76949245
H	1.06269123	2.58857753	2.73464197
C	0.28958456	−3.45575235	−0.01543448
H	1.06899208	−4.22923133	−0.17190555
H	−0.76353499	−3.76260124	−0.18450259
O	0.58208801	−2.32386914	0.32585928

Formaldehyde–1-cyclohexyl-3-[3,5-bis(trifluoromethyl)phenyl]selenourea

E: −5787.11
H: −5571.85
G: −5623.11
N_{imag} = 0

H	3.29175741	3.29963813	3.75069754
H	2.75639569	4.24638345	2.36377331
H	4.87897877	2.02415875	2.32035983
H	5.18618253	3.75242347	2.14289403
N	0.84286432	1.06169594	0.48754251
N	−1.18830636	0.06657024	0.25208799
C	−0.50158619	1.25132124	0.44033224
C	−2.55484894	−0.18671871	0.05852298
C	−3.03039805	−1.44059784	0.47227154
C	−4.36073461	−1.80604920	0.24932476
C	−5.24876680	−0.92117861	−0.36313498
C	−4.76782629	0.32977406	−0.77178148
C	−3.43781357	0.70505342	−0.57824837
C	−5.73002318	1.30545379	−1.41451686
F	−3.94806138	−4.16028322	0.29609671
F	−5.09721164	2.23921226	−2.18417040
F	−6.45549835	1.99171990	−0.46966606
F	−6.64314442	0.66983152	−2.21907756
F	−6.05336043	−3.50333227	0.25712972
F	−4.85351521	−3.25700095	2.08629628
H	−2.35861511	−2.12601334	0.98165117
Se	−1.29085902	2.91213143	0.65445529
H	1.19412224	0.10963012	0.40449957
H	−3.07837967	1.66887897	−0.91205388
H	−0.63251162	−0.78619883	0.35377005

C	-4.81444083	-3.16769960	0.71509522
H	-6.28205177	-1.20061250	-0.53209293
C	1.84330603	2.08600432	0.81626987
C	4.23430356	2.83332035	0.41458349
C	3.15901924	1.78001730	0.08033845
C	2.05971025	2.19705379	2.33971516
C	3.12882509	3.25649709	2.66644154
C	4.45259232	2.96307031	1.93465991
H	1.43249649	3.03573160	0.45415377
H	3.91791866	3.80619908	0.01037815
H	5.17498009	2.57406263	-0.08745392
H	3.52060608	0.78356712	0.38305499
H	2.97597098	1.74461508	-1.00110879
H	2.37909171	1.21612154	2.72431457
H	1.10452358	2.44533836	2.81526809
C	0.17604882	-3.47408060	-0.13770162
H	0.90477682	-4.28382713	-0.34607267
H	-0.89831615	-3.71798923	-0.27067968
O	0.54464870	-2.36959198	0.22038185

Formaldehyde–5,6-dihydroindolo[2,3-*b*]indole (X = C; R = 3-CF_3-phenyl)

E: −5240.42
H: −5079.49
G: −5127.24
N_{imag} = 0

H	-0.02980627	-1.50621711	-2.94640037
H	0.02980627	1.50621711	-2.94640037
N	0.02286265	1.27013866	-1.96204894
C	0.00000000	0.00000000	-1.44850495
H	0.07231487	4.11426382	-1.79068591
N	-0.02286265	-1.27013866	-1.96204894
F	1.43503106	6.14350358	0.67478496
C	-0.00000000	-0.00000000	-0.04675833
C	-0.04127194	-2.15659986	-0.86542091
C	-0.06502137	-3.54474286	-0.86622881
C	-0.07166312	-4.19535136	0.37673955
C	-0.06128527	-3.46451766	1.58278624
C	-0.03760542	-2.07080693	1.57712805
C	-0.02693506	-1.38354291	0.35262311
H	-0.07231487	-4.11426382	-1.79068591
C	-0.15019841	-5.69547001	0.43172725
H	-0.06602588	-4.00022709	2.52731319
F	0.24472958	-6.29196017	-0.74124187
C	0.04127194	2.15659986	-0.86542091
C	0.02693506	1.38354291	0.35262311
C	0.03760542	2.07080693	1.57712805
C	0.06128527	3.46451766	1.58278624
C	0.07166312	4.19535136	0.37673955
C	0.06502137	3.54474286	-0.86622881
F	-0.62831134	6.22760458	1.43029944
F	-1.43503106	-6.14350358	0.67478496
H	0.06602588	4.00022709	2.52731319
C	0.15019841	5.69547001	0.43172725
F	0.62831134	-6.22760458	1.43029944
F	-0.24472958	6.29196017	-0.74124187
H	-0.02509117	-1.52295109	2.51559483
H	0.02509117	1.52295109	2.51559483
C	-0.00000000	-0.00000000	-5.92144713
O	-0.00000000	-0.00000000	-4.70464001
H	0.01984734	0.94473374	-6.50377182
H	-0.01984734	-0.94473374	-6.50377182

Formaldehyde–5,6-dihydroindolo[2,3-*b*]indole (X = Si; R = 3-CF_3-phenyl)

E: −5119.99
H: −4962.54
G: −5009.57
N_{imag} = 0

H	1.15176733	−2.11499704	0.50850642
H	−0.05384805	−2.70367874	−1.79626166
N	−0.14564053	−1.69146872	−1.86446196
C	0.39226819	−0.83503251	−0.96414815
H	−1.58995061	−2.76898891	−3.92409077
N	0.94855319	−1.15720714	0.22759036
F	−3.02992094	−2.85485506	−5.91698298
Si	−0.03357928	0.96756363	−1.20179091
C	0.82033641	−0.10998757	1.17811975
C	1.34171800	−0.19074534	2.46849750
C	1.09803595	0.86295185	3.35695899
C	0.37343734	1.99346806	2.94420239
C	−0.08904123	2.08498238	1.62967611
C	0.09223424	1.03673309	0.71346512
H	1.91880732	−1.05570275	2.78450768
C	1.56653078	0.75639422	4.78421696
H	0.18768524	2.80043587	3.64682310
F	2.64638577	−0.08049941	4.92534472
C	−1.17329388	−1.08342175	−2.63305192
C	−1.46986687	0.27401391	−2.27189648
C	−2.50289563	0.90639047	−2.98195694
C	−3.23854818	0.22967339	−3.95720395
C	−2.90639911	−1.09248938	−4.29649552
C	−1.85722274	−1.75279323	−3.64676082
F	−4.15773784	−1.00686079	−6.33578671
F	0.58209336	0.25452888	5.61304944
H	−4.05902187	0.72668945	−4.46633274
C	−3.72317929	−1.82641427	−5.32702751
F	1.92836582	1.96833416	5.31165388
F	−4.85622725	−2.39380331	−4.77734795
H	−0.61084528	2.98725552	1.31726548
H	−2.74782373	1.94390562	−2.76386750
C	0.80056224	−5.30826088	−0.04684043
O	0.77331895	−4.12424680	−0.32876559
H	0.38550326	−6.07758069	−0.72996102
H	1.24165989	−5.66848297	0.90522875

Formaldehyde–5,6-dihydroindolo[2,3-*b*]indole (X = Ge; R = 3-CF_3-phenyl)

E: −5090.21
H: −4933.23
G: −4981.34
N_{imag} = 0

H	1.16612582	−2.01294246	0.41722895
H	0.00496706	−2.57946700	−1.80341028
N	−0.07394301	−1.56753059	−1.91496719
C	0.44502596	−0.70365199	−1.02592474
H	−1.44703996	−2.73419737	−3.95842489
N	1.00497844	−1.04118540	0.14818589
F	−2.84453183	−2.85227200	−6.02651743
Ge	−0.01968623	1.23942584	−1.27849367
C	0.86016974	−0.03542238	1.15066554
C	1.41051070	−0.17950830	2.42333830
C	1.15722310	0.81403582	3.37836023
C	0.39618190	1.94349214	3.04532806
C	−0.09850091	2.09239838	1.74409482
C	0.08482727	1.09966666	0.77495249
H	2.02342866	−1.04130547	2.67490884
C	1.66179242	0.63057393	4.78571143

H	0.20197552	2.70403920	3.79593302
F	2.82794038	−0.09442511	4.83634979
C	−1.14332295	−1.01283692	−2.68057604
C	−1.53438163	0.30976750	−2.32179984
C	−2.59791026	0.87316182	−3.03568060
C	−3.29324127	0.14394596	−4.00778695
C	−2.87881532	−1.15452834	−4.33601838
C	−1.78432414	−1.73791857	−3.68384668
F	−4.25165572	−1.17674762	−6.29904134
F	0.75390338	−0.05228236	5.57192870
H	−4.14638041	0.58310994	−4.51646660
C	−3.64356748	−1.95878481	−5.35439542
F	1.90186137	1.81921095	5.42109462
F	−4.64531682	−2.70909312	−4.76920865
H	−0.65379675	2.99464276	1.49433714
H	−2.91067663	1.89370564	−2.82250693
C	0.51565632	−5.16377719	0.06859134
O	0.67606955	−4.01611001	−0.30500981
H	0.01241309	−5.91727258	−0.57127056
H	0.86626165	−5.50411374	1.06441199

Formaldehyde–1,3-bis[4-(trifluoromethyl)phenyl]urea

E: −5378.86
H: −5204.68
G: −5255.22
N_{imag} = 0

C	−0.17277054	−9.40809808	−1.80480641
C	−0.19559895	−10.75888850	−1.48070272
C	−0.21251419	−11.15786236	−0.13660601
C	−0.19974021	−10.18311102	0.86812575
C	−0.17711732	−8.82483828	0.55414633
C	−0.08798720	−3.43732779	−0.79512042
C	−0.07811611	−2.45122367	−1.80480471
C	−0.05526630	−1.10043375	−1.48070033
C	−0.03836599	−0.70146054	−0.13660324
C	−0.05117902	−1.67621181	0.86812815
C	−0.07382303	−3.03448404	0.55414811
H	−0.11587530	−4.98413223	−5.86101604
H	−0.16470137	−9.10465724	−2.85025514
O	−0.12548261	−5.92966119	0.80076348
H	−0.11325023	−4.90650717	−2.22409059
H	−0.17195659	−8.07634373	1.33452122
H	−0.13766984	−6.95281373	−2.22409116
C	−0.12545732	−5.92966428	−5.28098664
C	−0.18374949	−12.61988170	0.21361705
H	−0.03724192	−1.37524623	1.91155091
H	−0.08617067	−2.75466421	−2.85025363
O	−0.12545436	−5.92966062	−4.06416339
C	−0.06710192	0.76055937	0.21362001
H	−0.07901302	−3.78297864	1.33452272
H	−0.21368914	−10.48407705	1.91154820
H	−0.13504183	−6.87519986	−5.86101027
H	−0.04409510	−0.35501207	−2.27040875
H	−0.20673837	−11.50431029	−2.27041150
F	1.10430468	−13.11665947	0.26273760
F	−0.85231732	−13.39146852	−0.70607762
F	−0.74208539	−12.88077371	1.43751838
F	0.49122346	1.02143783	1.43752905
F	−1.35514533	1.25736700	0.26272349
F	0.60149624	1.53213113	−0.70606513
N	−0.10877369	−4.77616625	−1.21600003
N	−0.14216039	−7.08315506	−1.21600069

C	−0.12547441	−5.92966079	−0.42631370
C	−0.16293493	−8.42199389	−0.79512179
